# The noncoding RNAs regulating pyroptosis in colon adenocarcinoma were derived from the construction of a ceRNA network and used to develop a prognostic model

**DOI:** 10.1186/s12920-022-01359-w

**Published:** 2022-09-20

**Authors:** Yanfeng Chen, Zongbiao Tian, Hebin Hou, Wei Gai

**Affiliations:** Department of Gastroenterology, TengZhou Central People’s Hospital, Tengzhou, Shandong China

**Keywords:** Pyroptpsis, Noncoding RNAs, ceRNA network, Colon adenocarcinoma

## Abstract

**Background:**

Noncoding RNAs (ncRNAs), pyroptosis and tumours are all hot topics in current research, but there are very limited studies on pyroptosis and its regulated ncRNAs in colon adenocarcinoma (COAD).

**Methods:**

The COAD transcription profile dataset from TCGA was used for differential expression analysis. Pyroptosis-related genes (PRGs), the top 200 long noncoding RNAs (lncRNAs) and circular RNA (circRNAs) were selected from the results to construct an endogenous competitive RNA (ceRNA) network. Moreover, the expression of the ceRNAs was used for consensus cluster analysis of COAD and developing a risk model after combining clinical follow-up data by the least absolute shrinkage and selection operator method. The stability and independent prognostic ability of the risk model were evaluated. Finally, gene set enrichment analysis (GSEA) and immune score comparisons between the high-risk and low-risk groups were performed.

**Results:**

There were 87 PRGs with significant differences, among which casp3/8, NLRP1/3, and IL-1α/1β were at the core of the interactions. The ceRNA network consisted of 58 lncRNAs, 6 circRNAs, 25 PRGs, and 55 microRNAs. We speculated that KCNQ1OT1-miRNAs-SQSTM1 and HSA_CIRC_0001495-miRNAs-PTEN have great potential and value in the pyroptosis mechanism of COAD. Nine RNAs were involved in the risk score, which had excellent independent prognostic ability. Survival analyses were significant between the high-risk (HR) and low-risk (LR) groups (training cohort: *P* < 0.001; test cohort: *P* = 0.037). GSEA was mainly enriched in tumour proliferation and metastasis related pathways, while differences in immune activity showed a bipolar distribution between the HR and LR groups.

**Conclusions:**

The overall mechanism of pyroptosis in COAD was revealed. CeRNAs most closely related to the pyroptosis mechanism of COAD were selected and used to develop a prognostic model. The results may present new regulatory sites and potential targets for COAD pyroptosis mechanisms.

**Supplementary Information:**

The online version contains supplementary material available at 10.1186/s12920-022-01359-w.

## Introduction

Colon cancer is a very common malignant tumour of the digestive system, and its morbidity and mortality have increased in recent years [1]. Among its subtypes, colon adenocarcinoma (COAD) represents an extremely high proportion (98%) [2]. Currently, the main treatments for COAD include surgery, chemotherapy and immunotherapy. However, within 5 years of treatment, an estimated 30–50% of COAD patients will eventually relapse or metastasize [3]. Due to the progress of surgery and systemic treatment, the overall prognosis of COAD has been greatly improved, but the survival of patients with advanced age, postoperative recurrence or metastasis is still unsatisfactory [4]. Although studies have suggested that the pathogenesis of COAD is related to diet, genetics, chronic enteritis and diverticular disease, the specific mechanism remains to be fully elucidated [5, 6]. Advances in molecular biology have revolutionized the way of understanding diseases. The identification of gene phenotypes for neoplastic diseases plays a crucial role in understanding disease progression and discovering key markers for prognosis or diagnosis [7, 8]. Therefore, we need to continuously use these methods to develop new diagnostic methods and evaluation systems to facilitate and improve individualized treatment regimens.

In recent years, pyroptosis research has attracted the attention of scientists and has become a hot research field. Gasdermin (GSDM) family proteins are cleaved by activated cysteinyl aspartate-specific proteinase (CASP) and then transferred to the cell membrane and polymerized into a pore-like structure, resulting in changes in cell membrane permeability. Secondary cells swell, rupture, and die. Simultaneously, cytokines in the interleukin-1 (IL-1) family are released into the outside of the cell to activate the host immune response, a process known as pyroptosis [9]. Scientists believe that pyroptosis is closely linked to cancer. Recent evidence suggests that pyroptosis induces intense inflammatory responses and significant regression of tumour cells. However, pyroptosis is a double-edged sword, and abnormal pyroptosis may cause organ failure and the formation of a microenvironment conducive to tumour progression [10]. In addition, noncoding RNAs (ncRNAs), such as long noncodinig RNAs (lncRNAs) and circular RNAs (circRNAs), have been proven to have a regulatory effect on the activation of the pyroptosis pathway, but studies are mostly limited to some non-oncological diseases, such as cardiovascular diseases, immune diseases, and diabetic nephropathy [11, 12]. Moreover, the regulatory mechanism of these ncRNAs is likely to affect mRNA expression by acting as competing endogenous RNAs (ceRNAs). The specific process is that response elements on microRNAs (miRNAs) can bind not only to some mRNAs but also to some lncRNAs, circRNAs and other types of RNAs, thus forming a competitive relationship between RNAs that bind the same miRNA [13].

Previous studies have not only found the close association between pyroptosis and a variety of malignant tumours but also found that this mechanism could be regulated by many ncRNAs [14–16]. However, there are still limited studies on pyroptosis and COAD, especially the regulatory mechanism of ncRNA on the pyroptosis activation state of COAD. The Cancer Genome Atlas (TCGA) (https://portal.gdc.cancer.gov/repository) database and some signal pathway-related databases were utilized in this study. LncRNAs and circRNAs with regulatory value in COAD pyroptosis were selected. Moreover, a gene set prognostic scoring model was established based on the ceRNAs. This study not only confirmed the involvement of the pyroptosis pathway in COAD occurrence and progression but also attempted for the first time to identify ceRNAs that interfere with COAD pyroptosis. This work provides a new perspective and target for future research, early warning signs and new therapeutic means.

## Methods

### Data sources and characteristics

Transcriptome sequencing data and clinical follow-up information of COAD patients were downloaded from the TCGA. Workflow Type Select “HTSeq—FPKM” (High-throughput sequence—Fragments Per Kilobase of exon model per Million mapped fragments). The paraffin-embedded sample data were deleted, and the multiple sequencing results of the same patient sample were averaged. The overall survival (OS) data of some patients were lost to follow-up, resulting in an unequal number of cases (Table 1).

**Table 1:** Sample composition and follow-up data of the TCGA COAD patients

Dataset	Specimen category(n)		Follow-up time(days)		OS(n)		Clinical traits(n)								
	Tumor	Tumor-adjacent	Minimum–maximum	Average	Survival	Death	Age(years)		Sex		TNMstage				
							≤ 65	> 65	Female	Male	I	II	III	IV	unkonw
TCGA-COAD	388	39	0–4502	840.83	300	79	157	222	177	202	65	149	101	53	11

*TCGA* The cancer genome atlas; *COAD* Colon adenocarcinoma; *OS* Overall survival

To comprehensively identify pyroptosis-related genes (PRGs), we retrieved “pyroptosis” in two databases: GeneCards (http://www.genecards.org/) and MsigDB (https://www.gsea-msigdb.org/). The filtering conditions of the GeneCards database were set as “Protein Coding RNA” and “Relevance Score > 1”, and 102 PRGs remained. In addition, GOBP_PYROPTOSIS (18 PRGs) and REACTOME_PYROPTOSIS (27 PRGs) were obtained from the MsigDB database. Based on the three gene sets, 117 PRGs (Additional file 1: Table S1) were collected for subsequent analysis.

### Differential expression analysis

The difference in the expression value of the whole transcriptome between paracancer and COAD tissues was analysed using the Mann–Whitney test and the R language "limma" package (cutoff: *P* < 0.05). To avoid missing valuable PRG, the logFoldChange (logFC) threshold was set to zero. The significantly differentially expressed genes of 117 PRGs (dePRGs) were selected and their interactions were determined by the STRING website (https://string-db.org) (interaction score = 0.4) and the “igraph” and “reshape2” packages of R (Pearson, correlation threshold = 0.2). At the same time, differentially expressed lncRNAs (de-lncRNAs), and differentially expressed circRNAs (de-circRNAs) were ranked by |logFC|, the top 200 of which were taken, respectively for the next step. Annotation files downloaded from miRcode (http://mircode.org/) and starBase (https://starbase.sysu.edu.cn/starbase2/).

### Construction of the ceRNA network

The TargetScan (http://www.targetscan.org) database was used to identify miRNAs that can bind the mRNA of dePRGs (mRNAs-TargetScan-miRNAs) (Additional file 1: Table S2). The miRNAs that could bind to the lncRNAs or circRNAs above were further predicted by miRcode and starBase, respectively (Additional file 1: Table S3–S4). The overlapping domains of miRNAs bound by mRNAs and ncRNAs could form a ceRNA network. Therefore, it could be predicted that the ncRNAs in the network may competitively bind these miRNAs to regulate the activity of the pyroptosis pathway. Visual manipulation using Cytocape_V3.9.0.

### Consensus cluster analysis based on ceRNAs

The expression matrix of ceRNA in COAD patients was analysed by the “ConsensusClusterPlus” package of R. The significance of grouping was observed as the clustering variable (K) increased. Then, survival analysis (Kaplan‒Meier method) and differential expression analysis (117 PRGs) were performed among the clusters, demonstrating the effect of ceRNA on pyroptosis activity in COAD.

### Development and establishment of a prognostic risk model

Since the selected ceRNAs were believed to have the ability to regulate the opening of the pyroptosis pathway, a risk scoring formula was constructed based on the expression of the ceRNAs, from which possible key factors and prognostic markers were found, and the interfering effect of pyroptosis on COAD OS was indirectly confirmed. First, TCGA COAD patients were randomly divided into a training cohort and a test cohort. Then, the expression of ceRNAs and follow-up data of patients in the training cohort were merged. Cox regression and least absolute shrinkage and selection operator (LASSO) regression were performed successively, resulting in a risk coefficient for each remaining ceRNA (R: “glmnet” package).

Risk Score = $$\sum \begin{gathered} a \hfill \\ i \hfill \\ \end{gathered} Bi \times Ci$$(The sum of each ceRNA’s FPKM multiplied by the risk coefficient). According to the median Risk score, patients were divided into the High Risk (HR) group (> median score) and Low Risk (LR) group (< median score). Next, the separation efficacy of the risk model and the difference in OS between the two groups were assessed. Principal component analysis (PCA), T-distributed neighbor embedding (T-SNE), Kaplan‒Meier survival analysis, and receiver operator characteristic (ROC) were applied sequentially.

The effectiveness of the risk model was validated based on the data of the test group by the same approach and process.

### Assessment of the independent prognostic strength of the risk model

All COAD patients were analysed using risk scores combined with clinical characteristics, including age, sex, TNM grading and tumour stage. First, factors that were not significantly correlated with OS were excluded by univariate Cox regression analysis. Second, multivariate Cox regression analysis was performed to identify factors that had independent evaluation value for prognosis, and the role of the risk model was considered.

### Gene set enrichment analysis

To understand the functional pathways that may promote the progression of COAD or even have a close relationship with the activity of pyroptosis, gene set enrichment analysis (GSEA) was used for differential pathway enrichment between the HR and LR groups. The classic HALLMARK gene sets were used in GSEA_4.1.0 software (enrichment statistic: weighted; metric for ranking genes: single2noise; cut-off: |NES|> 1, NOM *P* value < 0.05, FDR q-value < 0.25).

### Differences in immune activity between the HR and LR groups

The immune-related gene set was downloaded from the GSEA website (https://www.gsea-msigdb.org/gsea/index.jsp) and was enriched in 13 types of immune cells and 16 immune function pathways. From this gene set, single-sample gene set enrichment analysis (ssGSEA) was used to score the immunity of each COAD sample, thus more intuitively showing differences in immune activity between the HR and LR groups.

### Statistical analysis

In this study, R × 64 4.1.1 was used for data analysis, and functions and thresholds for key steps were listed in each section.

## Results

### PRGs differentially expressed in COAD

**Fig. 1:**
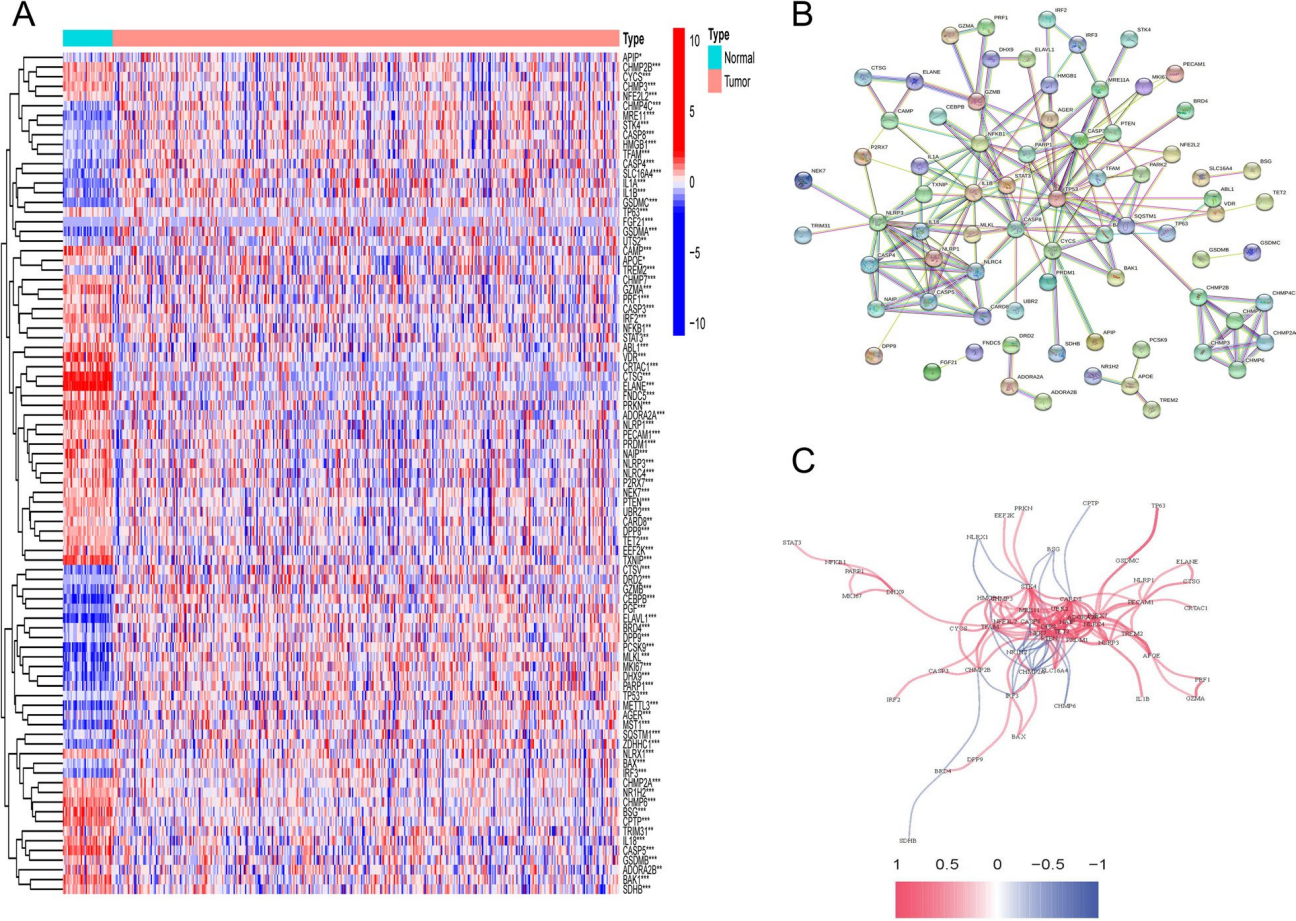
Differential expression and interaction of PRGs in COAD. **A** Expression of differential PRGs between tumor and normal samples (Each cell represents the log2FPKM of genes; brilliant blue: normal samples; cherry red: tumor samples; blue-red: log2FPKM gradually increased; *P*-Values were displayed as: *P < 0.05; **P < 0.01; ***P < 0.001). **B** Interaction network of dePRGs by String (eachedge represents an interactive path). **C** The correlations of dePRGs by Pearson correlation analysis (blue line: negative correlation; red line: positive correlation; the depth of the line color indicates the strength of the correlation)

A total of 87 PRGs were significantly differentially expressed (Fig. 1A). The most interacting genes were at the core of the STRING network (Fig. 1B). Moreover, there were also different levels of positive and negative regulation between genes (Fig. 1C).

### CeRNA network

**Fig. 2:**
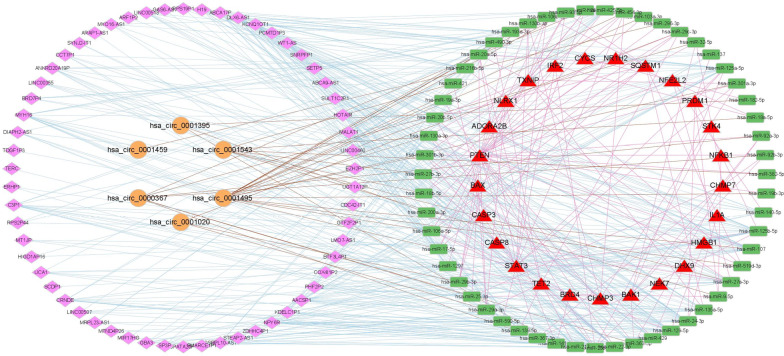
The de-lncRNAs and de-circRNAs can combine the miRNAs competitively with dePRGs in COAD. (yellow circle: de-circRNAs; pink diamond: de-lncRNAs; red triangle: de-PRGs; green rectangle: microRNAs; blue edges: interactions between lncRNAs and miRNAs; brown edges: interactions between circRNAs and miRNAs; light red line: interactions between PRGs and miRNAs)

A total of 58 lncRNAs and 6 circRNAs were found to compete with 25 PRGs to bind 55 miRNAs (Fig. 2). The 64 ceRNAs could likely regulate the pyroptosis activity of COAD, the expression of which was used to construct a risk model.

### Consensus cluster analysis

**Fig. 3:**
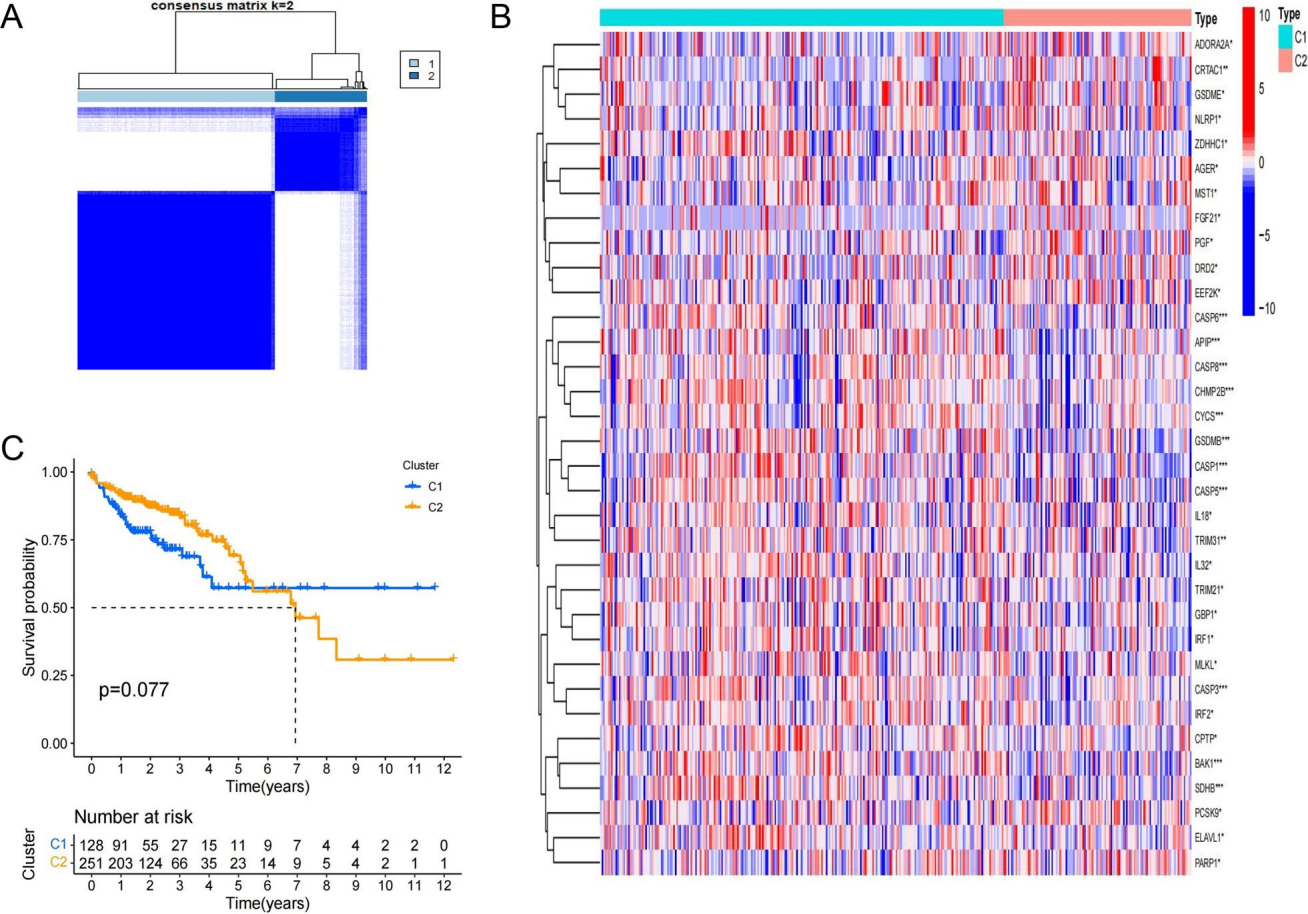
The differences of pyroptosis activity and OS between two clusters based on ceRNAs expression. **A** 379 COAD patients were divided into two clusters (C1, C2) by consensus cluster analysis (k = 2). **B** Differential expression of 117 PRGs between C1 and C2 (brilliant blue: C1; cherry red: C2; blue-red: the expression level of logFPKM; *P*-Values were shown as: *P < 0.05, **P < 0.01, ***P < 0.001). **C** OS curves for two clusters (cluster C1: blue; cluster C2: yellow)

Two relatively clear and stable clusters were obtained (Fig. 3A), and 34 PRGs were significantly differentially expressed between the two clusters, including several universally recognized hub genes of the pyroptosis pathway in the CASP family and GSDM family (Fig. 3B). However, there were no significant differences in OS (Fig. 3C). The results showed that clusters based on the coexpression levels of predictive regulatory genes in the ceRNA network did show significant differences and had different degrees of pyroptosis.

### Risk model of the training cohort

**Fig. 4:**
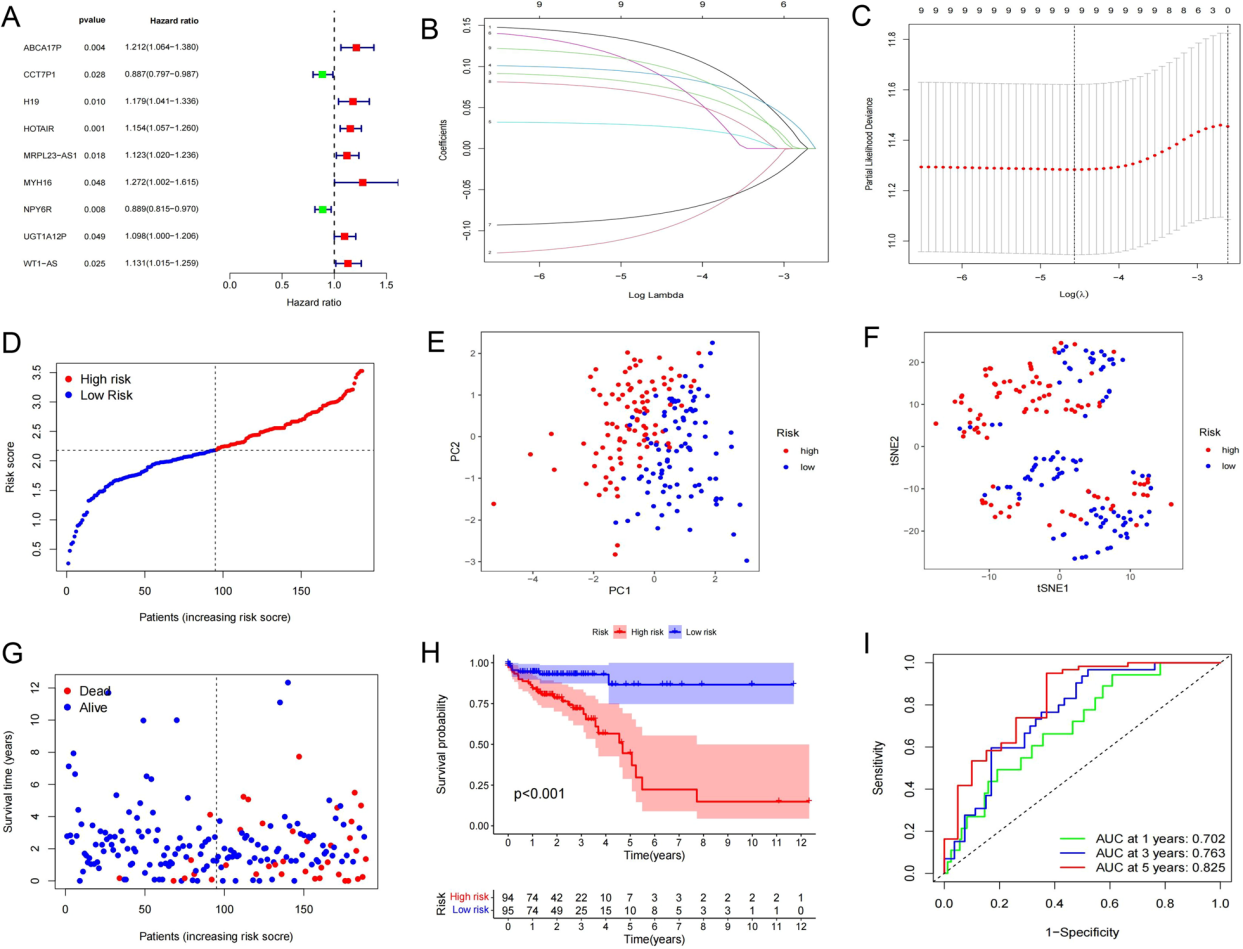
Exploration of risk model in the train cohort. **A** 9 ceRNAs had significant correlation with OS by univariate cox regression analysis (*P* < 0.05). **B** LASSO regression of the 9 ceRNAs. **C** Cross-validation of optimal adjustment of λ-value. **D** Patients distribution in the HR and LR groups. **E** PCA of HR and LR groups. **F** t-SNE plot of HR and LR patients. **G** The survival state of train cohort patients. **H** OS curves of HR and LR groups (*P* < 0.001). **I** Prediction efficiency of the risk model for three time gradients

After Cox regression analysis, the remaining 9 ceRNAs related to OS were all lncRNAs. Among them, MYH16 and ABCA17P had large hazard ratios (HRs). In contrast, the HRs of CCT7P1 and NPY6R were less than 1, suggesting that they may inhibit tumour progression (Fig. 4A). According to the result of the optimum λ value, the 9 genes could not be reduced by LASSO regression analysis (Fig. 4B, C), and the formula for the risk score of the 9 genes was as follows:risk score = (0.125 × ABCA17P) + (− 0.100 × CCT7P1) + (0.077 × H19) + (0.090 × HOTAIR) + (0.027 × MRPL23-AS1) + (0.092 × MYH16) + (− 0.080 × NPY6R) + (0.065 × UGT1A12P) + (0.101 × WT1-AS). According to the median score, COAD patients were evenly divided into HR and LR groups (Fig. 4D), and the two groups were clearly separated by PCA and t-SNE (Fig. 4E, F). Visualization of the survival state showed that patients who died and had a short survival period were more concentrated in the high score segment (Fig. 4G), and OS analysis showed significant differences (*P* < 0.001) (Fig. 4H). In addition, ROC analysis results indicate that the risk model has a satisfactory ability to evaluate survival expectation at three nodes of time (1, 3 and 5 years) (Fig. 4I).

### Risk model testing

**Fig. 5:**
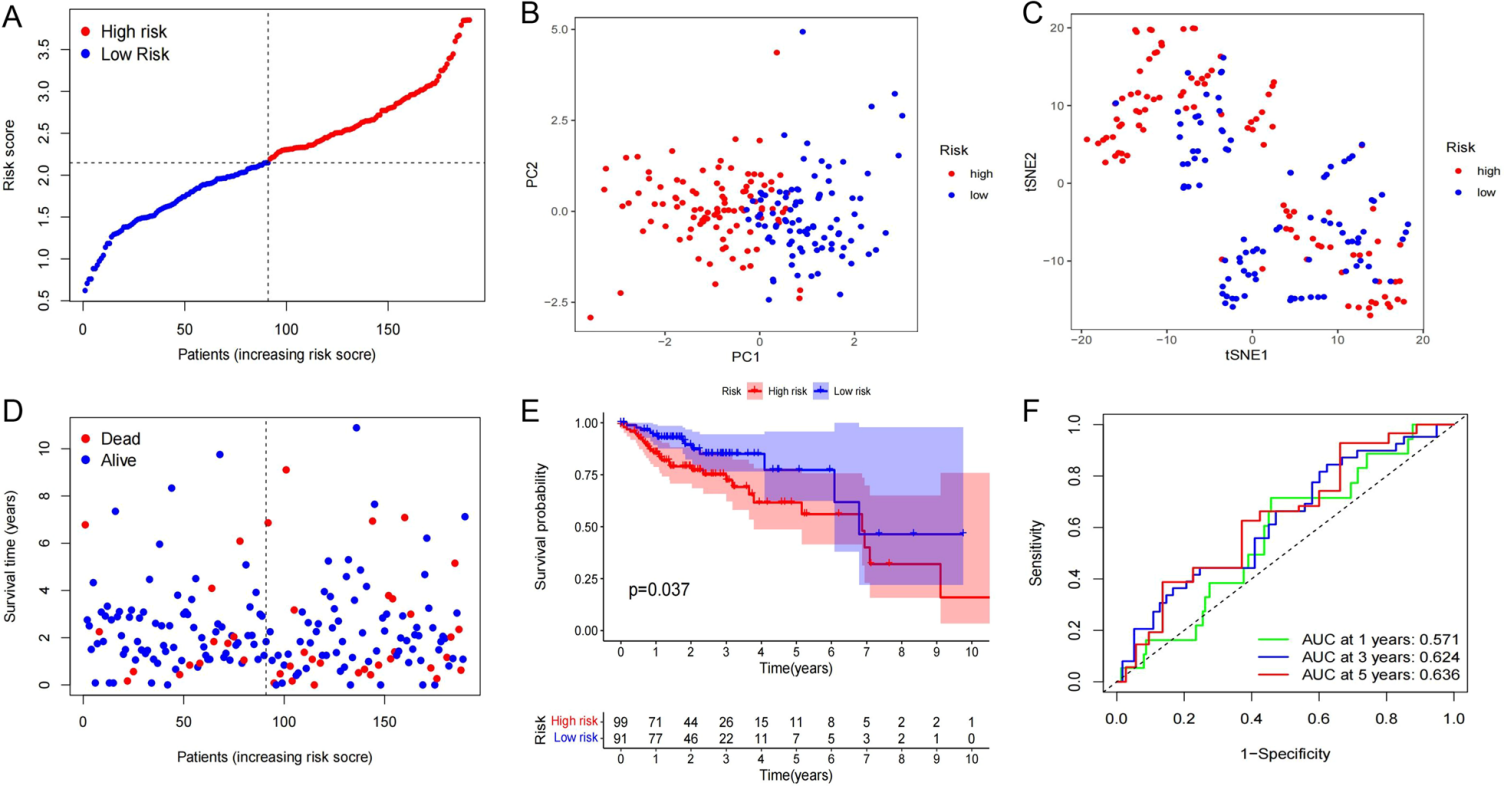
Validation of prognostic model by test cohort. **A** Patients distribution based on the median score. **B** PCA of HR and LR groups in test cohort. **C** t-SNE plot for HR and LR groups of test cohort. **D** Survival status of test cohort patients. **E** OS curves of HR and LR groups (*P* = 0.037). **F** Time-dependent ROC curves for test cohort patients

To verify the reliability of the risk model, the test cohort with an equal number of randomly separated samples from the total COAD patients was analysed according to the same process. The result was slightly inferior to that of the training cohort. However, the number of HR (99) and LR (91) cases were similar, and the risk score presented a linear distribution (Fig. 5A). In PCA, the distribution difference between the two groups was clear (Fig. 5B), while t-SNE results showed weak differentiation (Fig. 5C). The survival situation suggested that the number of deaths in areas with high scores was still higher (Fig. 5D). Importantly, OS analysis remained significant (*P* = 0.037) (Fig. 5E) and the areas under the curves (AUCs) were 0.571, 0.624, 0.636 in 1, 3, 5 years, respectively (Fig. 5F).

### Independent prognostic power of the risk model

**Fig. 6:**
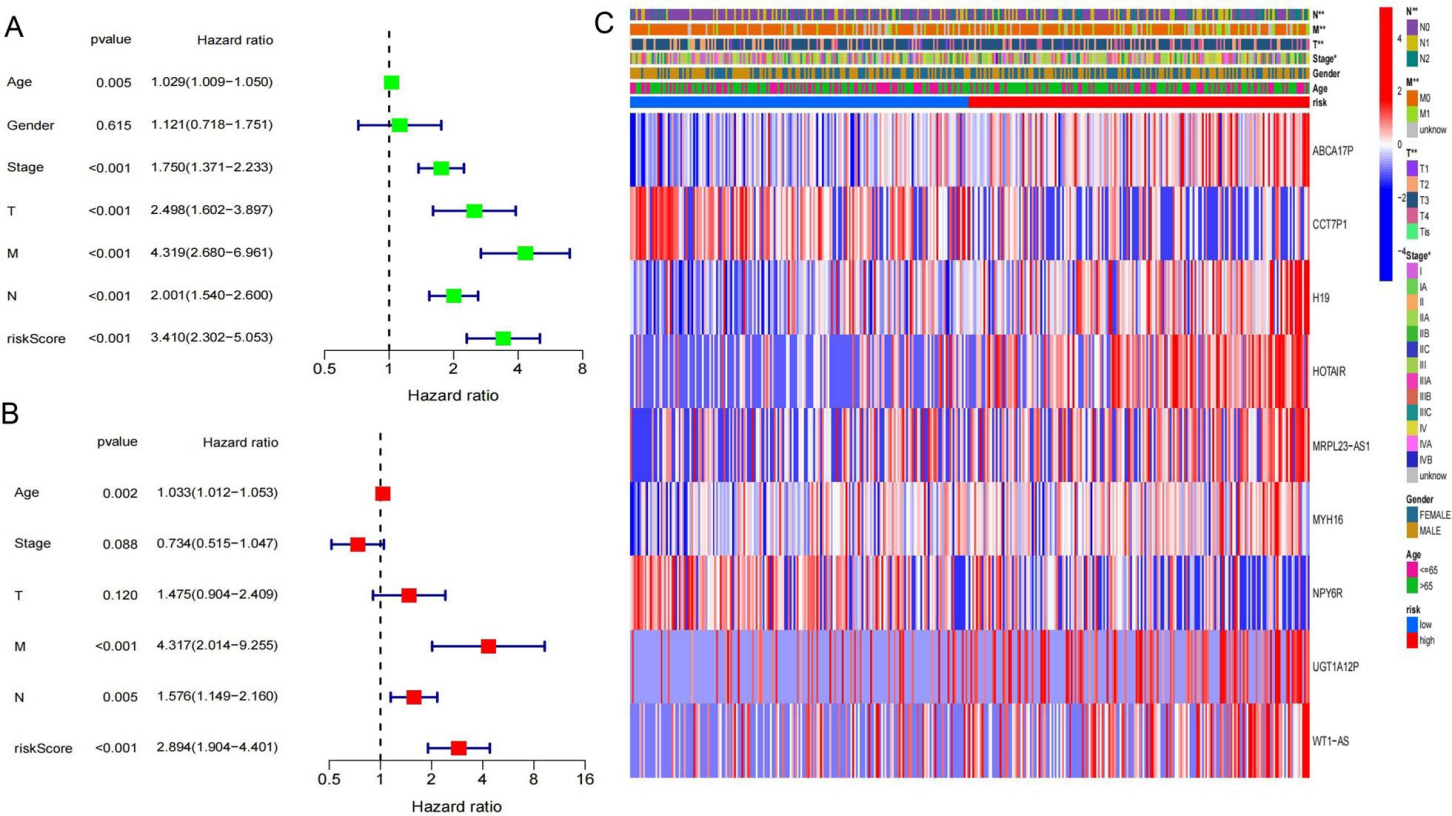
Independent prognostic assessment of clinical traits and risk score. **A** Univariate analysis for all of the TCGA COAD patients (*P* < 0.05 indicates a significant relationship with OS). **B** Multivariate analysis after adjusting confounding factors (Age, N&M grading, riskScore had independent prognostic ability). **C** Distribution significance of various clinical traits in the HR and LR groups (*P < 0.05, **P < 0.01)

M stage and risk score showed a close relationship with OS and had independent prognostic ability, while T stage and sex had no independent prognostic value (Fig. 6A, B). The survival of patients diagnosed with advanced COAD is naturally predictable, so the strong predictive power of the risk score is of clinical value. According to the heatmap (Fig. 6C), patients of different stages and grades were significantly different in the distribution of HR and LR groups, which confirmed the credibility of risk stratification.

### Differences in GSEA between HR and LR groups

**Fig. 7:**
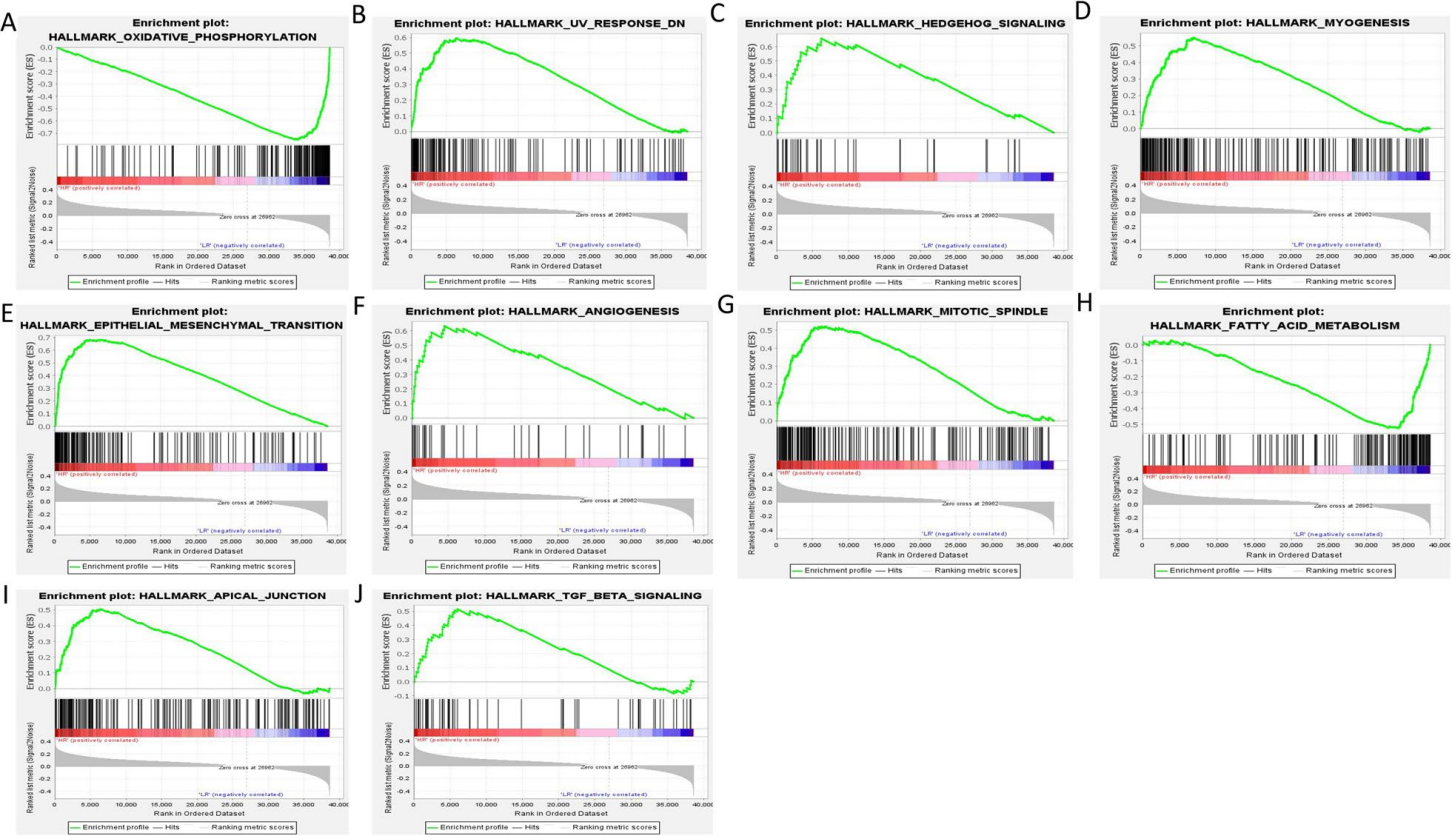
Top 10 enriched pathways between HR and LR groups of TCGA samples. (**A**–**J** |NES|gradually reduce)

A total of 13 pathways were significantly enriched (Additional file 1: Table S5). We show the top 10 pathways according to |NES| sorting. These are “OXIDATIVE_PHOSPHORYLATION”, “UV_RESPONSE_DN”, “HEDGEHOG_SIGNALING”, “MYOGENESIS”, “EPITHELIAL_MESENCHYMAL_TRANSITION”, “ANGIOGENESIS”, “MITOTIC_SPINDLE”, “FATTY_ACID_METABOLISM”, “APICAL_JUNCTION”, and “TGF_BETA_SIGNALING” (Fig. 7A–J). Most of the above pathways are considered to be closely related to tumour progression, which reflects the reliability of grouping.

### Differences in immune activity between the HR and LR groups

**Fig. 8:**
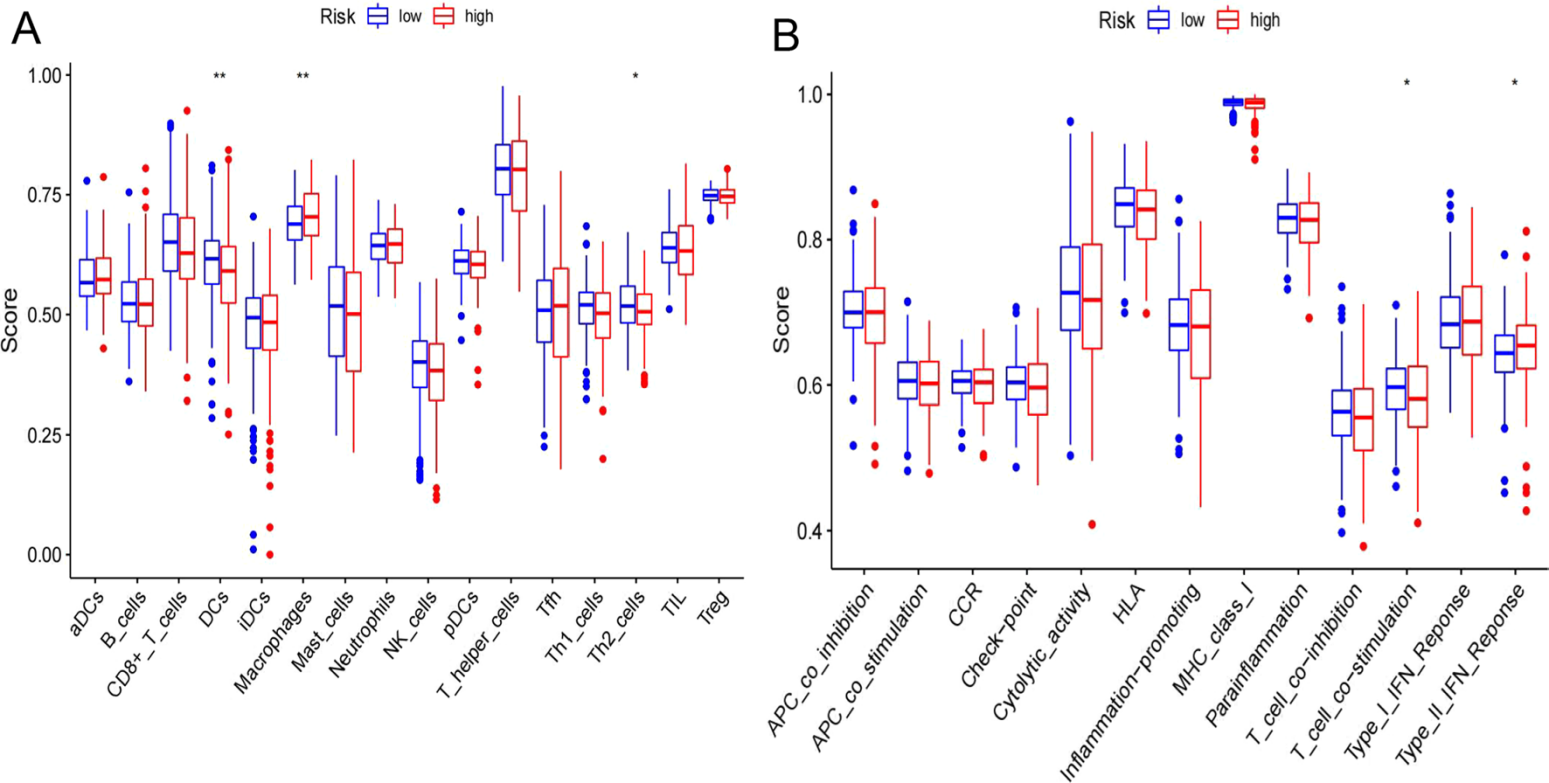
Difference of immune activity between HR and LR groups of TCGA data set. **A** Comparison of 16 types of immune cells between LR and HR groups. **B** Comparison of 13 types of immune functions between LR and HR groups (*P < 0.05; **P < 0.01; HR: red box; LR: blue box)

The comparison of immune scores between all HR and LR samples can be seen by box plot (Fig. 8A, B). The activity of dendritic cells (DCs) (*P* < 0.01) and T helper 2 (Th2) cells (*P* < 0.05) was decreased in the HR group, while the activity of macrophages (*P* < 0.01) was increased significantly. In addition, “T_cell_co-stimulation” (*P* < 0.05) was inhibited in the HR group, while “Type_II_IFN_Response” (*P* < 0.05) was overactivated.

## Discussion

Research on the correlation relationship between the pyroptosis pathway and tumours is a hot topic today [17]. Current studies mostly show that pyroptosis may promote cancer progression and metastasis [18, 19]. However, the opposite conclusion has also been reached: pyroptosis can sometimes prevent the development of anticancer drug resistance and even prevent tumour progression [20, 21]. Some researchers have found that pyroptosis is regulated by some ncRNAs in the process of tumour growth and metastasis [22]. In summary, pyroptosis and cancer must be closely related and may even become potential biological markers or targets for tumour diagnosis and treatment. Our study was inspired by these studies. In this work, we not only explored the relationship between COAD and pyroptosis more thoroughly but also identified ceRNAs that may play a regulatory role in this process.

First, we selected PRGs from three databases for differential analysis to avoid omission, and 87 PRGs most closely related to COAD were identified. Among them, several CASP family genes including CASP3/4/5/8, had significant differences in expression. Moreover, GSDMA/B/C of GSDM family genes, as the final link of pyroptosis, were also selected. This was consistent with a previous study showing that some chemotherapy drugs can induce pyroptosis in colon cancer cells by CASP3 cleavage of GSDME [23]. Another study suggested that GSDMB was involved in the progression of inflammatory bowel disease, which may explain some of the pathogenesis links between IBD and colon cancer [24]. From the interaction of the 87 PRGs, it was found that casp3/8, NLRP1/3, IL-1α/1β and other hot genes were indeed in the core position, reflecting the reliability of the detection results of the pyroptosis pathway. At the same time, BSG, CHMP2A and other genes may have important negative regulatory effects on pyroptosis, which needs further experimental verification.

NcRNAs has been used as prognostic markers in colon cancer. A recent study found that has_circ_0084927 has prognostic value in colorectal cancer and has an endogenous competition mechanism [25, 26]. The effect of KCNQ1OT1 on lncRNAs in the development of colon cancer has been confirmed in several studies [27, 28]. Our ceRNA network also proved this point. KCNQ1OT1, with the most regulated connections in the network, competes with 14 PRGs, including PTEN/SQSTM1/TXNIP/STAT3, to bind multiple miRNAs. SQSTM1 interferes with the progression of colon cancer through the activation of autophagy [29]. Moreover, SQSTM1 can participate in the degradation of GSDMD and intervene in the activation of the pyroptosis pathway [30]. Therefore, it is reasonable to speculate that KCNQ1OT1-mirNAs-SQSTM1 play an important role in the activation of pyroptosis in colon cancer. Has_circ_0001495 of the ceRNA network has up to 16 possible regulatory routes, among which the interaction with PTEN is the closet. At present, studies on the correlation between hsa_circ_0001495 and hepatocellular carcinoma, cervical cancer, lung cancer and other malignant tumours can be retrieved, suggesting that hsa_circ_0001495 plays a key regulatory role in cancer progression [31–33]. A recent study on oral cancer indicated that the expression of GSDME in oral cancer mice with PTEN knockout genes was significantly increased at an early stage, thereby inhibiting tumour progression by activating the pyroptosis response [34]. Therefore, we once again boldly speculated that hsa_circ_0001495-miRNAs-PTEN might be involved in pyroptosis activation in early colon cancer. Of course, there must be many important regulatory mechanisms in the network, and our interpretation of this result is conducive to providing clues and support for further research.

The clarity of the two clusters generated by consensus cluster analysis led us to believe that the regulatory role of ceRNAs was solid and powerful. The differential expression analysis of PRGs further confirmed the great influence of regulatory genes on the activation state of pyroptosis because the 34 differential PRGs contained multiple CASP and GSDM family members, which are at the core of the pyroptosis pathway.

All 9 ceRNAs in the risk model are lncRNAs. We believe that this phenomenon may be due to some particularities of circRNA, resulting in insignificant differences in their expression levels. However, the prognosis of the risk model still showed good performance in both cohorts. This not only is advantageous for making the right judgement for COAD patients in the clinic but also further confirms the importance of pyroptosis in the progression of COAD. In addition, these ceRNAs also have the potential to be therapeutic targets and the basis of new drug research and development. Among the 9 RNAs, ABCA17P, CCT7P1 and UGT1A12P belong to pseudogenes, which are similar but different from protein-encoding genes in structure, leading to failure to translate proteins. However, they may be functional, and they may act as regulators in a mannersimilar to ncRNAs. There are some limited reports about the relationship between pseudogenes and colon cancer [35, 36]. MYH16 stands out in the risk model. As early as 2002, Nada al-Tassan et al. found that MYH was associated with genetic susceptibility to colorectal cancer [37]. However, there are no studies on MYH16. H19 is a lncRNA with a high frequency in colon cancer related studies. For example, a report from a few years ago showed that H19 promoted the transformation process of colon cancer epithelial cells into mesenchymal cells through the H19/Mir-29B-3p/PGRN Axis [38]. Another study published this year linked H19 overexpression to colon cancer recurrence [39]. At the same time, H19 has also been proven to be closely related to pyroptosis by a number of studies, including pneumonia, myocardial infarction and other diseases [40, 41]. HOTAIR is also a popular lncRNA, and a number of studies have proven that it can promote the progression of colon cancer by regulating the activity of protein-coding RNAs through an endogenous competition mechanism [42, 43]. MRPL23-AS1 has been shown to promote metastasis in some types of lung cancer, but its association with colon cancer has not been identified [44]. There are few studies on the other genes, but some direct or indirect evidence shows that they are related to cancer or pyroptosis pathway. Joint efforts of the scientific community are still needed to explore their unknow roles. In conclusion, the 9 lncRNAs mentioned above showed a possible close relationship with colon cancer or pyroptosis in previous studies and showed a good prognostic prediction of colon cancer outcome with both sensitivity and specificity through mutual restriction or enhancement. The HRs suggested that the independent prognostic efficacy of the risk score was more accurate than N/M staging and was more appropriate for patients in early stages.

Later, we conducted GSEA to understand the difference in pathway enrichment between the HR and LR groups and to preliminarily explore pathways that may be closely related to pyroptosis. Most of the results were recognized as pathways strongly linked to tumorigenesis and development, including vascular, musculogenesis, epithelial mesenchymal cell transformation, transforming growth factor-β (TGF-β) signalling and other functional pathways, which confirmed the credibility of our study [45, 46]. The comparison of immune activity between the different risk layers was interesting. Overall, the difference in immune activity between the two groups was not as great as we thought. In detail, DCs and Th2 cells showed the opposite status to macrophages. In the comparison of immune function, activation of the type II interferon pathway and inhibition of the T-cell costimulation pathway in the HR group indicated that excessive inflammation of colon cells may be involved in the occurrence of cancer, while the progression and deterioration of cancer may be related to the weakening of immune surveillance function. This "contradiction" actually matches the current research status [47, 48].

## Conclusions

In summary, our study preliminarily explored the correlation between COAD and pyroptosis and identified some RNAs that may regulate this process through endogenous competition mechanisms. According to previous studies, these RNAs are tightly linked to COAD, pyroptosis or other malignant tumours. Subsequently, we constructed a risk model based on the expression of the RNAs and clinical follow-up data. The evaluation effect of the training cohort and test cohort was considerable, and the independent prognostic ability was also strong. The enrichment pathways between the HR and LR groups were mainly reflected in the factors related to tumour progression, while the comparison of immune activity displayed a bipolar state. Limitations: In our study, bioinformatics was used to conduct secondary mining of published data, without targeted experimental validation, and the conclusions were not absolutely confirmed. However, the limited scope of the results provides direction and foundation for future research and avoids the economic and time cost of in-depth research. Moreover, the integration of complex mathematical operations and basic life science experiments is considered to be mainstream medical research today and has the potential to yield outstanding research results.

## Supplementary Information

**Additional file 1: Table S1.** There were 117 PRGs left after merging and removing duplicates. **Table S2.** 38 out of 87 dePRGs have corresponding miRNAs binding. **Table S3.** 68 out of top 200 de-lncRNAs have corresponding miRNAs binding. **Table S4.** 8 out of top 200 de-circRNAs have corresponding miRNAs binding. **Table S5.** Enrichment pathways between high and low risk groups results from GSEA

**Additional file 2: Table S6.** Accession numbers of COAD from TCGA database.

## Data Availability

The datasets analysed during the current study are available in the TCGA repository: [https://portal.gdc.cancer.gov/repository], which are publicly available databases. The specific sample is TCGA-COAD, and the detailed accession numbers are shown in Additional file 2: Table S6.

## Abbreviations

ncRNAs: Noncoding RNAs
COAD: Colon adenocarcinoma
PRGs: Pyroptosis-related genes
lncRNAs: Long noncoding RNAs
circRNAs: Circular RNA
ceRNA: Endogenous competitive RNA
LASSO: Least absolute shrinkage and selection operator
GSEA: Gene set enrichment analysis
HR: High-risk
LR: Low-risk
GSDM: Gasdermin
CASP: Cysteinyl aspartate-specific proteinase
IL-1: Interleukin-1
miRNAs: MicroRNAs
TCGA: The cancer genome atlas
FPKM: Fragments per kilobase of exon model per million mapped fragments
OS: Overall survival
PRGs: Pyroptosis-related genes
logFC: LogFoldChange
dePRGs: Differentially expressed PRGs
de-lncRNAs: Differentially expressed lncRNAs
de-circRNAs: Differentially expressed circRNAs
PCA: Principal component analysis
T-SNE: T-distributed neighbor embedding
ROC: Receiver operator characteristic
ssGSEA: Single-sample gene set enrichment analysis
HRs: Hazard ratios
AUCs: Areas under the curves
DCs: Dendritic cells
TGF-β: Transforming growth factor-β
