# Supplementary material for: The noncoding RNAs regulating pyroptosis in colon adenocarcinoma were derived from the construction of a ceRNA network and used to develop a prognostic model
Source: BMC Med Genomics. 2022 Sep 20;15:201. doi: 10.1186/s12920-022-01359-w (PMC9490888; doi:10.1186/s12920-022-01359-w)
Supplement: Supplementary file 1 — Additional file 1: Table S1. There were 117 PRGs left after merging and removing duplicates. Table S2. 38 out of 87 dePRGs have corresponding miRNAs binding. Table S3. 68 out of top 200 de-lncRNAs have corresponding miRNAs binding. Table S4. 8 out of top 200 de-circRNAs have corresponding miRNAs binding. Table S5. Enrichment pathways between high and low risk groups results from GSEA [file 12920_2022_1359_MOESM1_ESM.docx]

**Table S1 There were 117 PRGs left after merging and removing duplicates.**

| 117 PRGs |
| --- |
| AIM2 |
| APIP |
| BAK1 |
| BAX |
| CASP1 |
| CASP3 |
| CASP4 |
| CASP5 |
| CASP8 |
| CHMP2A |
| CHMP2B |
| CHMP3 |
| CHMP4A |
| CHMP4B |
| CHMP4C |
| CHMP6 |
| CHMP7 |
| CYCS |
| DHX9 |
| ELANE |
| GSDMA |
| GSDMB |
| GSDMC |
| GSDMD |
| GSDME |
| GZMA |
| GZMB |
| HMGB1 |
| IL18 |
| IL1A |
| IL1B |
| IRF1 |
| IRF2 |
| NAIP |
| NLRC4 |
| NLRP1 |
| NLRP9 |
| TP53 |
| TP63 |
| ZBP1 |
| NLRP3 |
| CARD8 |
| DPP8 |
| DPP9 |
| PYCARD |
| STAT3 |
| TREM2 |
| FOXO3 |
| CASP6 |
| TXNIP |
| DDX3X |
| SIRT1 |
| GBP1 |
| GJA1 |
| CPTP |
| UBR2 |
| PRDM1 |
| BRD4 |
| CRTAC1 |
| IKBKE |
| NEK7 |
| PCSK9 |
| VDR |
| AGER |
| CTSV |
| NFE2L2 |
| TET2 |
| UTS2 |
| MLKL |
| APOE |
| CD274 |
| EEF2K |
| FGF21 |
| NFKB1 |
| P2RX7 |
| SDHB |
| BSG |
| CEBPB |
| IL32 |
| NLRX1 |
| PGF |
| SLC16A4 |
| TFAM |
| MALT1 |
| ELAVL1 |
| MST1 |
| PRF1 |
| PTGS2 |
| STK4 |
| ABL1 |
| CDK9 |
| HDAC6 |
| IRF3 |
| SQSTM1 |
| STING1 |
| ZDHHC1 |
| ADORA1 |
| ADORA2A |
| ADORA2B |
| ADORA3 |
| DRD2 |
| METTL3 |
| PECAM1 |
| PTEN |
| TRIM31 |
| IFI16 |
| CAMP |
| FNDC4 |
| FNDC5 |
| MRE11 |
| GBP5 |
| PARP1 |
| PRKN |
| TRIM21 |
| NR1H2 |
| CTSG |
| MKI67 |

**Table S2 38 out of 87 de-PRGs have corresponding miRNAs binding.**

| mRNA | miRNA |
| --- | --- |
| EEF2K | hsa-let-7a-5p |
| TP53 | hsa-let-7a-5p |
| CASP3 | hsa-let-7a-5p |
| EEF2K | hsa-let-7b-5p |
| TP53 | hsa-let-7b-5p |
| CASP3 | hsa-let-7b-5p |
| EEF2K | hsa-let-7c-5p |
| TP53 | hsa-let-7c-5p |
| CASP3 | hsa-let-7c-5p |
| EEF2K | hsa-let-7d-5p |
| TP53 | hsa-let-7d-5p |
| CASP3 | hsa-let-7d-5p |
| EEF2K | hsa-let-7e-5p |
| TP53 | hsa-let-7e-5p |
| CASP3 | hsa-let-7e-5p |
| EEF2K | hsa-let-7f-5p |
| TP53 | hsa-let-7f-5p |
| CASP3 | hsa-let-7f-5p |
| EEF2K | hsa-let-7g-5p |
| TP53 | hsa-let-7g-5p |
| CASP3 | hsa-let-7g-5p |
| EEF2K | hsa-let-7i-5p |
| TP53 | hsa-let-7i-5p |
| CASP3 | hsa-let-7i-5p |
| NEK7 | hsa-miR-101-3p |
| TET2 | hsa-miR-101-3p |
| PTEN | hsa-miR-103a-3p |
| TXNIP | hsa-miR-106a-5p |
| PTEN | hsa-miR-106a-5p |
| STAT3 | hsa-miR-106a-5p |
| TET2 | hsa-miR-106a-5p |
| SQSTM1 | hsa-miR-106a-5p |
| TXNIP | hsa-miR-106b-5p |
| PTEN | hsa-miR-106b-5p |
| STAT3 | hsa-miR-106b-5p |
| SQSTM1 | hsa-miR-106b-5p |
| PTEN | hsa-miR-107 |
| STK4 | hsa-miR-124-3p |
| UBR2 | hsa-miR-124-3p |
| STAT3 | hsa-miR-125a-5p |
| BAK1 | hsa-miR-125a-5p |
| PRDM1 | hsa-miR-125a-5p |
| STAT3 | hsa-miR-125b-5p |
| BAK1 | hsa-miR-125b-5p |
| PRDM1 | hsa-miR-125b-5p |
| NEK7 | hsa-miR-1271-5p |
| TXNIP | hsa-miR-128-3p |
| ADORA2B | hsa-miR-128-3p |
| CASP8 | hsa-miR-128-3p |
| HMGB1 | hsa-miR-129-5p |
| TET2 | hsa-miR-129-5p |
| PTEN | hsa-miR-1297 |
| TET2 | hsa-miR-1297 |
| TXNIP | hsa-miR-130a-3p |
| PTEN | hsa-miR-130a-3p |
| STAT3 | hsa-miR-130a-3p |
| CHMP3 | hsa-miR-130a-3p |
| TXNIP | hsa-miR-130b-3p |
| PTEN | hsa-miR-130b-3p |
| STAT3 | hsa-miR-130b-3p |
| CHMP3 | hsa-miR-130b-3p |
| ELAVL1 | hsa-miR-133a-3p |
| ELAVL1 | hsa-miR-133b |
| TXNIP | hsa-miR-135a-5p |
| SQSTM1 | hsa-miR-135a-5p |
| TXNIP | hsa-miR-135b-5p |
| SQSTM1 | hsa-miR-135b-5p |
| PRDM1 | hsa-miR-137 |
| CASP3 | hsa-miR-138-5p |
| TET2 | hsa-miR-138-5p |
| TET2 | hsa-miR-139-5p |
| NFE2L2 | hsa-miR-140-5p |
| PTEN | hsa-miR-141-3p |
| BRD4 | hsa-miR-141-3p |
| TET2 | hsa-miR-143-3p |
| NEK7 | hsa-miR-144-3p |
| PTEN | hsa-miR-144-3p |
| NFE2L2 | hsa-miR-144-3p |
| TET2 | hsa-miR-144-3p |
| TET2 | hsa-miR-145-5p |
| PRDM1 | hsa-miR-145-5p |
| TXNIP | hsa-miR-148a-3p |
| PTEN | hsa-miR-148a-3p |
| TXNIP | hsa-miR-148b-3p |
| PTEN | hsa-miR-148b-3p |
| CHMP7 | hsa-miR-149-5p |
| TXNIP | hsa-miR-152-3p |
| PTEN | hsa-miR-152-3p |
| PTEN | hsa-miR-153-3p |
| NFE2L2 | hsa-miR-153-3p |
| IRF2 | hsa-miR-153-3p |
| CEBPB | hsa-miR-155-5p |
| TXNIP | hsa-miR-15a-5p |
| TXNIP | hsa-miR-15b-5p |
| TXNIP | hsa-miR-16-5p |
| TXNIP | hsa-miR-17-5p |
| PTEN | hsa-miR-17-5p |
| STAT3 | hsa-miR-17-5p |
| SQSTM1 | hsa-miR-17-5p |
| NEK7 | hsa-miR-181a-5p |
| PTEN | hsa-miR-181a-5p |
| STAT3 | hsa-miR-181a-5p |
| CHMP3 | hsa-miR-181a-5p |
| IL1A | hsa-miR-181a-5p |
| NEK7 | hsa-miR-181b-5p |
| PTEN | hsa-miR-181b-5p |
| STAT3 | hsa-miR-181b-5p |
| CHMP3 | hsa-miR-181b-5p |
| IL1A | hsa-miR-181b-5p |
| NEK7 | hsa-miR-181c-5p |
| PTEN | hsa-miR-181c-5p |
| STAT3 | hsa-miR-181c-5p |
| CHMP3 | hsa-miR-181c-5p |
| IL1A | hsa-miR-181c-5p |
| NEK7 | hsa-miR-181d-5p |
| PTEN | hsa-miR-181d-5p |
| STAT3 | hsa-miR-181d-5p |
| CHMP3 | hsa-miR-181d-5p |
| IL1A | hsa-miR-181d-5p |
| CYCS | hsa-miR-182-5p |
| DPP8 | hsa-miR-183-5p |
| TXNIP | hsa-miR-186-5p |
| PTEN | hsa-miR-186-5p |
| HMGB1 | hsa-miR-186-5p |
| DPP9 | hsa-miR-186-5p |
| CYCS | hsa-miR-186-5p |
| NR1H2 | hsa-miR-18a-5p |
| IRF2 | hsa-miR-18a-5p |
| NR1H2 | hsa-miR-18b-5p |
| IRF2 | hsa-miR-18b-5p |
| PTEN | hsa-miR-193a-3p |
| PTEN | hsa-miR-193b-3p |
| TXNIP | hsa-miR-195-5p |
| EEF2K | hsa-miR-196a-5p |
| ABL1 | hsa-miR-196a-5p |
| EEF2K | hsa-miR-196b-5p |
| ABL1 | hsa-miR-196b-5p |
| DPP8 | hsa-miR-199a-5p |
| STK4 | hsa-miR-199a-5p |
| TET2 | hsa-miR-199a-5p |
| DPP8 | hsa-miR-199b-5p |
| STK4 | hsa-miR-199b-5p |
| TET2 | hsa-miR-199b-5p |
| PTEN | hsa-miR-19a-3p |
| PTEN | hsa-miR-19b-3p |
| PTEN | hsa-miR-200a-3p |
| BRD4 | hsa-miR-200a-3p |
| DHX9 | hsa-miR-200b-3p |
| PTEN | hsa-miR-200b-3p |
| DHX9 | hsa-miR-200c-3p |
| PTEN | hsa-miR-200c-3p |
| HMGB1 | hsa-miR-204-5p |
| BRD4 | hsa-miR-204-5p |
| TET2 | hsa-miR-204-5p |
| PTEN | hsa-miR-205-5p |
| TXNIP | hsa-miR-20a-5p |
| PTEN | hsa-miR-20a-5p |
| STAT3 | hsa-miR-20a-5p |
| TET2 | hsa-miR-20a-5p |
| SQSTM1 | hsa-miR-20a-5p |
| TXNIP | hsa-miR-20b-5p |
| PTEN | hsa-miR-20b-5p |
| STAT3 | hsa-miR-20b-5p |
| SQSTM1 | hsa-miR-20b-5p |
| STAT3 | hsa-miR-21-5p |
| HMGB1 | hsa-miR-211-5p |
| BRD4 | hsa-miR-211-5p |
| TET2 | hsa-miR-211-5p |
| PTEN | hsa-miR-216a-5p |
| HMGB1 | hsa-miR-216a-5p |
| HMGB1 | hsa-miR-216b-5p |
| TET2 | hsa-miR-217 |
| HMGB1 | hsa-miR-218-5p |
| PTEN | hsa-miR-22-3p |
| TET2 | hsa-miR-22-3p |
| IRF2 | hsa-miR-221-3p |
| IRF2 | hsa-miR-222-3p |
| PRDM1 | hsa-miR-223-3p |
| NEK7 | hsa-miR-23a-3p |
| PTEN | hsa-miR-23a-3p |
| STK4 | hsa-miR-23a-3p |
| IRF2 | hsa-miR-23a-3p |
| TET2 | hsa-miR-23a-3p |
| NEK7 | hsa-miR-23b-3p |
| PTEN | hsa-miR-23b-3p |
| STK4 | hsa-miR-23b-3p |
| IRF2 | hsa-miR-23b-3p |
| TET2 | hsa-miR-23b-3p |
| IL1A | hsa-miR-24-3p |
| PRDM1 | hsa-miR-24-3p |
| PTEN | hsa-miR-25-3p |
| BAK1 | hsa-miR-25-3p |
| CHMP7 | hsa-miR-25-3p |
| PTEN | hsa-miR-26a-5p |
| TET2 | hsa-miR-26a-5p |
| PTEN | hsa-miR-26b-5p |
| TET2 | hsa-miR-26b-5p |
| ADORA2B | hsa-miR-27a-3p |
| NFE2L2 | hsa-miR-27a-3p |
| CASP8 | hsa-miR-27a-3p |
| ADORA2B | hsa-miR-27b-3p |
| NFE2L2 | hsa-miR-27b-3p |
| CASP8 | hsa-miR-27b-3p |
| NFE2L2 | hsa-miR-28-5p |
| STK4 | hsa-miR-28-5p |
| PTEN | hsa-miR-296-3p |
| PTEN | hsa-miR-29a-3p |
| NLRX1 | hsa-miR-29a-3p |
| BAX | hsa-miR-29a-3p |
| TET2 | hsa-miR-29a-3p |
| PTEN | hsa-miR-29b-3p |
| NLRX1 | hsa-miR-29b-3p |
| BAX | hsa-miR-29b-3p |
| TET2 | hsa-miR-29b-3p |
| PTEN | hsa-miR-29c-3p |
| NLRX1 | hsa-miR-29c-3p |
| BAX | hsa-miR-29c-3p |
| TET2 | hsa-miR-29c-3p |
| TXNIP | hsa-miR-301a-3p |
| PTEN | hsa-miR-301a-3p |
| STAT3 | hsa-miR-301a-3p |
| CHMP3 | hsa-miR-301a-3p |
| TXNIP | hsa-miR-301b-3p |
| PTEN | hsa-miR-301b-3p |
| STAT3 | hsa-miR-301b-3p |
| CHMP3 | hsa-miR-301b-3p |
| TXNIP | hsa-miR-302a-3p |
| VDR | hsa-miR-302a-3p |
| IRF2 | hsa-miR-302a-3p |
| TET2 | hsa-miR-302a-3p |
| SQSTM1 | hsa-miR-302a-3p |
| TXNIP | hsa-miR-302b-3p |
| VDR | hsa-miR-302b-3p |
| IRF2 | hsa-miR-302b-3p |
| TET2 | hsa-miR-302b-3p |
| SQSTM1 | hsa-miR-302b-3p |
| TXNIP | hsa-miR-302c-3p |
| VDR | hsa-miR-302c-3p |
| IRF2 | hsa-miR-302c-3p |
| TET2 | hsa-miR-302c-3p |
| SQSTM1 | hsa-miR-302c-3p |
| TXNIP | hsa-miR-302d-3p |
| VDR | hsa-miR-302d-3p |
| IRF2 | hsa-miR-302d-3p |
| TET2 | hsa-miR-302d-3p |
| SQSTM1 | hsa-miR-302d-3p |
| TXNIP | hsa-miR-302e |
| VDR | hsa-miR-302e |
| IRF2 | hsa-miR-302e |
| TET2 | hsa-miR-302e |
| SQSTM1 | hsa-miR-302e |
| STK4 | hsa-miR-30a-5p |
| CHMP2B | hsa-miR-30a-5p |
| PRDM1 | hsa-miR-30a-5p |
| ABL1 | hsa-miR-30a-5p |
| CHMP2B | hsa-miR-30b-5p |
| PRDM1 | hsa-miR-30b-5p |
| ABL1 | hsa-miR-30b-5p |
| CHMP2B | hsa-miR-30c-5p |
| PRDM1 | hsa-miR-30c-5p |
| ABL1 | hsa-miR-30c-5p |
| STK4 | hsa-miR-30d-5p |
| CHMP2B | hsa-miR-30d-5p |
| PRDM1 | hsa-miR-30d-5p |
| ABL1 | hsa-miR-30d-5p |
| STK4 | hsa-miR-30e-5p |
| CHMP2B | hsa-miR-30e-5p |
| PRDM1 | hsa-miR-30e-5p |
| ABL1 | hsa-miR-30e-5p |
| ELAVL1 | hsa-miR-31-5p |
| PTEN | hsa-miR-32-5p |
| BAK1 | hsa-miR-32-5p |
| CHMP7 | hsa-miR-32-5p |
| PTEN | hsa-miR-320a |
| PTEN | hsa-miR-320b |
| PTEN | hsa-miR-320c |
| PTEN | hsa-miR-320d |
| ELAVL1 | hsa-miR-324-5p |
| CHMP4C | hsa-miR-324-5p |
| BRD4 | hsa-miR-329-3p |
| TFAM | hsa-miR-340-5p |
| NFE2L2 | hsa-miR-340-5p |
| CHMP7 | hsa-miR-340-5p |
| BRD4 | hsa-miR-362-3p |
| PTEN | hsa-miR-363-3p |
| BAK1 | hsa-miR-363-3p |
| CHMP7 | hsa-miR-363-3p |
| PTEN | hsa-miR-365a-3p |
| PRDM1 | hsa-miR-365a-3p |
| PTEN | hsa-miR-367-3p |
| BAK1 | hsa-miR-367-3p |
| CHMP7 | hsa-miR-367-3p |
| TXNIP | hsa-miR-372-3p |
| VDR | hsa-miR-372-3p |
| IRF2 | hsa-miR-372-3p |
| TET2 | hsa-miR-372-3p |
| SQSTM1 | hsa-miR-372-3p |
| TXNIP | hsa-miR-373-3p |
| VDR | hsa-miR-373-3p |
| IRF2 | hsa-miR-373-3p |
| TET2 | hsa-miR-373-3p |
| SQSTM1 | hsa-miR-373-3p |
| PTEN | hsa-miR-374a-5p |
| ADORA2B | hsa-miR-374a-5p |
| CEBPB | hsa-miR-374a-5p |
| TET2 | hsa-miR-374a-5p |
| PTEN | hsa-miR-374b-5p |
| ADORA2B | hsa-miR-374b-5p |
| CEBPB | hsa-miR-374b-5p |
| TET2 | hsa-miR-374b-5p |
| CEBPB | hsa-miR-376a-3p |
| CEBPB | hsa-miR-376b-3p |
| TP53 | hsa-miR-379-5p |
| PTEN | hsa-miR-382-5p |
| PTEN | hsa-miR-410-3p |
| HMGB1 | hsa-miR-410-3p |
| STAT3 | hsa-miR-410-3p |
| CASP3 | hsa-miR-421 |
| TXNIP | hsa-miR-424-5p |
| PTEN | hsa-miR-425-5p |
| DHX9 | hsa-miR-429 |
| PTEN | hsa-miR-429 |
| EEF2K | hsa-miR-448 |
| STK4 | hsa-miR-448 |
| TXNIP | hsa-miR-454-3p |
| PTEN | hsa-miR-454-3p |
| STAT3 | hsa-miR-454-3p |
| CHMP3 | hsa-miR-454-3p |
| PTEN | hsa-miR-485-5p |
| PTEN | hsa-miR-486-5p |
| STK4 | hsa-miR-486-5p |
| TET2 | hsa-miR-490-3p |
| STK4 | hsa-miR-491-5p |
| PTEN | hsa-miR-494-3p |
| PTEN | hsa-miR-495-3p |
| DPP8 | hsa-miR-495-3p |
| ELAVL1 | hsa-miR-495-3p |
| TXNIP | hsa-miR-497-5p |
| HMGB1 | hsa-miR-505-3p |
| TXNIP | hsa-miR-519d-3p |
| PTEN | hsa-miR-519d-3p |
| STAT3 | hsa-miR-519d-3p |
| SQSTM1 | hsa-miR-519d-3p |
| TXNIP | hsa-miR-520a-3p |
| VDR | hsa-miR-520a-3p |
| IRF2 | hsa-miR-520a-3p |
| TET2 | hsa-miR-520a-3p |
| SQSTM1 | hsa-miR-520a-3p |
| TXNIP | hsa-miR-520b |
| VDR | hsa-miR-520b |
| IRF2 | hsa-miR-520b |
| TET2 | hsa-miR-520b |
| SQSTM1 | hsa-miR-520b |
| TXNIP | hsa-miR-520c-3p |
| VDR | hsa-miR-520c-3p |
| IRF2 | hsa-miR-520c-3p |
| TET2 | hsa-miR-520c-3p |
| SQSTM1 | hsa-miR-520c-3p |
| TXNIP | hsa-miR-520d-3p |
| VDR | hsa-miR-520d-3p |
| IRF2 | hsa-miR-520d-3p |
| TET2 | hsa-miR-520d-3p |
| SQSTM1 | hsa-miR-520d-3p |
| TXNIP | hsa-miR-520e |
| VDR | hsa-miR-520e |
| IRF2 | hsa-miR-520e |
| TET2 | hsa-miR-520e |
| SQSTM1 | hsa-miR-520e |
| PTEN | hsa-miR-542-3p |
| PTEN | hsa-miR-543 |
| IL1A | hsa-miR-543 |
| STAT3 | hsa-miR-590-5p |
| PARP1 | hsa-miR-7-5p |
| NR1H2 | hsa-miR-7-5p |
| NFE2L2 | hsa-miR-708-5p |
| STK4 | hsa-miR-708-5p |
| NFE2L2 | hsa-miR-873-5p |
| BRD4 | hsa-miR-9-5p |
| NFKB1 | hsa-miR-9-5p |
| PRDM1 | hsa-miR-9-5p |
| PTEN | hsa-miR-92a-3p |
| BAK1 | hsa-miR-92a-3p |
| CHMP7 | hsa-miR-92a-3p |
| PTEN | hsa-miR-92b-3p |
| BAK1 | hsa-miR-92b-3p |
| CHMP7 | hsa-miR-92b-3p |
| TXNIP | hsa-miR-93-5p |
| PTEN | hsa-miR-93-5p |
| STAT3 | hsa-miR-93-5p |
| SQSTM1 | hsa-miR-93-5p |
| NEK7 | hsa-miR-96-5p |
| EEF2K | hsa-miR-98-5p |
| TP53 | hsa-miR-98-5p |
| CASP3 | hsa-miR-98-5p |

**Table S3** **68 out of top 200 de-lncRNAs have corresponding miRNAs binding.**

| lncRNA | miRNA |
| --- | --- |
| MYH16 | hsa-miR-7 |
| MYH16 | hsa-miR-7ab |
| MYH16 | hsa-miR-133abc |
| MYH16 | hsa-miR-96 |
| MYH16 | hsa-miR-507 |
| MYH16 | hsa-miR-1271 |
| MYH16 | hsa-miR-135ab |
| MYH16 | hsa-miR-135a-5p |
| MYH16 | hsa-miR-138 |
| MYH16 | hsa-miR-138ab |
| MYH16 | hsa-miR-143 |
| MYH16 | hsa-miR-1721 |
| MYH16 | hsa-miR-4770 |
| MYH16 | hsa-miR-145 |
| MYH16 | hsa-miR-153 |
| MYH16 | hsa-miR-15abc |
| MYH16 | hsa-miR-16 |
| MYH16 | hsa-miR-16abc |
| MYH16 | hsa-miR-195 |
| MYH16 | hsa-miR-322 |
| MYH16 | hsa-miR-424 |
| MYH16 | hsa-miR-497 |
| MYH16 | hsa-miR-1907 |
| MYH16 | hsa-miR-17 |
| MYH16 | hsa-miR-17-5p |
| MYH16 | hsa-miR-20ab |
| MYH16 | hsa-miR-20b-5p |
| MYH16 | hsa-miR-93 |
| MYH16 | hsa-miR-106ab |
| MYH16 | hsa-miR-427 |
| MYH16 | hsa-miR-518a-3p |
| MYH16 | hsa-miR-519d |
| MYH16 | hsa-miR-181abcd |
| MYH16 | hsa-miR-4262 |
| MYH16 | hsa-miR-182 |
| MYH16 | hsa-miR-192 |
| MYH16 | hsa-miR-215 |
| MYH16 | hsa-miR-193 |
| MYH16 | hsa-miR-193b |
| MYH16 | hsa-miR-193a-3p |
| MYH16 | hsa-miR-205 |
| MYH16 | hsa-miR-205ab |
| MYH16 | hsa-miR-21 |
| MYH16 | hsa-miR-590-5p |
| MYH16 | hsa-miR-214 |
| MYH16 | hsa-miR-761 |
| MYH16 | hsa-miR-3619-5p |
| MYH16 | hsa-miR-216a |
| MYH16 | hsa-miR-217 |
| MYH16 | hsa-miR-219-5p |
| MYH16 | hsa-miR-508 |
| MYH16 | hsa-miR-508-3p |
| MYH16 | hsa-miR-4782-3p |
| MYH16 | hsa-miR-22 |
| MYH16 | hsa-miR-22-3p |
| MYH16 | hsa-miR-23abc |
| MYH16 | hsa-miR-23b-3p |
| MYH16 | hsa-miR-24 |
| MYH16 | hsa-miR-24ab |
| MYH16 | hsa-miR-24-3p |
| MYH16 | hsa-miR-27abc |
| MYH16 | hsa-miR-27a-3p |
| MYH16 | hsa-miR-29abcd |
| MYH16 | hsa-miR-30abcdef |
| MYH16 | hsa-miR-30abe-5p |
| MYH16 | hsa-miR-384-5p |
| MYH16 | hsa-miR-103a |
| MYH16 | hsa-miR-107 |
| MYH16 | hsa-miR-107ab |
| MYH16 | hsa-miR-338 |
| MYH16 | hsa-miR-338-3p |
| MYH16 | hsa-miR-33a-3p |
| MYH16 | hsa-miR-365 |
| MYH16 | hsa-miR-365-3p |
| MYH16 | hsa-miR-375 |
| MYH16 | hsa-miR-383 |
| MYH16 | hsa-miR-425 |
| MYH16 | hsa-miR-425-5p |
| MYH16 | hsa-miR-489 |
| MYH16 | hsa-miR-10abc |
| MYH16 | hsa-miR-10a-5p |
| MYH16 | hsa-miR-128 |
| MYH16 | hsa-miR-128ab |
| MYH16 | hsa-miR-129-5p |
| MYH16 | hsa-miR-129ab-5p |
| MYH16 | hsa-miR-490-3p |
| H19 | hsa-miR-130ac |
| H19 | hsa-miR-301ab |
| H19 | hsa-miR-301b |
| H19 | hsa-miR-301b-3p |
| H19 | hsa-miR-454 |
| H19 | hsa-miR-721 |
| H19 | hsa-miR-4295 |
| H19 | hsa-miR-3666 |
| H19 | hsa-miR-132 |
| H19 | hsa-miR-212 |
| H19 | hsa-miR-212-3p |
| H19 | hsa-miR-138 |
| H19 | hsa-miR-138ab |
| H19 | hsa-miR-140 |
| H19 | hsa-miR-140-5p |
| H19 | hsa-miR-876-3p |
| H19 | hsa-miR-1244 |
| H19 | hsa-miR-141 |
| H19 | hsa-miR-200a |
| H19 | hsa-miR-148ab-3p |
| H19 | hsa-miR-152 |
| H19 | hsa-miR-17 |
| H19 | hsa-miR-17-5p |
| H19 | hsa-miR-20ab |
| H19 | hsa-miR-20b-5p |
| H19 | hsa-miR-93 |
| H19 | hsa-miR-106ab |
| H19 | hsa-miR-427 |
| H19 | hsa-miR-518a-3p |
| H19 | hsa-miR-519d |
| H19 | hsa-miR-18ab |
| H19 | hsa-miR-4735-3p |
| H19 | hsa-miR-193 |
| H19 | hsa-miR-193b |
| H19 | hsa-miR-193a-3p |
| H19 | hsa-miR-194 |
| H19 | hsa-miR-196abc |
| H19 | hsa-miR-19ab |
| H19 | hsa-miR-1ab |
| H19 | hsa-miR-206 |
| H19 | hsa-miR-613 |
| H19 | hsa-miR-216b |
| H19 | hsa-miR-216b-5p |
| H19 | hsa-miR-22 |
| H19 | hsa-miR-22-3p |
| H19 | hsa-miR-24 |
| H19 | hsa-miR-24ab |
| H19 | hsa-miR-24-3p |
| H19 | hsa-miR-29abcd |
| H19 | hsa-miR-103a |
| H19 | hsa-miR-107 |
| H19 | hsa-miR-107ab |
| H19 | hsa-miR-338 |
| H19 | hsa-miR-338-3p |
| C3P1 | hsa-miR-7 |
| C3P1 | hsa-miR-7ab |
| C3P1 | hsa-miR-133abc |
| C3P1 | hsa-miR-9 |
| C3P1 | hsa-miR-9ab |
| C3P1 | hsa-miR-93 |
| C3P1 | hsa-miR-93a |
| C3P1 | hsa-miR-105 |
| C3P1 | hsa-miR-106a |
| C3P1 | hsa-miR-291a-3p |
| C3P1 | hsa-miR-294 |
| C3P1 | hsa-miR-295 |
| C3P1 | hsa-miR-302abcde |
| C3P1 | hsa-miR-372 |
| C3P1 | hsa-miR-373 |
| C3P1 | hsa-miR-428 |
| C3P1 | hsa-miR-519a |
| C3P1 | hsa-miR-520be |
| C3P1 | hsa-miR-520acd-3p |
| C3P1 | hsa-miR-1378 |
| C3P1 | hsa-miR-1420ac |
| C3P1 | hsa-miR-96 |
| C3P1 | hsa-miR-507 |
| C3P1 | hsa-miR-1271 |
| C3P1 | hsa-miR-135ab |
| C3P1 | hsa-miR-135a-5p |
| C3P1 | hsa-miR-141 |
| C3P1 | hsa-miR-200a |
| C3P1 | hsa-miR-143 |
| C3P1 | hsa-miR-1721 |
| C3P1 | hsa-miR-4770 |
| C3P1 | hsa-miR-145 |
| C3P1 | hsa-miR-146ac |
| C3P1 | hsa-miR-146b-5p |
| C3P1 | hsa-miR-148ab-3p |
| C3P1 | hsa-miR-152 |
| C3P1 | hsa-miR-153 |
| C3P1 | hsa-miR-181abcd |
| C3P1 | hsa-miR-4262 |
| C3P1 | hsa-miR-182 |
| C3P1 | hsa-miR-let-7 |
| C3P1 | hsa-miR-98 |
| C3P1 | hsa-miR-4458 |
| C3P1 | hsa-miR-4500 |
| C3P1 | hsa-miR-193 |
| C3P1 | hsa-miR-193b |
| C3P1 | hsa-miR-193a-3p |
| C3P1 | hsa-miR-1ab |
| C3P1 | hsa-miR-206 |
| C3P1 | hsa-miR-613 |
| C3P1 | hsa-miR-200bc |
| C3P1 | hsa-miR-429 |
| C3P1 | hsa-miR-548a |
| C3P1 | hsa-miR-203 |
| C3P1 | hsa-miR-204 |
| C3P1 | hsa-miR-204b |
| C3P1 | hsa-miR-211 |
| C3P1 | hsa-miR-205 |
| C3P1 | hsa-miR-205ab |
| C3P1 | hsa-miR-217 |
| C3P1 | hsa-miR-22 |
| C3P1 | hsa-miR-22-3p |
| C3P1 | hsa-miR-23abc |
| C3P1 | hsa-miR-23b-3p |
| C3P1 | hsa-miR-24 |
| C3P1 | hsa-miR-24ab |
| C3P1 | hsa-miR-24-3p |
| C3P1 | hsa-miR-26ab |
| C3P1 | hsa-miR-1297 |
| C3P1 | hsa-miR-4465 |
| C3P1 | hsa-miR-27abc |
| C3P1 | hsa-miR-27a-3p |
| C3P1 | hsa-miR-29abcd |
| C3P1 | hsa-miR-31 |
| C3P1 | hsa-miR-103a |
| C3P1 | hsa-miR-107 |
| C3P1 | hsa-miR-107ab |
| C3P1 | hsa-miR-338 |
| C3P1 | hsa-miR-338-3p |
| C3P1 | hsa-miR-425 |
| C3P1 | hsa-miR-425-5p |
| C3P1 | hsa-miR-489 |
| C3P1 | hsa-miR-125a-5p |
| C3P1 | hsa-miR-125b-5p |
| C3P1 | hsa-miR-351 |
| C3P1 | hsa-miR-670 |
| C3P1 | hsa-miR-4319 |
| C3P1 | hsa-miR-128 |
| C3P1 | hsa-miR-128ab |
| WT1-AS | hsa-miR-503 |
| WT1-AS | hsa-miR-133abc |
| WT1-AS | hsa-miR-9 |
| WT1-AS | hsa-miR-9ab |
| WT1-AS | hsa-miR-93 |
| WT1-AS | hsa-miR-93a |
| WT1-AS | hsa-miR-105 |
| WT1-AS | hsa-miR-106a |
| WT1-AS | hsa-miR-291a-3p |
| WT1-AS | hsa-miR-294 |
| WT1-AS | hsa-miR-295 |
| WT1-AS | hsa-miR-302abcde |
| WT1-AS | hsa-miR-372 |
| WT1-AS | hsa-miR-373 |
| WT1-AS | hsa-miR-428 |
| WT1-AS | hsa-miR-519a |
| WT1-AS | hsa-miR-520be |
| WT1-AS | hsa-miR-520acd-3p |
| WT1-AS | hsa-miR-1378 |
| WT1-AS | hsa-miR-1420ac |
| WT1-AS | hsa-miR-96 |
| WT1-AS | hsa-miR-507 |
| WT1-AS | hsa-miR-1271 |
| WT1-AS | hsa-miR-135ab |
| WT1-AS | hsa-miR-135a-5p |
| WT1-AS | hsa-miR-139-5p |
| WT1-AS | hsa-miR-141 |
| WT1-AS | hsa-miR-200a |
| WT1-AS | hsa-miR-145 |
| WT1-AS | hsa-miR-155 |
| WT1-AS | hsa-miR-15abc |
| WT1-AS | hsa-miR-16 |
| WT1-AS | hsa-miR-16abc |
| WT1-AS | hsa-miR-195 |
| WT1-AS | hsa-miR-322 |
| WT1-AS | hsa-miR-424 |
| WT1-AS | hsa-miR-497 |
| WT1-AS | hsa-miR-1907 |
| WT1-AS | hsa-miR-17 |
| WT1-AS | hsa-miR-17-5p |
| WT1-AS | hsa-miR-20ab |
| WT1-AS | hsa-miR-20b-5p |
| WT1-AS | hsa-miR-106ab |
| WT1-AS | hsa-miR-427 |
| WT1-AS | hsa-miR-518a-3p |
| WT1-AS | hsa-miR-519d |
| WT1-AS | hsa-miR-181abcd |
| WT1-AS | hsa-miR-4262 |
| WT1-AS | hsa-miR-182 |
| WT1-AS | hsa-miR-let-7 |
| WT1-AS | hsa-miR-98 |
| WT1-AS | hsa-miR-4458 |
| WT1-AS | hsa-miR-4500 |
| WT1-AS | hsa-miR-18ab |
| WT1-AS | hsa-miR-4735-3p |
| WT1-AS | hsa-miR-193 |
| WT1-AS | hsa-miR-193b |
| WT1-AS | hsa-miR-193a-3p |
| WT1-AS | hsa-miR-199ab-5p |
| WT1-AS | hsa-miR-1ab |
| WT1-AS | hsa-miR-206 |
| WT1-AS | hsa-miR-613 |
| WT1-AS | hsa-miR-200bc |
| WT1-AS | hsa-miR-429 |
| WT1-AS | hsa-miR-548a |
| WT1-AS | hsa-miR-203 |
| WT1-AS | hsa-miR-208ab |
| WT1-AS | hsa-miR-208ab-3p |
| WT1-AS | hsa-miR-216b |
| WT1-AS | hsa-miR-216b-5p |
| WT1-AS | hsa-miR-217 |
| WT1-AS | hsa-miR-218 |
| WT1-AS | hsa-miR-218a |
| WT1-AS | hsa-miR-22 |
| WT1-AS | hsa-miR-22-3p |
| WT1-AS | hsa-miR-221 |
| WT1-AS | hsa-miR-222 |
| WT1-AS | hsa-miR-222ab |
| WT1-AS | hsa-miR-1928 |
| WT1-AS | hsa-miR-223 |
| WT1-AS | hsa-miR-23abc |
| WT1-AS | hsa-miR-23b-3p |
| WT1-AS | hsa-miR-24 |
| WT1-AS | hsa-miR-24ab |
| WT1-AS | hsa-miR-24-3p |
| WT1-AS | hsa-miR-25 |
| WT1-AS | hsa-miR-32 |
| WT1-AS | hsa-miR-92abc |
| WT1-AS | hsa-miR-363 |
| WT1-AS | hsa-miR-363-3p |
| WT1-AS | hsa-miR-367 |
| WT1-AS | hsa-miR-27abc |
| WT1-AS | hsa-miR-27a-3p |
| WT1-AS | hsa-miR-30abcdef |
| WT1-AS | hsa-miR-30abe-5p |
| WT1-AS | hsa-miR-384-5p |
| WT1-AS | hsa-miR-31 |
| WT1-AS | hsa-miR-34ac |
| WT1-AS | hsa-miR-34bc-5p |
| WT1-AS | hsa-miR-449abc |
| WT1-AS | hsa-miR-449c-5p |
| WT1-AS | hsa-miR-375 |
| WT1-AS | hsa-miR-383 |
| WT1-AS | hsa-miR-125a-5p |
| WT1-AS | hsa-miR-125b-5p |
| WT1-AS | hsa-miR-351 |
| WT1-AS | hsa-miR-670 |
| WT1-AS | hsa-miR-4319 |
| WT1-AS | hsa-miR-129-5p |
| WT1-AS | hsa-miR-129ab-5p |
| LINC00518 | hsa-miR-133abc |
| LINC00518 | hsa-miR-135ab |
| LINC00518 | hsa-miR-135a-5p |
| LINC00518 | hsa-miR-138 |
| LINC00518 | hsa-miR-138ab |
| LINC00518 | hsa-miR-141 |
| LINC00518 | hsa-miR-200a |
| LINC00518 | hsa-miR-143 |
| LINC00518 | hsa-miR-1721 |
| LINC00518 | hsa-miR-4770 |
| LINC00518 | hsa-miR-145 |
| LINC00518 | hsa-miR-150 |
| LINC00518 | hsa-miR-5127 |
| LINC00518 | hsa-miR-199ab-5p |
| LINC00518 | hsa-miR-19ab |
| LINC00518 | hsa-miR-1ab |
| LINC00518 | hsa-miR-206 |
| LINC00518 | hsa-miR-613 |
| LINC00518 | hsa-miR-203 |
| LINC00518 | hsa-miR-204 |
| LINC00518 | hsa-miR-204b |
| LINC00518 | hsa-miR-211 |
| LINC00518 | hsa-miR-216a |
| LINC00518 | hsa-miR-216b |
| LINC00518 | hsa-miR-216b-5p |
| LINC00518 | hsa-miR-24 |
| LINC00518 | hsa-miR-24ab |
| LINC00518 | hsa-miR-24-3p |
| LINC00518 | hsa-miR-27abc |
| LINC00518 | hsa-miR-27a-3p |
| LINC00518 | hsa-miR-29abcd |
| LINC00518 | hsa-miR-33ab |
| LINC00518 | hsa-miR-33-5p |
| LINC00518 | hsa-miR-375 |
| LINC00518 | hsa-miR-125a-5p |
| LINC00518 | hsa-miR-125b-5p |
| LINC00518 | hsa-miR-351 |
| LINC00518 | hsa-miR-670 |
| LINC00518 | hsa-miR-4319 |
| LINC00518 | hsa-miR-128 |
| LINC00518 | hsa-miR-128ab |
| LINC00518 | hsa-miR-129-5p |
| LINC00518 | hsa-miR-129ab-5p |
| ANKRD20A19P | hsa-miR-503 |
| ANKRD20A19P | hsa-miR-9 |
| ANKRD20A19P | hsa-miR-9ab |
| ANKRD20A19P | hsa-miR-96 |
| ANKRD20A19P | hsa-miR-507 |
| ANKRD20A19P | hsa-miR-1271 |
| ANKRD20A19P | hsa-miR-137 |
| ANKRD20A19P | hsa-miR-137ab |
| ANKRD20A19P | hsa-miR-139-5p |
| ANKRD20A19P | hsa-miR-143 |
| ANKRD20A19P | hsa-miR-1721 |
| ANKRD20A19P | hsa-miR-4770 |
| ANKRD20A19P | hsa-miR-148ab-3p |
| ANKRD20A19P | hsa-miR-152 |
| ANKRD20A19P | hsa-miR-15abc |
| ANKRD20A19P | hsa-miR-16 |
| ANKRD20A19P | hsa-miR-16abc |
| ANKRD20A19P | hsa-miR-195 |
| ANKRD20A19P | hsa-miR-322 |
| ANKRD20A19P | hsa-miR-424 |
| ANKRD20A19P | hsa-miR-497 |
| ANKRD20A19P | hsa-miR-1907 |
| ANKRD20A19P | hsa-miR-17 |
| ANKRD20A19P | hsa-miR-17-5p |
| ANKRD20A19P | hsa-miR-20ab |
| ANKRD20A19P | hsa-miR-20b-5p |
| ANKRD20A19P | hsa-miR-93 |
| ANKRD20A19P | hsa-miR-106ab |
| ANKRD20A19P | hsa-miR-427 |
| ANKRD20A19P | hsa-miR-518a-3p |
| ANKRD20A19P | hsa-miR-519d |
| ANKRD20A19P | hsa-miR-182 |
| ANKRD20A19P | hsa-miR-183 |
| ANKRD20A19P | hsa-miR-192 |
| ANKRD20A19P | hsa-miR-215 |
| ANKRD20A19P | hsa-miR-199ab-5p |
| ANKRD20A19P | hsa-miR-204 |
| ANKRD20A19P | hsa-miR-204b |
| ANKRD20A19P | hsa-miR-211 |
| ANKRD20A19P | hsa-miR-205 |
| ANKRD20A19P | hsa-miR-205ab |
| ANKRD20A19P | hsa-miR-214 |
| ANKRD20A19P | hsa-miR-761 |
| ANKRD20A19P | hsa-miR-3619-5p |
| ANKRD20A19P | hsa-miR-217 |
| ANKRD20A19P | hsa-miR-223 |
| ANKRD20A19P | hsa-miR-24 |
| ANKRD20A19P | hsa-miR-24ab |
| ANKRD20A19P | hsa-miR-24-3p |
| ANKRD20A19P | hsa-miR-124 |
| ANKRD20A19P | hsa-miR-124ab |
| ANKRD20A19P | hsa-miR-506 |
| ANKRD20A19P | hsa-miR-338 |
| ANKRD20A19P | hsa-miR-338-3p |
| ANKRD20A19P | hsa-miR-375 |
| ANKRD20A19P | hsa-miR-125a-5p |
| ANKRD20A19P | hsa-miR-125b-5p |
| ANKRD20A19P | hsa-miR-351 |
| ANKRD20A19P | hsa-miR-670 |
| ANKRD20A19P | hsa-miR-4319 |
| ANKRD20A19P | hsa-miR-129-5p |
| ANKRD20A19P | hsa-miR-129ab-5p |
| TERC | hsa-miR-34ac |
| TERC | hsa-miR-34bc-5p |
| TERC | hsa-miR-449abc |
| TERC | hsa-miR-449c-5p |
| TERC | hsa-miR-129-5p |
| TERC | hsa-miR-129ab-5p |
| PPP1R14BP2 | hsa-miR-143 |
| PPP1R14BP2 | hsa-miR-1721 |
| PPP1R14BP2 | hsa-miR-4770 |
| PPP1R14BP2 | hsa-miR-145 |
| PPP1R14BP2 | hsa-miR-194 |
| PPP1R14BP2 | hsa-miR-214 |
| PPP1R14BP2 | hsa-miR-761 |
| PPP1R14BP2 | hsa-miR-3619-5p |
| PPP1R14BP2 | hsa-miR-338 |
| PPP1R14BP2 | hsa-miR-338-3p |
| PPP1R14BP2 | hsa-miR-34ac |
| PPP1R14BP2 | hsa-miR-34bc-5p |
| PPP1R14BP2 | hsa-miR-449abc |
| PPP1R14BP2 | hsa-miR-449c-5p |
| ARF1P2 | hsa-miR-144 |
| ARF1P2 | hsa-miR-148ab-3p |
| ARF1P2 | hsa-miR-152 |
| ARF1P2 | hsa-miR-182 |
| ARF1P2 | hsa-miR-223 |
| ARF1P2 | hsa-miR-103a |
| ARF1P2 | hsa-miR-107 |
| ARF1P2 | hsa-miR-107ab |
| ARF1P2 | hsa-miR-338 |
| ARF1P2 | hsa-miR-338-3p |
| ARF1P2 | hsa-miR-128 |
| ARF1P2 | hsa-miR-128ab |
| UCA1 | hsa-miR-96 |
| UCA1 | hsa-miR-507 |
| UCA1 | hsa-miR-1271 |
| UCA1 | hsa-miR-135ab |
| UCA1 | hsa-miR-135a-5p |
| UCA1 | hsa-miR-138 |
| UCA1 | hsa-miR-138ab |
| UCA1 | hsa-miR-143 |
| UCA1 | hsa-miR-1721 |
| UCA1 | hsa-miR-4770 |
| UCA1 | hsa-miR-182 |
| UCA1 | hsa-miR-184 |
| UCA1 | hsa-miR-18ab |
| UCA1 | hsa-miR-4735-3p |
| UCA1 | hsa-miR-190 |
| UCA1 | hsa-miR-190ab |
| UCA1 | hsa-miR-193 |
| UCA1 | hsa-miR-193b |
| UCA1 | hsa-miR-193a-3p |
| UCA1 | hsa-miR-1ab |
| UCA1 | hsa-miR-206 |
| UCA1 | hsa-miR-613 |
| UCA1 | hsa-miR-203 |
| UCA1 | hsa-miR-214 |
| UCA1 | hsa-miR-761 |
| UCA1 | hsa-miR-3619-5p |
| UCA1 | hsa-miR-122 |
| UCA1 | hsa-miR-122a |
| UCA1 | hsa-miR-1352 |
| UCA1 | hsa-miR-23abc |
| UCA1 | hsa-miR-23b-3p |
| UCA1 | hsa-miR-26ab |
| UCA1 | hsa-miR-1297 |
| UCA1 | hsa-miR-4465 |
| UCA1 | hsa-miR-103a |
| UCA1 | hsa-miR-107 |
| UCA1 | hsa-miR-107ab |
| UCA1 | hsa-miR-124 |
| UCA1 | hsa-miR-124ab |
| UCA1 | hsa-miR-506 |
| UCA1 | hsa-miR-383 |
| UCA1 | hsa-miR-455-5p |
| UCA1 | hsa-miR-129-5p |
| UCA1 | hsa-miR-129ab-5p |
| RPS19P1 | hsa-miR-133abc |
| RPS19P1 | hsa-miR-218 |
| RPS19P1 | hsa-miR-218a |
| RPS19P1 | hsa-miR-22 |
| RPS19P1 | hsa-miR-22-3p |
| RPS19P1 | hsa-miR-223 |
| RPS19P1 | hsa-miR-125a-5p |
| RPS19P1 | hsa-miR-125b-5p |
| RPS19P1 | hsa-miR-351 |
| RPS19P1 | hsa-miR-670 |
| RPS19P1 | hsa-miR-4319 |
| MIR17HG | hsa-miR-203 |
| MIR17HG | hsa-miR-23abc |
| MIR17HG | hsa-miR-23b-3p |
| MIR17HG | hsa-miR-425 |
| MIR17HG | hsa-miR-425-5p |
| MIR17HG | hsa-miR-489 |
| MIR17HG | hsa-miR-455-5p |
| CCT7P1 | hsa-miR-551a |
| CCT7P1 | hsa-miR-96 |
| CCT7P1 | hsa-miR-507 |
| CCT7P1 | hsa-miR-1271 |
| CCT7P1 | hsa-miR-143 |
| CCT7P1 | hsa-miR-1721 |
| CCT7P1 | hsa-miR-4770 |
| CCT7P1 | hsa-miR-146ac |
| CCT7P1 | hsa-miR-146b-5p |
| CCT7P1 | hsa-miR-181abcd |
| CCT7P1 | hsa-miR-4262 |
| CCT7P1 | hsa-miR-182 |
| CCT7P1 | hsa-miR-18ab |
| CCT7P1 | hsa-miR-4735-3p |
| CCT7P1 | hsa-miR-199ab-5p |
| CCT7P1 | hsa-miR-1ab |
| CCT7P1 | hsa-miR-206 |
| CCT7P1 | hsa-miR-613 |
| CCT7P1 | hsa-miR-205 |
| CCT7P1 | hsa-miR-205ab |
| CCT7P1 | hsa-miR-216b |
| CCT7P1 | hsa-miR-216b-5p |
| CCT7P1 | hsa-miR-217 |
| CCT7P1 | hsa-miR-22 |
| CCT7P1 | hsa-miR-22-3p |
| CCT7P1 | hsa-miR-27abc |
| CCT7P1 | hsa-miR-27a-3p |
| CCT7P1 | hsa-miR-30abcdef |
| CCT7P1 | hsa-miR-30abe-5p |
| CCT7P1 | hsa-miR-384-5p |
| CCT7P1 | hsa-miR-103a |
| CCT7P1 | hsa-miR-107 |
| CCT7P1 | hsa-miR-107ab |
| CCT7P1 | hsa-miR-338 |
| CCT7P1 | hsa-miR-338-3p |
| CCT7P1 | hsa-miR-33ab |
| CCT7P1 | hsa-miR-33-5p |
| CCT7P1 | hsa-miR-128 |
| CCT7P1 | hsa-miR-128ab |
| BRD7P4 | hsa-miR-130ac |
| BRD7P4 | hsa-miR-301ab |
| BRD7P4 | hsa-miR-301b |
| BRD7P4 | hsa-miR-301b-3p |
| BRD7P4 | hsa-miR-454 |
| BRD7P4 | hsa-miR-721 |
| BRD7P4 | hsa-miR-4295 |
| BRD7P4 | hsa-miR-3666 |
| BRD7P4 | hsa-miR-7 |
| BRD7P4 | hsa-miR-7ab |
| BRD7P4 | hsa-miR-96 |
| BRD7P4 | hsa-miR-507 |
| BRD7P4 | hsa-miR-1271 |
| BRD7P4 | hsa-miR-137 |
| BRD7P4 | hsa-miR-137ab |
| BRD7P4 | hsa-miR-141 |
| BRD7P4 | hsa-miR-200a |
| BRD7P4 | hsa-miR-143 |
| BRD7P4 | hsa-miR-1721 |
| BRD7P4 | hsa-miR-4770 |
| BRD7P4 | hsa-miR-145 |
| BRD7P4 | hsa-miR-153 |
| BRD7P4 | hsa-miR-181abcd |
| BRD7P4 | hsa-miR-4262 |
| BRD7P4 | hsa-miR-182 |
| BRD7P4 | hsa-miR-19ab |
| BRD7P4 | hsa-miR-1ab |
| BRD7P4 | hsa-miR-206 |
| BRD7P4 | hsa-miR-613 |
| BRD7P4 | hsa-miR-205 |
| BRD7P4 | hsa-miR-205ab |
| BRD7P4 | hsa-miR-216a |
| BRD7P4 | hsa-miR-216b |
| BRD7P4 | hsa-miR-216b-5p |
| BRD7P4 | hsa-miR-218 |
| BRD7P4 | hsa-miR-218a |
| BRD7P4 | hsa-miR-122 |
| BRD7P4 | hsa-miR-122a |
| BRD7P4 | hsa-miR-1352 |
| BRD7P4 | hsa-miR-33a-3p |
| BRD7P4 | hsa-miR-365 |
| BRD7P4 | hsa-miR-365-3p |
| BRD7P4 | hsa-miR-34ac |
| BRD7P4 | hsa-miR-34bc-5p |
| BRD7P4 | hsa-miR-449abc |
| BRD7P4 | hsa-miR-449c-5p |
| BRD7P4 | hsa-miR-375 |
| BRD7P4 | hsa-miR-125a-5p |
| BRD7P4 | hsa-miR-125b-5p |
| BRD7P4 | hsa-miR-351 |
| BRD7P4 | hsa-miR-670 |
| BRD7P4 | hsa-miR-4319 |
| BRD7P4 | hsa-miR-455-5p |
| HIGD1AP16 | hsa-miR-132 |
| HIGD1AP16 | hsa-miR-212 |
| HIGD1AP16 | hsa-miR-212-3p |
| HIGD1AP16 | hsa-miR-383 |
| HIGD1AP16 | hsa-miR-125a-5p |
| HIGD1AP16 | hsa-miR-125b-5p |
| HIGD1AP16 | hsa-miR-351 |
| HIGD1AP16 | hsa-miR-670 |
| HIGD1AP16 | hsa-miR-4319 |
| SMARCE1P1 | hsa-miR-133abc |
| SMARCE1P1 | hsa-miR-96 |
| SMARCE1P1 | hsa-miR-507 |
| SMARCE1P1 | hsa-miR-1271 |
| SMARCE1P1 | hsa-miR-143 |
| SMARCE1P1 | hsa-miR-1721 |
| SMARCE1P1 | hsa-miR-4770 |
| SMARCE1P1 | hsa-miR-150 |
| SMARCE1P1 | hsa-miR-5127 |
| SMARCE1P1 | hsa-miR-199ab-5p |
| SMARCE1P1 | hsa-miR-19ab |
| SMARCE1P1 | hsa-miR-1ab |
| SMARCE1P1 | hsa-miR-206 |
| SMARCE1P1 | hsa-miR-613 |
| SMARCE1P1 | hsa-miR-204 |
| SMARCE1P1 | hsa-miR-204b |
| SMARCE1P1 | hsa-miR-211 |
| SMARCE1P1 | hsa-miR-205 |
| SMARCE1P1 | hsa-miR-205ab |
| SMARCE1P1 | hsa-miR-21 |
| SMARCE1P1 | hsa-miR-590-5p |
| SMARCE1P1 | hsa-miR-214 |
| SMARCE1P1 | hsa-miR-761 |
| SMARCE1P1 | hsa-miR-3619-5p |
| SMARCE1P1 | hsa-miR-216a |
| SMARCE1P1 | hsa-miR-218 |
| SMARCE1P1 | hsa-miR-218a |
| SMARCE1P1 | hsa-miR-122 |
| SMARCE1P1 | hsa-miR-122a |
| SMARCE1P1 | hsa-miR-1352 |
| SMARCE1P1 | hsa-miR-33ab |
| SMARCE1P1 | hsa-miR-33-5p |
| SMARCE1P1 | hsa-miR-129-5p |
| SMARCE1P1 | hsa-miR-129ab-5p |
| TDGF1P3 | hsa-miR-503 |
| TDGF1P3 | hsa-miR-7 |
| TDGF1P3 | hsa-miR-7ab |
| TDGF1P3 | hsa-miR-214 |
| TDGF1P3 | hsa-miR-761 |
| TDGF1P3 | hsa-miR-3619-5p |
| TDGF1P3 | hsa-miR-27abc |
| TDGF1P3 | hsa-miR-27a-3p |
| TDGF1P3 | hsa-miR-338 |
| TDGF1P3 | hsa-miR-338-3p |
| TDGF1P3 | hsa-miR-128 |
| TDGF1P3 | hsa-miR-128ab |
| SP3P | hsa-miR-503 |
| SP3P | hsa-miR-132 |
| SP3P | hsa-miR-212 |
| SP3P | hsa-miR-212-3p |
| SP3P | hsa-miR-133abc |
| SP3P | hsa-miR-96 |
| SP3P | hsa-miR-507 |
| SP3P | hsa-miR-1271 |
| SP3P | hsa-miR-138 |
| SP3P | hsa-miR-138ab |
| SP3P | hsa-miR-139-5p |
| SP3P | hsa-miR-143 |
| SP3P | hsa-miR-1721 |
| SP3P | hsa-miR-4770 |
| SP3P | hsa-miR-145 |
| SP3P | hsa-miR-146ac |
| SP3P | hsa-miR-146b-5p |
| SP3P | hsa-miR-148ab-3p |
| SP3P | hsa-miR-152 |
| SP3P | hsa-miR-150 |
| SP3P | hsa-miR-5127 |
| SP3P | hsa-miR-155 |
| SP3P | hsa-miR-15abc |
| SP3P | hsa-miR-16 |
| SP3P | hsa-miR-16abc |
| SP3P | hsa-miR-195 |
| SP3P | hsa-miR-322 |
| SP3P | hsa-miR-424 |
| SP3P | hsa-miR-497 |
| SP3P | hsa-miR-1907 |
| SP3P | hsa-miR-219-5p |
| SP3P | hsa-miR-508 |
| SP3P | hsa-miR-508-3p |
| SP3P | hsa-miR-4782-3p |
| SP3P | hsa-miR-31 |
| SP3P | hsa-miR-338 |
| SP3P | hsa-miR-338-3p |
| SP3P | hsa-miR-33a-3p |
| SP3P | hsa-miR-365 |
| SP3P | hsa-miR-365-3p |
| SP3P | hsa-miR-375 |
| SP3P | hsa-miR-125a-5p |
| SP3P | hsa-miR-125b-5p |
| SP3P | hsa-miR-351 |
| SP3P | hsa-miR-670 |
| SP3P | hsa-miR-4319 |
| SP3P | hsa-miR-451 |
| SP3P | hsa-miR-490-3p |
| PHF2P2 | hsa-miR-9 |
| PHF2P2 | hsa-miR-9ab |
| PHF2P2 | hsa-miR-93 |
| PHF2P2 | hsa-miR-93a |
| PHF2P2 | hsa-miR-105 |
| PHF2P2 | hsa-miR-106a |
| PHF2P2 | hsa-miR-291a-3p |
| PHF2P2 | hsa-miR-294 |
| PHF2P2 | hsa-miR-295 |
| PHF2P2 | hsa-miR-302abcde |
| PHF2P2 | hsa-miR-372 |
| PHF2P2 | hsa-miR-373 |
| PHF2P2 | hsa-miR-428 |
| PHF2P2 | hsa-miR-519a |
| PHF2P2 | hsa-miR-520be |
| PHF2P2 | hsa-miR-520acd-3p |
| PHF2P2 | hsa-miR-1378 |
| PHF2P2 | hsa-miR-1420ac |
| PHF2P2 | hsa-miR-135ab |
| PHF2P2 | hsa-miR-135a-5p |
| PHF2P2 | hsa-miR-141 |
| PHF2P2 | hsa-miR-200a |
| PHF2P2 | hsa-miR-144 |
| PHF2P2 | hsa-miR-148ab-3p |
| PHF2P2 | hsa-miR-152 |
| PHF2P2 | hsa-miR-150 |
| PHF2P2 | hsa-miR-5127 |
| PHF2P2 | hsa-miR-205 |
| PHF2P2 | hsa-miR-205ab |
| PHF2P2 | hsa-miR-214 |
| PHF2P2 | hsa-miR-761 |
| PHF2P2 | hsa-miR-3619-5p |
| PHF2P2 | hsa-miR-23abc |
| PHF2P2 | hsa-miR-23b-3p |
| PHF2P2 | hsa-miR-27abc |
| PHF2P2 | hsa-miR-27a-3p |
| PHF2P2 | hsa-miR-29abcd |
| PHF2P2 | hsa-miR-103a |
| PHF2P2 | hsa-miR-107 |
| PHF2P2 | hsa-miR-107ab |
| PHF2P2 | hsa-miR-338 |
| PHF2P2 | hsa-miR-338-3p |
| PHF2P2 | hsa-miR-34ac |
| PHF2P2 | hsa-miR-34bc-5p |
| PHF2P2 | hsa-miR-449abc |
| PHF2P2 | hsa-miR-449c-5p |
| PHF2P2 | hsa-miR-375 |
| PHF2P2 | hsa-miR-125a-5p |
| PHF2P2 | hsa-miR-125b-5p |
| PHF2P2 | hsa-miR-351 |
| PHF2P2 | hsa-miR-670 |
| PHF2P2 | hsa-miR-4319 |
| PHF2P2 | hsa-miR-128 |
| PHF2P2 | hsa-miR-128ab |
| PHF2P2 | hsa-miR-129-5p |
| PHF2P2 | hsa-miR-129ab-5p |
| PHF2P2 | hsa-miR-490-3p |
| NPY6R | hsa-miR-132 |
| NPY6R | hsa-miR-212 |
| NPY6R | hsa-miR-212-3p |
| NPY6R | hsa-miR-138 |
| NPY6R | hsa-miR-138ab |
| NPY6R | hsa-miR-139-5p |
| NPY6R | hsa-miR-144 |
| NPY6R | hsa-miR-146ac |
| NPY6R | hsa-miR-146b-5p |
| NPY6R | hsa-miR-148ab-3p |
| NPY6R | hsa-miR-152 |
| NPY6R | hsa-miR-150 |
| NPY6R | hsa-miR-5127 |
| NPY6R | hsa-miR-153 |
| NPY6R | hsa-miR-182 |
| NPY6R | hsa-miR-199ab-5p |
| NPY6R | hsa-miR-200bc |
| NPY6R | hsa-miR-429 |
| NPY6R | hsa-miR-548a |
| NPY6R | hsa-miR-208ab |
| NPY6R | hsa-miR-208ab-3p |
| NPY6R | hsa-miR-21 |
| NPY6R | hsa-miR-590-5p |
| NPY6R | hsa-miR-214 |
| NPY6R | hsa-miR-761 |
| NPY6R | hsa-miR-3619-5p |
| NPY6R | hsa-miR-217 |
| NPY6R | hsa-miR-218 |
| NPY6R | hsa-miR-218a |
| NPY6R | hsa-miR-122 |
| NPY6R | hsa-miR-122a |
| NPY6R | hsa-miR-1352 |
| NPY6R | hsa-miR-23abc |
| NPY6R | hsa-miR-23b-3p |
| NPY6R | hsa-miR-24 |
| NPY6R | hsa-miR-24ab |
| NPY6R | hsa-miR-24-3p |
| NPY6R | hsa-miR-25 |
| NPY6R | hsa-miR-32 |
| NPY6R | hsa-miR-92abc |
| NPY6R | hsa-miR-363 |
| NPY6R | hsa-miR-363-3p |
| NPY6R | hsa-miR-367 |
| NPY6R | hsa-miR-26ab |
| NPY6R | hsa-miR-1297 |
| NPY6R | hsa-miR-4465 |
| NPY6R | hsa-miR-29abcd |
| NPY6R | hsa-miR-103a |
| NPY6R | hsa-miR-107 |
| NPY6R | hsa-miR-107ab |
| NPY6R | hsa-miR-124 |
| NPY6R | hsa-miR-124ab |
| NPY6R | hsa-miR-506 |
| NPY6R | hsa-miR-33ab |
| NPY6R | hsa-miR-33-5p |
| NPY6R | hsa-miR-375 |
| NPY6R | hsa-miR-425 |
| NPY6R | hsa-miR-425-5p |
| NPY6R | hsa-miR-489 |
| NPY6R | hsa-miR-125a-5p |
| NPY6R | hsa-miR-125b-5p |
| NPY6R | hsa-miR-351 |
| NPY6R | hsa-miR-670 |
| NPY6R | hsa-miR-4319 |
| NPY6R | hsa-miR-10abc |
| NPY6R | hsa-miR-10a-5p |
| NPY6R | hsa-miR-129-5p |
| NPY6R | hsa-miR-129ab-5p |
| NPY6R | hsa-miR-499-5p |
| MRPL23-AS1 | hsa-miR-503 |
| MRPL23-AS1 | hsa-miR-551a |
| MRPL23-AS1 | hsa-miR-138 |
| MRPL23-AS1 | hsa-miR-138ab |
| MRPL23-AS1 | hsa-miR-143 |
| MRPL23-AS1 | hsa-miR-1721 |
| MRPL23-AS1 | hsa-miR-4770 |
| MRPL23-AS1 | hsa-miR-145 |
| MRPL23-AS1 | hsa-miR-150 |
| MRPL23-AS1 | hsa-miR-5127 |
| MRPL23-AS1 | hsa-miR-193 |
| MRPL23-AS1 | hsa-miR-193b |
| MRPL23-AS1 | hsa-miR-193a-3p |
| MRPL23-AS1 | hsa-miR-199ab-5p |
| MRPL23-AS1 | hsa-miR-22 |
| MRPL23-AS1 | hsa-miR-22-3p |
| MRPL23-AS1 | hsa-miR-24 |
| MRPL23-AS1 | hsa-miR-24ab |
| MRPL23-AS1 | hsa-miR-24-3p |
| MRPL23-AS1 | hsa-miR-33a-3p |
| MRPL23-AS1 | hsa-miR-365 |
| MRPL23-AS1 | hsa-miR-365-3p |
| MRPL23-AS1 | hsa-miR-34ac |
| MRPL23-AS1 | hsa-miR-34bc-5p |
| MRPL23-AS1 | hsa-miR-449abc |
| MRPL23-AS1 | hsa-miR-449c-5p |
| MRPL23-AS1 | hsa-miR-10abc |
| MRPL23-AS1 | hsa-miR-10a-5p |
| MRPL23-AS1 | hsa-miR-128 |
| MRPL23-AS1 | hsa-miR-128ab |
| MRPL23-AS1 | hsa-miR-490-3p |
| SCDP1 | hsa-miR-182 |
| SCDP1 | hsa-miR-1ab |
| SCDP1 | hsa-miR-206 |
| SCDP1 | hsa-miR-613 |
| SCDP1 | hsa-miR-29abcd |
| SCDP1 | hsa-miR-103a |
| SCDP1 | hsa-miR-107 |
| SCDP1 | hsa-miR-107ab |
| SCDP1 | hsa-miR-383 |
| SCDP1 | hsa-miR-455-5p |
| SLAMF6P1 | hsa-miR-205 |
| SLAMF6P1 | hsa-miR-205ab |
| STEAP2-AS1 | hsa-miR-7 |
| STEAP2-AS1 | hsa-miR-7ab |
| STEAP2-AS1 | hsa-miR-137 |
| STEAP2-AS1 | hsa-miR-137ab |
| STEAP2-AS1 | hsa-miR-138 |
| STEAP2-AS1 | hsa-miR-138ab |
| STEAP2-AS1 | hsa-miR-143 |
| STEAP2-AS1 | hsa-miR-1721 |
| STEAP2-AS1 | hsa-miR-4770 |
| STEAP2-AS1 | hsa-miR-146ac |
| STEAP2-AS1 | hsa-miR-146b-5p |
| STEAP2-AS1 | hsa-miR-148ab-3p |
| STEAP2-AS1 | hsa-miR-152 |
| STEAP2-AS1 | hsa-miR-155 |
| STEAP2-AS1 | hsa-miR-15abc |
| STEAP2-AS1 | hsa-miR-16 |
| STEAP2-AS1 | hsa-miR-16abc |
| STEAP2-AS1 | hsa-miR-195 |
| STEAP2-AS1 | hsa-miR-322 |
| STEAP2-AS1 | hsa-miR-424 |
| STEAP2-AS1 | hsa-miR-497 |
| STEAP2-AS1 | hsa-miR-1907 |
| STEAP2-AS1 | hsa-miR-24 |
| STEAP2-AS1 | hsa-miR-24ab |
| STEAP2-AS1 | hsa-miR-24-3p |
| STEAP2-AS1 | hsa-miR-103a |
| STEAP2-AS1 | hsa-miR-107 |
| STEAP2-AS1 | hsa-miR-107ab |
| STEAP2-AS1 | hsa-miR-338 |
| STEAP2-AS1 | hsa-miR-338-3p |
| STEAP2-AS1 | hsa-miR-33a-3p |
| STEAP2-AS1 | hsa-miR-365 |
| STEAP2-AS1 | hsa-miR-365-3p |
| STEAP2-AS1 | hsa-miR-375 |
| STEAP2-AS1 | hsa-miR-499-5p |
| LINC00355 | hsa-miR-503 |
| LINC00355 | hsa-miR-135ab |
| LINC00355 | hsa-miR-135a-5p |
| LINC00355 | hsa-miR-140 |
| LINC00355 | hsa-miR-140-5p |
| LINC00355 | hsa-miR-876-3p |
| LINC00355 | hsa-miR-1244 |
| LINC00355 | hsa-miR-141 |
| LINC00355 | hsa-miR-200a |
| LINC00355 | hsa-miR-142-3p |
| LINC00355 | hsa-miR-150 |
| LINC00355 | hsa-miR-5127 |
| LINC00355 | hsa-miR-153 |
| LINC00355 | hsa-miR-15abc |
| LINC00355 | hsa-miR-16 |
| LINC00355 | hsa-miR-16abc |
| LINC00355 | hsa-miR-195 |
| LINC00355 | hsa-miR-322 |
| LINC00355 | hsa-miR-424 |
| LINC00355 | hsa-miR-497 |
| LINC00355 | hsa-miR-1907 |
| LINC00355 | hsa-miR-181abcd |
| LINC00355 | hsa-miR-4262 |
| LINC00355 | hsa-miR-let-7 |
| LINC00355 | hsa-miR-98 |
| LINC00355 | hsa-miR-4458 |
| LINC00355 | hsa-miR-4500 |
| LINC00355 | hsa-miR-214 |
| LINC00355 | hsa-miR-761 |
| LINC00355 | hsa-miR-3619-5p |
| LINC00355 | hsa-miR-218 |
| LINC00355 | hsa-miR-218a |
| LINC00355 | hsa-miR-223 |
| LINC00355 | hsa-miR-122 |
| LINC00355 | hsa-miR-122a |
| LINC00355 | hsa-miR-1352 |
| LINC00355 | hsa-miR-27abc |
| LINC00355 | hsa-miR-27a-3p |
| LINC00355 | hsa-miR-103a |
| LINC00355 | hsa-miR-107 |
| LINC00355 | hsa-miR-107ab |
| LINC00355 | hsa-miR-124 |
| LINC00355 | hsa-miR-124ab |
| LINC00355 | hsa-miR-506 |
| LINC00355 | hsa-miR-338 |
| LINC00355 | hsa-miR-338-3p |
| LINC00355 | hsa-miR-34ac |
| LINC00355 | hsa-miR-34bc-5p |
| LINC00355 | hsa-miR-449abc |
| LINC00355 | hsa-miR-449c-5p |
| LINC00355 | hsa-miR-10abc |
| LINC00355 | hsa-miR-10a-5p |
| LINC00355 | hsa-miR-455-5p |
| LINC00355 | hsa-miR-128 |
| LINC00355 | hsa-miR-128ab |
| LMO7-AS1 | hsa-miR-7 |
| LMO7-AS1 | hsa-miR-7ab |
| LMO7-AS1 | hsa-miR-93 |
| LMO7-AS1 | hsa-miR-93a |
| LMO7-AS1 | hsa-miR-105 |
| LMO7-AS1 | hsa-miR-106a |
| LMO7-AS1 | hsa-miR-291a-3p |
| LMO7-AS1 | hsa-miR-294 |
| LMO7-AS1 | hsa-miR-295 |
| LMO7-AS1 | hsa-miR-302abcde |
| LMO7-AS1 | hsa-miR-372 |
| LMO7-AS1 | hsa-miR-373 |
| LMO7-AS1 | hsa-miR-428 |
| LMO7-AS1 | hsa-miR-519a |
| LMO7-AS1 | hsa-miR-520be |
| LMO7-AS1 | hsa-miR-520acd-3p |
| LMO7-AS1 | hsa-miR-1378 |
| LMO7-AS1 | hsa-miR-1420ac |
| LMO7-AS1 | hsa-miR-137 |
| LMO7-AS1 | hsa-miR-137ab |
| LMO7-AS1 | hsa-miR-145 |
| LMO7-AS1 | hsa-miR-150 |
| LMO7-AS1 | hsa-miR-5127 |
| LMO7-AS1 | hsa-miR-153 |
| LMO7-AS1 | hsa-miR-17 |
| LMO7-AS1 | hsa-miR-17-5p |
| LMO7-AS1 | hsa-miR-20ab |
| LMO7-AS1 | hsa-miR-20b-5p |
| LMO7-AS1 | hsa-miR-106ab |
| LMO7-AS1 | hsa-miR-427 |
| LMO7-AS1 | hsa-miR-518a-3p |
| LMO7-AS1 | hsa-miR-519d |
| LMO7-AS1 | hsa-miR-181abcd |
| LMO7-AS1 | hsa-miR-4262 |
| LMO7-AS1 | hsa-miR-let-7 |
| LMO7-AS1 | hsa-miR-98 |
| LMO7-AS1 | hsa-miR-4458 |
| LMO7-AS1 | hsa-miR-4500 |
| LMO7-AS1 | hsa-miR-190 |
| LMO7-AS1 | hsa-miR-190ab |
| LMO7-AS1 | hsa-miR-193 |
| LMO7-AS1 | hsa-miR-193b |
| LMO7-AS1 | hsa-miR-193a-3p |
| LMO7-AS1 | hsa-miR-196abc |
| LMO7-AS1 | hsa-miR-204 |
| LMO7-AS1 | hsa-miR-204b |
| LMO7-AS1 | hsa-miR-211 |
| LMO7-AS1 | hsa-miR-214 |
| LMO7-AS1 | hsa-miR-761 |
| LMO7-AS1 | hsa-miR-3619-5p |
| LMO7-AS1 | hsa-miR-122 |
| LMO7-AS1 | hsa-miR-122a |
| LMO7-AS1 | hsa-miR-1352 |
| LMO7-AS1 | hsa-miR-24 |
| LMO7-AS1 | hsa-miR-24ab |
| LMO7-AS1 | hsa-miR-24-3p |
| LMO7-AS1 | hsa-miR-375 |
| LMO7-AS1 | hsa-miR-425 |
| LMO7-AS1 | hsa-miR-425-5p |
| LMO7-AS1 | hsa-miR-489 |
| LMO7-AS1 | hsa-miR-128 |
| LMO7-AS1 | hsa-miR-128ab |
| HOTAIR | hsa-miR-130ac |
| HOTAIR | hsa-miR-301ab |
| HOTAIR | hsa-miR-301b |
| HOTAIR | hsa-miR-301b-3p |
| HOTAIR | hsa-miR-454 |
| HOTAIR | hsa-miR-721 |
| HOTAIR | hsa-miR-4295 |
| HOTAIR | hsa-miR-3666 |
| HOTAIR | hsa-miR-133abc |
| HOTAIR | hsa-miR-9 |
| HOTAIR | hsa-miR-9ab |
| HOTAIR | hsa-miR-138 |
| HOTAIR | hsa-miR-138ab |
| HOTAIR | hsa-miR-143 |
| HOTAIR | hsa-miR-1721 |
| HOTAIR | hsa-miR-4770 |
| HOTAIR | hsa-miR-146ac |
| HOTAIR | hsa-miR-146b-5p |
| HOTAIR | hsa-miR-148ab-3p |
| HOTAIR | hsa-miR-152 |
| HOTAIR | hsa-miR-150 |
| HOTAIR | hsa-miR-5127 |
| HOTAIR | hsa-miR-17 |
| HOTAIR | hsa-miR-17-5p |
| HOTAIR | hsa-miR-20ab |
| HOTAIR | hsa-miR-20b-5p |
| HOTAIR | hsa-miR-93 |
| HOTAIR | hsa-miR-106ab |
| HOTAIR | hsa-miR-427 |
| HOTAIR | hsa-miR-518a-3p |
| HOTAIR | hsa-miR-519d |
| HOTAIR | hsa-miR-193 |
| HOTAIR | hsa-miR-193b |
| HOTAIR | hsa-miR-193a-3p |
| HOTAIR | hsa-miR-194 |
| HOTAIR | hsa-miR-19ab |
| HOTAIR | hsa-miR-1ab |
| HOTAIR | hsa-miR-206 |
| HOTAIR | hsa-miR-613 |
| HOTAIR | hsa-miR-203 |
| HOTAIR | hsa-miR-204 |
| HOTAIR | hsa-miR-204b |
| HOTAIR | hsa-miR-211 |
| HOTAIR | hsa-miR-208ab |
| HOTAIR | hsa-miR-208ab-3p |
| HOTAIR | hsa-miR-21 |
| HOTAIR | hsa-miR-590-5p |
| HOTAIR | hsa-miR-214 |
| HOTAIR | hsa-miR-761 |
| HOTAIR | hsa-miR-3619-5p |
| HOTAIR | hsa-miR-216a |
| HOTAIR | hsa-miR-216b |
| HOTAIR | hsa-miR-216b-5p |
| HOTAIR | hsa-miR-217 |
| HOTAIR | hsa-miR-221 |
| HOTAIR | hsa-miR-222 |
| HOTAIR | hsa-miR-222ab |
| HOTAIR | hsa-miR-1928 |
| HOTAIR | hsa-miR-30abcdef |
| HOTAIR | hsa-miR-30abe-5p |
| HOTAIR | hsa-miR-384-5p |
| HOTAIR | hsa-miR-103a |
| HOTAIR | hsa-miR-107 |
| HOTAIR | hsa-miR-107ab |
| HOTAIR | hsa-miR-124 |
| HOTAIR | hsa-miR-124ab |
| HOTAIR | hsa-miR-506 |
| HOTAIR | hsa-miR-33ab |
| HOTAIR | hsa-miR-33-5p |
| HOTAIR | hsa-miR-34ac |
| HOTAIR | hsa-miR-34bc-5p |
| HOTAIR | hsa-miR-449abc |
| HOTAIR | hsa-miR-449c-5p |
| HOTAIR | hsa-miR-375 |
| HOTAIR | hsa-miR-10abc |
| HOTAIR | hsa-miR-10a-5p |
| HOTAIR | hsa-miR-129-5p |
| HOTAIR | hsa-miR-129ab-5p |
| HOTAIR | hsa-miR-499-5p |
| COX4I1P2 | hsa-miR-205 |
| COX4I1P2 | hsa-miR-205ab |
| COX4I1P2 | hsa-miR-26ab |
| COX4I1P2 | hsa-miR-1297 |
| COX4I1P2 | hsa-miR-4465 |
| COX4I1P2 | hsa-miR-34ac |
| COX4I1P2 | hsa-miR-34bc-5p |
| COX4I1P2 | hsa-miR-449abc |
| COX4I1P2 | hsa-miR-449c-5p |
| COX4I1P2 | hsa-miR-383 |
| COX4I1P2 | hsa-miR-128 |
| COX4I1P2 | hsa-miR-128ab |
| SNRPD2P1 | hsa-miR-205 |
| SNRPD2P1 | hsa-miR-205ab |
| SNRPD2P1 | hsa-miR-29abcd |
| SNRPD2P1 | hsa-miR-338 |
| SNRPD2P1 | hsa-miR-338-3p |
| UGT1A12P | hsa-miR-96 |
| UGT1A12P | hsa-miR-507 |
| UGT1A12P | hsa-miR-1271 |
| UGT1A12P | hsa-miR-146ac |
| UGT1A12P | hsa-miR-146b-5p |
| UGT1A12P | hsa-miR-182 |
| UGT1A12P | hsa-miR-19ab |
| UGT1A12P | hsa-miR-124 |
| UGT1A12P | hsa-miR-124ab |
| UGT1A12P | hsa-miR-506 |
| UGT1A12P | hsa-miR-125a-5p |
| UGT1A12P | hsa-miR-125b-5p |
| UGT1A12P | hsa-miR-351 |
| UGT1A12P | hsa-miR-670 |
| UGT1A12P | hsa-miR-4319 |
| CDC42-IT1 | hsa-miR-143 |
| CDC42-IT1 | hsa-miR-1721 |
| CDC42-IT1 | hsa-miR-4770 |
| CDC42-IT1 | hsa-miR-155 |
| CDC42-IT1 | hsa-miR-194 |
| CDC42-IT1 | hsa-miR-203 |
| CDC42-IT1 | hsa-miR-204 |
| CDC42-IT1 | hsa-miR-204b |
| CDC42-IT1 | hsa-miR-211 |
| CDC42-IT1 | hsa-miR-24 |
| CDC42-IT1 | hsa-miR-24ab |
| CDC42-IT1 | hsa-miR-24-3p |
| CDC42-IT1 | hsa-miR-31 |
| CDC42-IT1 | hsa-miR-338 |
| CDC42-IT1 | hsa-miR-338-3p |
| CDC42-IT1 | hsa-miR-33a-3p |
| CDC42-IT1 | hsa-miR-365 |
| CDC42-IT1 | hsa-miR-365-3p |
| CDC42-IT1 | hsa-miR-126-3p |
| CDC42-IT1 | hsa-miR-490-3p |
| PHBP4 | hsa-miR-9 |
| PHBP4 | hsa-miR-9ab |
| PHBP4 | hsa-miR-138 |
| PHBP4 | hsa-miR-138ab |
| PHBP4 | hsa-miR-1ab |
| PHBP4 | hsa-miR-206 |
| PHBP4 | hsa-miR-613 |
| PHBP4 | hsa-miR-124 |
| PHBP4 | hsa-miR-124ab |
| PHBP4 | hsa-miR-506 |
| PHBP4 | hsa-miR-338 |
| PHBP4 | hsa-miR-338-3p |
| PHBP4 | hsa-miR-383 |
| SPATA20P1 | hsa-miR-9 |
| SPATA20P1 | hsa-miR-9ab |
| SPATA20P1 | hsa-miR-19ab |
| SPATA20P1 | hsa-miR-1ab |
| SPATA20P1 | hsa-miR-206 |
| SPATA20P1 | hsa-miR-613 |
| SPATA20P1 | hsa-miR-27abc |
| SPATA20P1 | hsa-miR-27a-3p |
| SPATA20P1 | hsa-miR-29abcd |
| RPS3AP3 | hsa-miR-145 |
| RPS3AP3 | hsa-miR-204 |
| RPS3AP3 | hsa-miR-204b |
| RPS3AP3 | hsa-miR-211 |
| EZH2P1 | hsa-miR-153 |
| EZH2P1 | hsa-miR-26ab |
| EZH2P1 | hsa-miR-1297 |
| EZH2P1 | hsa-miR-4465 |
| TBL1XR1-AS1 | hsa-miR-96 |
| TBL1XR1-AS1 | hsa-miR-507 |
| TBL1XR1-AS1 | hsa-miR-1271 |
| TBL1XR1-AS1 | hsa-miR-182 |
| TBL1XR1-AS1 | hsa-miR-205 |
| TBL1XR1-AS1 | hsa-miR-205ab |
| TBL1XR1-AS1 | hsa-miR-218 |
| TBL1XR1-AS1 | hsa-miR-218a |
| TBL1XR1-AS1 | hsa-miR-223 |
| TBL1XR1-AS1 | hsa-miR-128 |
| TBL1XR1-AS1 | hsa-miR-128ab |
| TBL1XR1-AS1 | hsa-miR-499-5p |
| ABCA9-AS1 | hsa-miR-15abc |
| ABCA9-AS1 | hsa-miR-16 |
| ABCA9-AS1 | hsa-miR-16abc |
| ABCA9-AS1 | hsa-miR-195 |
| ABCA9-AS1 | hsa-miR-322 |
| ABCA9-AS1 | hsa-miR-424 |
| ABCA9-AS1 | hsa-miR-497 |
| ABCA9-AS1 | hsa-miR-1907 |
| ABCA9-AS1 | hsa-miR-let-7 |
| ABCA9-AS1 | hsa-miR-98 |
| ABCA9-AS1 | hsa-miR-4458 |
| ABCA9-AS1 | hsa-miR-4500 |
| ABCA9-AS1 | hsa-miR-24 |
| ABCA9-AS1 | hsa-miR-24ab |
| ABCA9-AS1 | hsa-miR-24-3p |
| ABCA9-AS1 | hsa-miR-103a |
| ABCA9-AS1 | hsa-miR-107 |
| ABCA9-AS1 | hsa-miR-107ab |
| DLX6-AS1 | hsa-miR-503 |
| DLX6-AS1 | hsa-miR-7 |
| DLX6-AS1 | hsa-miR-7ab |
| DLX6-AS1 | hsa-miR-133abc |
| DLX6-AS1 | hsa-miR-93 |
| DLX6-AS1 | hsa-miR-93a |
| DLX6-AS1 | hsa-miR-105 |
| DLX6-AS1 | hsa-miR-106a |
| DLX6-AS1 | hsa-miR-291a-3p |
| DLX6-AS1 | hsa-miR-294 |
| DLX6-AS1 | hsa-miR-295 |
| DLX6-AS1 | hsa-miR-302abcde |
| DLX6-AS1 | hsa-miR-372 |
| DLX6-AS1 | hsa-miR-373 |
| DLX6-AS1 | hsa-miR-428 |
| DLX6-AS1 | hsa-miR-519a |
| DLX6-AS1 | hsa-miR-520be |
| DLX6-AS1 | hsa-miR-520acd-3p |
| DLX6-AS1 | hsa-miR-1378 |
| DLX6-AS1 | hsa-miR-1420ac |
| DLX6-AS1 | hsa-miR-99ab |
| DLX6-AS1 | hsa-miR-100 |
| DLX6-AS1 | hsa-miR-135ab |
| DLX6-AS1 | hsa-miR-135a-5p |
| DLX6-AS1 | hsa-miR-139-5p |
| DLX6-AS1 | hsa-miR-141 |
| DLX6-AS1 | hsa-miR-200a |
| DLX6-AS1 | hsa-miR-142-3p |
| DLX6-AS1 | hsa-miR-144 |
| DLX6-AS1 | hsa-miR-145 |
| DLX6-AS1 | hsa-miR-148ab-3p |
| DLX6-AS1 | hsa-miR-152 |
| DLX6-AS1 | hsa-miR-150 |
| DLX6-AS1 | hsa-miR-5127 |
| DLX6-AS1 | hsa-miR-155 |
| DLX6-AS1 | hsa-miR-15abc |
| DLX6-AS1 | hsa-miR-16 |
| DLX6-AS1 | hsa-miR-16abc |
| DLX6-AS1 | hsa-miR-195 |
| DLX6-AS1 | hsa-miR-322 |
| DLX6-AS1 | hsa-miR-424 |
| DLX6-AS1 | hsa-miR-497 |
| DLX6-AS1 | hsa-miR-1907 |
| DLX6-AS1 | hsa-miR-17 |
| DLX6-AS1 | hsa-miR-17-5p |
| DLX6-AS1 | hsa-miR-20ab |
| DLX6-AS1 | hsa-miR-20b-5p |
| DLX6-AS1 | hsa-miR-106ab |
| DLX6-AS1 | hsa-miR-427 |
| DLX6-AS1 | hsa-miR-518a-3p |
| DLX6-AS1 | hsa-miR-519d |
| DLX6-AS1 | hsa-miR-181abcd |
| DLX6-AS1 | hsa-miR-4262 |
| DLX6-AS1 | hsa-miR-192 |
| DLX6-AS1 | hsa-miR-215 |
| DLX6-AS1 | hsa-miR-193 |
| DLX6-AS1 | hsa-miR-193b |
| DLX6-AS1 | hsa-miR-193a-3p |
| DLX6-AS1 | hsa-miR-196abc |
| DLX6-AS1 | hsa-miR-199ab-5p |
| DLX6-AS1 | hsa-miR-1ab |
| DLX6-AS1 | hsa-miR-206 |
| DLX6-AS1 | hsa-miR-613 |
| DLX6-AS1 | hsa-miR-200bc |
| DLX6-AS1 | hsa-miR-429 |
| DLX6-AS1 | hsa-miR-548a |
| DLX6-AS1 | hsa-miR-203 |
| DLX6-AS1 | hsa-miR-204 |
| DLX6-AS1 | hsa-miR-204b |
| DLX6-AS1 | hsa-miR-211 |
| DLX6-AS1 | hsa-miR-214 |
| DLX6-AS1 | hsa-miR-761 |
| DLX6-AS1 | hsa-miR-3619-5p |
| DLX6-AS1 | hsa-miR-216a |
| DLX6-AS1 | hsa-miR-216b |
| DLX6-AS1 | hsa-miR-216b-5p |
| DLX6-AS1 | hsa-miR-22 |
| DLX6-AS1 | hsa-miR-22-3p |
| DLX6-AS1 | hsa-miR-223 |
| DLX6-AS1 | hsa-miR-122 |
| DLX6-AS1 | hsa-miR-122a |
| DLX6-AS1 | hsa-miR-1352 |
| DLX6-AS1 | hsa-miR-23abc |
| DLX6-AS1 | hsa-miR-23b-3p |
| DLX6-AS1 | hsa-miR-24 |
| DLX6-AS1 | hsa-miR-24ab |
| DLX6-AS1 | hsa-miR-24-3p |
| DLX6-AS1 | hsa-miR-26ab |
| DLX6-AS1 | hsa-miR-1297 |
| DLX6-AS1 | hsa-miR-4465 |
| DLX6-AS1 | hsa-miR-27abc |
| DLX6-AS1 | hsa-miR-27a-3p |
| DLX6-AS1 | hsa-miR-29abcd |
| DLX6-AS1 | hsa-miR-31 |
| DLX6-AS1 | hsa-miR-103a |
| DLX6-AS1 | hsa-miR-107 |
| DLX6-AS1 | hsa-miR-107ab |
| DLX6-AS1 | hsa-miR-124 |
| DLX6-AS1 | hsa-miR-124ab |
| DLX6-AS1 | hsa-miR-506 |
| DLX6-AS1 | hsa-miR-338 |
| DLX6-AS1 | hsa-miR-338-3p |
| DLX6-AS1 | hsa-miR-383 |
| DLX6-AS1 | hsa-miR-125a-5p |
| DLX6-AS1 | hsa-miR-125b-5p |
| DLX6-AS1 | hsa-miR-351 |
| DLX6-AS1 | hsa-miR-670 |
| DLX6-AS1 | hsa-miR-4319 |
| DLX6-AS1 | hsa-miR-10abc |
| DLX6-AS1 | hsa-miR-10a-5p |
| DLX6-AS1 | hsa-miR-128 |
| DLX6-AS1 | hsa-miR-128ab |
| DLX6-AS1 | hsa-miR-129-5p |
| DLX6-AS1 | hsa-miR-129ab-5p |
| DLX6-AS1 | hsa-miR-490-3p |
| SNRPFP1 | hsa-miR-139-5p |
| BTF3L4P1 | hsa-miR-146ac |
| BTF3L4P1 | hsa-miR-146b-5p |
| BTF3L4P1 | hsa-miR-27abc |
| BTF3L4P1 | hsa-miR-27a-3p |
| BTF3L4P1 | hsa-miR-29abcd |
| OSBPL10-AS1 | hsa-miR-503 |
| OSBPL10-AS1 | hsa-miR-7 |
| OSBPL10-AS1 | hsa-miR-7ab |
| OSBPL10-AS1 | hsa-miR-133abc |
| OSBPL10-AS1 | hsa-miR-96 |
| OSBPL10-AS1 | hsa-miR-507 |
| OSBPL10-AS1 | hsa-miR-1271 |
| OSBPL10-AS1 | hsa-miR-135ab |
| OSBPL10-AS1 | hsa-miR-135a-5p |
| OSBPL10-AS1 | hsa-miR-138 |
| OSBPL10-AS1 | hsa-miR-138ab |
| OSBPL10-AS1 | hsa-miR-145 |
| OSBPL10-AS1 | hsa-miR-146ac |
| OSBPL10-AS1 | hsa-miR-146b-5p |
| OSBPL10-AS1 | hsa-miR-155 |
| OSBPL10-AS1 | hsa-miR-15abc |
| OSBPL10-AS1 | hsa-miR-16 |
| OSBPL10-AS1 | hsa-miR-16abc |
| OSBPL10-AS1 | hsa-miR-195 |
| OSBPL10-AS1 | hsa-miR-322 |
| OSBPL10-AS1 | hsa-miR-424 |
| OSBPL10-AS1 | hsa-miR-497 |
| OSBPL10-AS1 | hsa-miR-1907 |
| OSBPL10-AS1 | hsa-miR-182 |
| OSBPL10-AS1 | hsa-miR-204 |
| OSBPL10-AS1 | hsa-miR-204b |
| OSBPL10-AS1 | hsa-miR-211 |
| OSBPL10-AS1 | hsa-miR-214 |
| OSBPL10-AS1 | hsa-miR-761 |
| OSBPL10-AS1 | hsa-miR-3619-5p |
| OSBPL10-AS1 | hsa-miR-216a |
| OSBPL10-AS1 | hsa-miR-216b |
| OSBPL10-AS1 | hsa-miR-216b-5p |
| OSBPL10-AS1 | hsa-miR-23abc |
| OSBPL10-AS1 | hsa-miR-23b-3p |
| OSBPL10-AS1 | hsa-miR-26ab |
| OSBPL10-AS1 | hsa-miR-1297 |
| OSBPL10-AS1 | hsa-miR-4465 |
| OSBPL10-AS1 | hsa-miR-27abc |
| OSBPL10-AS1 | hsa-miR-27a-3p |
| OSBPL10-AS1 | hsa-miR-124 |
| OSBPL10-AS1 | hsa-miR-124ab |
| OSBPL10-AS1 | hsa-miR-506 |
| OSBPL10-AS1 | hsa-miR-375 |
| OSBPL10-AS1 | hsa-miR-125a-5p |
| OSBPL10-AS1 | hsa-miR-125b-5p |
| OSBPL10-AS1 | hsa-miR-351 |
| OSBPL10-AS1 | hsa-miR-670 |
| OSBPL10-AS1 | hsa-miR-4319 |
| OSBPL10-AS1 | hsa-miR-451 |
| OSBPL10-AS1 | hsa-miR-128 |
| OSBPL10-AS1 | hsa-miR-128ab |
| OSBPL10-AS1 | hsa-miR-490-3p |
| SYNJ2-IT1 | hsa-miR-551a |
| SYNJ2-IT1 | hsa-miR-132 |
| SYNJ2-IT1 | hsa-miR-212 |
| SYNJ2-IT1 | hsa-miR-212-3p |
| SYNJ2-IT1 | hsa-miR-9 |
| SYNJ2-IT1 | hsa-miR-9ab |
| SYNJ2-IT1 | hsa-miR-143 |
| SYNJ2-IT1 | hsa-miR-1721 |
| SYNJ2-IT1 | hsa-miR-4770 |
| SYNJ2-IT1 | hsa-miR-181abcd |
| SYNJ2-IT1 | hsa-miR-4262 |
| SYNJ2-IT1 | hsa-miR-218 |
| SYNJ2-IT1 | hsa-miR-218a |
| SYNJ2-IT1 | hsa-miR-221 |
| SYNJ2-IT1 | hsa-miR-222 |
| SYNJ2-IT1 | hsa-miR-222ab |
| SYNJ2-IT1 | hsa-miR-1928 |
| SYNJ2-IT1 | hsa-miR-24 |
| SYNJ2-IT1 | hsa-miR-24ab |
| SYNJ2-IT1 | hsa-miR-24-3p |
| SYNJ2-IT1 | hsa-miR-10abc |
| SYNJ2-IT1 | hsa-miR-10a-5p |
| SYNJ2-IT1 | hsa-miR-129-5p |
| SYNJ2-IT1 | hsa-miR-129ab-5p |
| LINC00460 | hsa-miR-503 |
| LINC00460 | hsa-miR-143 |
| LINC00460 | hsa-miR-1721 |
| LINC00460 | hsa-miR-4770 |
| LINC00460 | hsa-miR-150 |
| LINC00460 | hsa-miR-5127 |
| LINC00460 | hsa-miR-1ab |
| LINC00460 | hsa-miR-206 |
| LINC00460 | hsa-miR-613 |
| LINC00460 | hsa-miR-200bc |
| LINC00460 | hsa-miR-429 |
| LINC00460 | hsa-miR-548a |
| LINC00460 | hsa-miR-221 |
| LINC00460 | hsa-miR-222 |
| LINC00460 | hsa-miR-222ab |
| LINC00460 | hsa-miR-1928 |
| LINC00460 | hsa-miR-23abc |
| LINC00460 | hsa-miR-23b-3p |
| LINC00460 | hsa-miR-24 |
| LINC00460 | hsa-miR-24ab |
| LINC00460 | hsa-miR-24-3p |
| LINC00460 | hsa-miR-103a |
| LINC00460 | hsa-miR-107 |
| LINC00460 | hsa-miR-107ab |
| LINC00460 | hsa-miR-338 |
| LINC00460 | hsa-miR-338-3p |
| LINC00460 | hsa-miR-425 |
| LINC00460 | hsa-miR-425-5p |
| LINC00460 | hsa-miR-489 |
| LINC00460 | hsa-miR-129-5p |
| LINC00460 | hsa-miR-129ab-5p |
| GAS6-AS1 | hsa-miR-96 |
| GAS6-AS1 | hsa-miR-507 |
| GAS6-AS1 | hsa-miR-1271 |
| GAS6-AS1 | hsa-miR-182 |
| GAS6-AS1 | hsa-miR-19ab |
| GAS6-AS1 | hsa-miR-205 |
| GAS6-AS1 | hsa-miR-205ab |
| GAS6-AS1 | hsa-miR-214 |
| GAS6-AS1 | hsa-miR-761 |
| GAS6-AS1 | hsa-miR-3619-5p |
| GAS6-AS1 | hsa-miR-218 |
| GAS6-AS1 | hsa-miR-218a |
| GAS6-AS1 | hsa-miR-223 |
| GAS6-AS1 | hsa-miR-25 |
| GAS6-AS1 | hsa-miR-32 |
| GAS6-AS1 | hsa-miR-92abc |
| GAS6-AS1 | hsa-miR-363 |
| GAS6-AS1 | hsa-miR-363-3p |
| GAS6-AS1 | hsa-miR-367 |
| GAS6-AS1 | hsa-miR-124 |
| GAS6-AS1 | hsa-miR-124ab |
| GAS6-AS1 | hsa-miR-506 |
| SETP5 | hsa-miR-145 |
| SETP5 | hsa-miR-148ab-3p |
| SETP5 | hsa-miR-152 |
| SETP5 | hsa-miR-150 |
| SETP5 | hsa-miR-5127 |
| SETP5 | hsa-miR-193 |
| SETP5 | hsa-miR-193b |
| SETP5 | hsa-miR-193a-3p |
| SETP5 | hsa-miR-490-3p |
| MTND4P26 | hsa-miR-132 |
| MTND4P26 | hsa-miR-212 |
| MTND4P26 | hsa-miR-212-3p |
| MTND4P26 | hsa-miR-93 |
| MTND4P26 | hsa-miR-93a |
| MTND4P26 | hsa-miR-105 |
| MTND4P26 | hsa-miR-106a |
| MTND4P26 | hsa-miR-291a-3p |
| MTND4P26 | hsa-miR-294 |
| MTND4P26 | hsa-miR-295 |
| MTND4P26 | hsa-miR-302abcde |
| MTND4P26 | hsa-miR-372 |
| MTND4P26 | hsa-miR-373 |
| MTND4P26 | hsa-miR-428 |
| MTND4P26 | hsa-miR-519a |
| MTND4P26 | hsa-miR-520be |
| MTND4P26 | hsa-miR-520acd-3p |
| MTND4P26 | hsa-miR-1378 |
| MTND4P26 | hsa-miR-1420ac |
| MTND4P26 | hsa-miR-135ab |
| MTND4P26 | hsa-miR-135a-5p |
| MTND4P26 | hsa-miR-144 |
| MTND4P26 | hsa-miR-146ac |
| MTND4P26 | hsa-miR-146b-5p |
| MTND4P26 | hsa-miR-184 |
| MTND4P26 | hsa-miR-let-7 |
| MTND4P26 | hsa-miR-98 |
| MTND4P26 | hsa-miR-4458 |
| MTND4P26 | hsa-miR-4500 |
| MTND4P26 | hsa-miR-194 |
| MTND4P26 | hsa-miR-200bc |
| MTND4P26 | hsa-miR-429 |
| MTND4P26 | hsa-miR-548a |
| MTND4P26 | hsa-miR-30abcdef |
| MTND4P26 | hsa-miR-30abe-5p |
| MTND4P26 | hsa-miR-384-5p |
| MTND4P26 | hsa-miR-124 |
| MTND4P26 | hsa-miR-124ab |
| MTND4P26 | hsa-miR-506 |
| MTND4P26 | hsa-miR-383 |
| PCMTD1P3 | hsa-miR-145 |
| PCMTD1P3 | hsa-miR-210 |
| PCMTD1P3 | hsa-miR-26ab |
| PCMTD1P3 | hsa-miR-1297 |
| PCMTD1P3 | hsa-miR-4465 |
| PCMTD1P3 | hsa-miR-425 |
| PCMTD1P3 | hsa-miR-425-5p |
| PCMTD1P3 | hsa-miR-489 |
| MYO16-AS1 | hsa-miR-208ab |
| MYO16-AS1 | hsa-miR-208ab-3p |
| MYO16-AS1 | hsa-miR-23abc |
| MYO16-AS1 | hsa-miR-23b-3p |
| MYO16-AS1 | hsa-miR-338 |
| MYO16-AS1 | hsa-miR-338-3p |
| MYO16-AS1 | hsa-miR-425 |
| MYO16-AS1 | hsa-miR-425-5p |
| MYO16-AS1 | hsa-miR-489 |
| MYO16-AS1 | hsa-miR-125a-5p |
| MYO16-AS1 | hsa-miR-125b-5p |
| MYO16-AS1 | hsa-miR-351 |
| MYO16-AS1 | hsa-miR-670 |
| MYO16-AS1 | hsa-miR-4319 |
| MYO16-AS1 | hsa-miR-129-5p |
| MYO16-AS1 | hsa-miR-129ab-5p |
| MYO16-AS1 | hsa-miR-499-5p |
| DIAPH2-AS1 | hsa-miR-9 |
| DIAPH2-AS1 | hsa-miR-9ab |
| DIAPH2-AS1 | hsa-miR-140 |
| DIAPH2-AS1 | hsa-miR-140-5p |
| DIAPH2-AS1 | hsa-miR-876-3p |
| DIAPH2-AS1 | hsa-miR-1244 |
| DIAPH2-AS1 | hsa-miR-153 |
| DIAPH2-AS1 | hsa-miR-181abcd |
| DIAPH2-AS1 | hsa-miR-4262 |
| DIAPH2-AS1 | hsa-miR-183 |
| DIAPH2-AS1 | hsa-miR-18ab |
| DIAPH2-AS1 | hsa-miR-4735-3p |
| DIAPH2-AS1 | hsa-miR-190 |
| DIAPH2-AS1 | hsa-miR-190ab |
| DIAPH2-AS1 | hsa-miR-200bc |
| DIAPH2-AS1 | hsa-miR-429 |
| DIAPH2-AS1 | hsa-miR-548a |
| DIAPH2-AS1 | hsa-miR-204 |
| DIAPH2-AS1 | hsa-miR-204b |
| DIAPH2-AS1 | hsa-miR-211 |
| DIAPH2-AS1 | hsa-miR-217 |
| DIAPH2-AS1 | hsa-miR-122 |
| DIAPH2-AS1 | hsa-miR-122a |
| DIAPH2-AS1 | hsa-miR-1352 |
| DIAPH2-AS1 | hsa-miR-23abc |
| DIAPH2-AS1 | hsa-miR-23b-3p |
| DIAPH2-AS1 | hsa-miR-34ac |
| DIAPH2-AS1 | hsa-miR-34bc-5p |
| DIAPH2-AS1 | hsa-miR-449abc |
| DIAPH2-AS1 | hsa-miR-449c-5p |
| DIAPH2-AS1 | hsa-miR-425 |
| DIAPH2-AS1 | hsa-miR-425-5p |
| DIAPH2-AS1 | hsa-miR-489 |
| DIAPH2-AS1 | hsa-miR-129-5p |
| DIAPH2-AS1 | hsa-miR-129ab-5p |
| SULT1C2P1 | hsa-miR-132 |
| SULT1C2P1 | hsa-miR-212 |
| SULT1C2P1 | hsa-miR-212-3p |
| SULT1C2P1 | hsa-miR-9 |
| SULT1C2P1 | hsa-miR-9ab |
| SULT1C2P1 | hsa-miR-93 |
| SULT1C2P1 | hsa-miR-93a |
| SULT1C2P1 | hsa-miR-105 |
| SULT1C2P1 | hsa-miR-106a |
| SULT1C2P1 | hsa-miR-291a-3p |
| SULT1C2P1 | hsa-miR-294 |
| SULT1C2P1 | hsa-miR-295 |
| SULT1C2P1 | hsa-miR-302abcde |
| SULT1C2P1 | hsa-miR-372 |
| SULT1C2P1 | hsa-miR-373 |
| SULT1C2P1 | hsa-miR-428 |
| SULT1C2P1 | hsa-miR-519a |
| SULT1C2P1 | hsa-miR-520be |
| SULT1C2P1 | hsa-miR-520acd-3p |
| SULT1C2P1 | hsa-miR-1378 |
| SULT1C2P1 | hsa-miR-1420ac |
| SULT1C2P1 | hsa-miR-96 |
| SULT1C2P1 | hsa-miR-507 |
| SULT1C2P1 | hsa-miR-1271 |
| SULT1C2P1 | hsa-miR-140 |
| SULT1C2P1 | hsa-miR-140-5p |
| SULT1C2P1 | hsa-miR-876-3p |
| SULT1C2P1 | hsa-miR-1244 |
| SULT1C2P1 | hsa-miR-143 |
| SULT1C2P1 | hsa-miR-1721 |
| SULT1C2P1 | hsa-miR-4770 |
| SULT1C2P1 | hsa-miR-146ac |
| SULT1C2P1 | hsa-miR-146b-5p |
| SULT1C2P1 | hsa-miR-182 |
| SULT1C2P1 | hsa-miR-192 |
| SULT1C2P1 | hsa-miR-215 |
| SULT1C2P1 | hsa-miR-196abc |
| SULT1C2P1 | hsa-miR-1ab |
| SULT1C2P1 | hsa-miR-206 |
| SULT1C2P1 | hsa-miR-613 |
| SULT1C2P1 | hsa-miR-205 |
| SULT1C2P1 | hsa-miR-205ab |
| SULT1C2P1 | hsa-miR-208ab |
| SULT1C2P1 | hsa-miR-208ab-3p |
| SULT1C2P1 | hsa-miR-21 |
| SULT1C2P1 | hsa-miR-590-5p |
| SULT1C2P1 | hsa-miR-214 |
| SULT1C2P1 | hsa-miR-761 |
| SULT1C2P1 | hsa-miR-3619-5p |
| SULT1C2P1 | hsa-miR-22 |
| SULT1C2P1 | hsa-miR-22-3p |
| SULT1C2P1 | hsa-miR-221 |
| SULT1C2P1 | hsa-miR-222 |
| SULT1C2P1 | hsa-miR-222ab |
| SULT1C2P1 | hsa-miR-1928 |
| SULT1C2P1 | hsa-miR-23abc |
| SULT1C2P1 | hsa-miR-23b-3p |
| SULT1C2P1 | hsa-miR-27abc |
| SULT1C2P1 | hsa-miR-27a-3p |
| SULT1C2P1 | hsa-miR-34ac |
| SULT1C2P1 | hsa-miR-34bc-5p |
| SULT1C2P1 | hsa-miR-449abc |
| SULT1C2P1 | hsa-miR-449c-5p |
| SULT1C2P1 | hsa-miR-455-5p |
| SULT1C2P1 | hsa-miR-128 |
| SULT1C2P1 | hsa-miR-128ab |
| SULT1C2P1 | hsa-miR-499-5p |
| CAMTA1-IT1 | hsa-miR-150 |
| CAMTA1-IT1 | hsa-miR-5127 |
| CAMTA1-IT1 | hsa-miR-1ab |
| CAMTA1-IT1 | hsa-miR-206 |
| CAMTA1-IT1 | hsa-miR-613 |
| CAMTA1-IT1 | hsa-miR-223 |
| ZDHHC4P1 | hsa-miR-7 |
| ZDHHC4P1 | hsa-miR-7ab |
| ZDHHC4P1 | hsa-miR-135ab |
| ZDHHC4P1 | hsa-miR-135a-5p |
| ZDHHC4P1 | hsa-miR-138 |
| ZDHHC4P1 | hsa-miR-138ab |
| ZDHHC4P1 | hsa-miR-146ac |
| ZDHHC4P1 | hsa-miR-146b-5p |
| ZDHHC4P1 | hsa-miR-148ab-3p |
| ZDHHC4P1 | hsa-miR-152 |
| ZDHHC4P1 | hsa-miR-let-7 |
| ZDHHC4P1 | hsa-miR-98 |
| ZDHHC4P1 | hsa-miR-4458 |
| ZDHHC4P1 | hsa-miR-4500 |
| ZDHHC4P1 | hsa-miR-196abc |
| ZDHHC4P1 | hsa-miR-1ab |
| ZDHHC4P1 | hsa-miR-206 |
| ZDHHC4P1 | hsa-miR-613 |
| ZDHHC4P1 | hsa-miR-29abcd |
| ZDHHC4P1 | hsa-miR-338 |
| ZDHHC4P1 | hsa-miR-338-3p |
| ZDHHC4P1 | hsa-miR-33a-3p |
| ZDHHC4P1 | hsa-miR-365 |
| ZDHHC4P1 | hsa-miR-365-3p |
| ABCA17P | hsa-miR-7 |
| ABCA17P | hsa-miR-7ab |
| ABCA17P | hsa-miR-9 |
| ABCA17P | hsa-miR-9ab |
| ABCA17P | hsa-miR-96 |
| ABCA17P | hsa-miR-507 |
| ABCA17P | hsa-miR-1271 |
| ABCA17P | hsa-miR-135ab |
| ABCA17P | hsa-miR-135a-5p |
| ABCA17P | hsa-miR-139-5p |
| ABCA17P | hsa-miR-143 |
| ABCA17P | hsa-miR-1721 |
| ABCA17P | hsa-miR-4770 |
| ABCA17P | hsa-miR-145 |
| ABCA17P | hsa-miR-150 |
| ABCA17P | hsa-miR-5127 |
| ABCA17P | hsa-miR-15abc |
| ABCA17P | hsa-miR-16 |
| ABCA17P | hsa-miR-16abc |
| ABCA17P | hsa-miR-195 |
| ABCA17P | hsa-miR-322 |
| ABCA17P | hsa-miR-424 |
| ABCA17P | hsa-miR-497 |
| ABCA17P | hsa-miR-1907 |
| ABCA17P | hsa-miR-181abcd |
| ABCA17P | hsa-miR-4262 |
| ABCA17P | hsa-miR-193 |
| ABCA17P | hsa-miR-193b |
| ABCA17P | hsa-miR-193a-3p |
| ABCA17P | hsa-miR-199ab-5p |
| ABCA17P | hsa-miR-1ab |
| ABCA17P | hsa-miR-206 |
| ABCA17P | hsa-miR-613 |
| ABCA17P | hsa-miR-203 |
| ABCA17P | hsa-miR-204 |
| ABCA17P | hsa-miR-204b |
| ABCA17P | hsa-miR-211 |
| ABCA17P | hsa-miR-205 |
| ABCA17P | hsa-miR-205ab |
| ABCA17P | hsa-miR-21 |
| ABCA17P | hsa-miR-590-5p |
| ABCA17P | hsa-miR-214 |
| ABCA17P | hsa-miR-761 |
| ABCA17P | hsa-miR-3619-5p |
| ABCA17P | hsa-miR-216b |
| ABCA17P | hsa-miR-216b-5p |
| ABCA17P | hsa-miR-22 |
| ABCA17P | hsa-miR-22-3p |
| ABCA17P | hsa-miR-23abc |
| ABCA17P | hsa-miR-23b-3p |
| ABCA17P | hsa-miR-24 |
| ABCA17P | hsa-miR-24ab |
| ABCA17P | hsa-miR-24-3p |
| ABCA17P | hsa-miR-30abcdef |
| ABCA17P | hsa-miR-30abe-5p |
| ABCA17P | hsa-miR-384-5p |
| ABCA17P | hsa-miR-103a |
| ABCA17P | hsa-miR-107 |
| ABCA17P | hsa-miR-107ab |
| ABCA17P | hsa-miR-124 |
| ABCA17P | hsa-miR-124ab |
| ABCA17P | hsa-miR-506 |
| ABCA17P | hsa-miR-338 |
| ABCA17P | hsa-miR-338-3p |
| ABCA17P | hsa-miR-33ab |
| ABCA17P | hsa-miR-33-5p |
| ABCA17P | hsa-miR-34ac |
| ABCA17P | hsa-miR-34bc-5p |
| ABCA17P | hsa-miR-449abc |
| ABCA17P | hsa-miR-449c-5p |
| ABCA17P | hsa-miR-10abc |
| ABCA17P | hsa-miR-10a-5p |
| ABCA17P | hsa-miR-490-3p |
| ABCA17P | hsa-miR-499-5p |
| SCARNA10 | hsa-miR-7 |
| SCARNA10 | hsa-miR-7ab |
| SCARNA10 | hsa-miR-145 |
| SCARNA10 | hsa-miR-let-7 |
| SCARNA10 | hsa-miR-98 |
| SCARNA10 | hsa-miR-4458 |
| SCARNA10 | hsa-miR-4500 |
| SCARNA10 | hsa-miR-338 |
| SCARNA10 | hsa-miR-338-3p |
| SCARNA10 | hsa-miR-10abc |
| SCARNA10 | hsa-miR-10a-5p |
| RPS2P44 | hsa-miR-143 |
| RPS2P44 | hsa-miR-1721 |
| RPS2P44 | hsa-miR-4770 |
| RPS2P44 | hsa-miR-148ab-3p |
| RPS2P44 | hsa-miR-152 |
| RPS2P44 | hsa-miR-15abc |
| RPS2P44 | hsa-miR-16 |
| RPS2P44 | hsa-miR-16abc |
| RPS2P44 | hsa-miR-195 |
| RPS2P44 | hsa-miR-322 |
| RPS2P44 | hsa-miR-424 |
| RPS2P44 | hsa-miR-497 |
| RPS2P44 | hsa-miR-1907 |
| RPS2P44 | hsa-miR-182 |
| RPS2P44 | hsa-miR-218 |
| RPS2P44 | hsa-miR-218a |
| RPS2P44 | hsa-miR-33ab |
| RPS2P44 | hsa-miR-33-5p |
| RPS2P44 | hsa-miR-34ac |
| RPS2P44 | hsa-miR-34bc-5p |
| RPS2P44 | hsa-miR-449abc |
| RPS2P44 | hsa-miR-449c-5p |
| RPS2P44 | hsa-miR-425 |
| RPS2P44 | hsa-miR-425-5p |
| RPS2P44 | hsa-miR-489 |
| RPS2P44 | hsa-miR-125a-5p |
| RPS2P44 | hsa-miR-125b-5p |
| RPS2P44 | hsa-miR-351 |
| RPS2P44 | hsa-miR-670 |
| RPS2P44 | hsa-miR-4319 |
| CRNDE | hsa-miR-9 |
| CRNDE | hsa-miR-9ab |
| CRNDE | hsa-miR-135ab |
| CRNDE | hsa-miR-135a-5p |
| CRNDE | hsa-miR-140 |
| CRNDE | hsa-miR-140-5p |
| CRNDE | hsa-miR-876-3p |
| CRNDE | hsa-miR-1244 |
| CRNDE | hsa-miR-142-3p |
| CRNDE | hsa-miR-143 |
| CRNDE | hsa-miR-1721 |
| CRNDE | hsa-miR-4770 |
| CRNDE | hsa-miR-144 |
| CRNDE | hsa-miR-145 |
| CRNDE | hsa-miR-146ac |
| CRNDE | hsa-miR-146b-5p |
| CRNDE | hsa-miR-155 |
| CRNDE | hsa-miR-181abcd |
| CRNDE | hsa-miR-4262 |
| CRNDE | hsa-miR-183 |
| CRNDE | hsa-miR-193 |
| CRNDE | hsa-miR-193b |
| CRNDE | hsa-miR-193a-3p |
| CRNDE | hsa-miR-199ab-5p |
| CRNDE | hsa-miR-203 |
| CRNDE | hsa-miR-205 |
| CRNDE | hsa-miR-205ab |
| CRNDE | hsa-miR-216b |
| CRNDE | hsa-miR-216b-5p |
| CRNDE | hsa-miR-217 |
| CRNDE | hsa-miR-219-5p |
| CRNDE | hsa-miR-508 |
| CRNDE | hsa-miR-508-3p |
| CRNDE | hsa-miR-4782-3p |
| CRNDE | hsa-miR-22 |
| CRNDE | hsa-miR-22-3p |
| CRNDE | hsa-miR-221 |
| CRNDE | hsa-miR-222 |
| CRNDE | hsa-miR-222ab |
| CRNDE | hsa-miR-1928 |
| CRNDE | hsa-miR-223 |
| CRNDE | hsa-miR-23abc |
| CRNDE | hsa-miR-23b-3p |
| CRNDE | hsa-miR-25 |
| CRNDE | hsa-miR-32 |
| CRNDE | hsa-miR-92abc |
| CRNDE | hsa-miR-363 |
| CRNDE | hsa-miR-363-3p |
| CRNDE | hsa-miR-367 |
| CRNDE | hsa-miR-26ab |
| CRNDE | hsa-miR-1297 |
| CRNDE | hsa-miR-4465 |
| CRNDE | hsa-miR-27abc |
| CRNDE | hsa-miR-27a-3p |
| CRNDE | hsa-miR-101 |
| CRNDE | hsa-miR-101ab |
| CRNDE | hsa-miR-31 |
| CRNDE | hsa-miR-338 |
| CRNDE | hsa-miR-338-3p |
| CRNDE | hsa-miR-128 |
| CRNDE | hsa-miR-128ab |
| CRNDE | hsa-miR-129-5p |
| CRNDE | hsa-miR-129ab-5p |
| GBA3 | hsa-miR-503 |
| GBA3 | hsa-miR-7 |
| GBA3 | hsa-miR-7ab |
| GBA3 | hsa-miR-138 |
| GBA3 | hsa-miR-138ab |
| GBA3 | hsa-miR-140 |
| GBA3 | hsa-miR-140-5p |
| GBA3 | hsa-miR-876-3p |
| GBA3 | hsa-miR-1244 |
| GBA3 | hsa-miR-15abc |
| GBA3 | hsa-miR-16 |
| GBA3 | hsa-miR-16abc |
| GBA3 | hsa-miR-195 |
| GBA3 | hsa-miR-322 |
| GBA3 | hsa-miR-424 |
| GBA3 | hsa-miR-497 |
| GBA3 | hsa-miR-1907 |
| GBA3 | hsa-miR-194 |
| GBA3 | hsa-miR-19ab |
| GBA3 | hsa-miR-203 |
| GBA3 | hsa-miR-208ab |
| GBA3 | hsa-miR-208ab-3p |
| GBA3 | hsa-miR-21 |
| GBA3 | hsa-miR-590-5p |
| GBA3 | hsa-miR-214 |
| GBA3 | hsa-miR-761 |
| GBA3 | hsa-miR-3619-5p |
| GBA3 | hsa-miR-218 |
| GBA3 | hsa-miR-218a |
| GBA3 | hsa-miR-22 |
| GBA3 | hsa-miR-22-3p |
| GBA3 | hsa-miR-221 |
| GBA3 | hsa-miR-222 |
| GBA3 | hsa-miR-222ab |
| GBA3 | hsa-miR-1928 |
| GBA3 | hsa-miR-25 |
| GBA3 | hsa-miR-32 |
| GBA3 | hsa-miR-92abc |
| GBA3 | hsa-miR-363 |
| GBA3 | hsa-miR-363-3p |
| GBA3 | hsa-miR-367 |
| GBA3 | hsa-miR-33ab |
| GBA3 | hsa-miR-33-5p |
| GBA3 | hsa-miR-34ac |
| GBA3 | hsa-miR-34bc-5p |
| GBA3 | hsa-miR-449abc |
| GBA3 | hsa-miR-449c-5p |
| GBA3 | hsa-miR-425 |
| GBA3 | hsa-miR-425-5p |
| GBA3 | hsa-miR-489 |
| GBA3 | hsa-miR-125a-5p |
| GBA3 | hsa-miR-125b-5p |
| GBA3 | hsa-miR-351 |
| GBA3 | hsa-miR-670 |
| GBA3 | hsa-miR-4319 |
| GBA3 | hsa-miR-499-5p |
| KDELC1P1 | hsa-miR-503 |
| KDELC1P1 | hsa-miR-130ac |
| KDELC1P1 | hsa-miR-301ab |
| KDELC1P1 | hsa-miR-301b |
| KDELC1P1 | hsa-miR-301b-3p |
| KDELC1P1 | hsa-miR-454 |
| KDELC1P1 | hsa-miR-721 |
| KDELC1P1 | hsa-miR-4295 |
| KDELC1P1 | hsa-miR-3666 |
| KDELC1P1 | hsa-miR-132 |
| KDELC1P1 | hsa-miR-212 |
| KDELC1P1 | hsa-miR-212-3p |
| KDELC1P1 | hsa-miR-7 |
| KDELC1P1 | hsa-miR-7ab |
| KDELC1P1 | hsa-miR-133abc |
| KDELC1P1 | hsa-miR-9 |
| KDELC1P1 | hsa-miR-9ab |
| KDELC1P1 | hsa-miR-93 |
| KDELC1P1 | hsa-miR-93a |
| KDELC1P1 | hsa-miR-105 |
| KDELC1P1 | hsa-miR-106a |
| KDELC1P1 | hsa-miR-291a-3p |
| KDELC1P1 | hsa-miR-294 |
| KDELC1P1 | hsa-miR-295 |
| KDELC1P1 | hsa-miR-302abcde |
| KDELC1P1 | hsa-miR-372 |
| KDELC1P1 | hsa-miR-373 |
| KDELC1P1 | hsa-miR-428 |
| KDELC1P1 | hsa-miR-519a |
| KDELC1P1 | hsa-miR-520be |
| KDELC1P1 | hsa-miR-520acd-3p |
| KDELC1P1 | hsa-miR-1378 |
| KDELC1P1 | hsa-miR-1420ac |
| KDELC1P1 | hsa-miR-141 |
| KDELC1P1 | hsa-miR-200a |
| KDELC1P1 | hsa-miR-142-3p |
| KDELC1P1 | hsa-miR-145 |
| KDELC1P1 | hsa-miR-15abc |
| KDELC1P1 | hsa-miR-16 |
| KDELC1P1 | hsa-miR-16abc |
| KDELC1P1 | hsa-miR-195 |
| KDELC1P1 | hsa-miR-322 |
| KDELC1P1 | hsa-miR-424 |
| KDELC1P1 | hsa-miR-497 |
| KDELC1P1 | hsa-miR-1907 |
| KDELC1P1 | hsa-miR-183 |
| KDELC1P1 | hsa-miR-18ab |
| KDELC1P1 | hsa-miR-4735-3p |
| KDELC1P1 | hsa-miR-192 |
| KDELC1P1 | hsa-miR-215 |
| KDELC1P1 | hsa-miR-196abc |
| KDELC1P1 | hsa-miR-1ab |
| KDELC1P1 | hsa-miR-206 |
| KDELC1P1 | hsa-miR-613 |
| KDELC1P1 | hsa-miR-200bc |
| KDELC1P1 | hsa-miR-429 |
| KDELC1P1 | hsa-miR-548a |
| KDELC1P1 | hsa-miR-203 |
| KDELC1P1 | hsa-miR-217 |
| KDELC1P1 | hsa-miR-22 |
| KDELC1P1 | hsa-miR-22-3p |
| KDELC1P1 | hsa-miR-31 |
| KDELC1P1 | hsa-miR-425 |
| KDELC1P1 | hsa-miR-425-5p |
| KDELC1P1 | hsa-miR-489 |
| KDELC1P1 | hsa-miR-125a-5p |
| KDELC1P1 | hsa-miR-125b-5p |
| KDELC1P1 | hsa-miR-351 |
| KDELC1P1 | hsa-miR-670 |
| KDELC1P1 | hsa-miR-4319 |
| KDELC1P1 | hsa-miR-455-5p |
| KDELC1P1 | hsa-miR-129-5p |
| KDELC1P1 | hsa-miR-129ab-5p |
| KDELC1P1 | hsa-miR-490-3p |
| AACSP1 | hsa-miR-503 |
| AACSP1 | hsa-miR-130ac |
| AACSP1 | hsa-miR-301ab |
| AACSP1 | hsa-miR-301b |
| AACSP1 | hsa-miR-301b-3p |
| AACSP1 | hsa-miR-454 |
| AACSP1 | hsa-miR-721 |
| AACSP1 | hsa-miR-4295 |
| AACSP1 | hsa-miR-3666 |
| AACSP1 | hsa-miR-7 |
| AACSP1 | hsa-miR-7ab |
| AACSP1 | hsa-miR-96 |
| AACSP1 | hsa-miR-507 |
| AACSP1 | hsa-miR-1271 |
| AACSP1 | hsa-miR-135ab |
| AACSP1 | hsa-miR-135a-5p |
| AACSP1 | hsa-miR-137 |
| AACSP1 | hsa-miR-137ab |
| AACSP1 | hsa-miR-140 |
| AACSP1 | hsa-miR-140-5p |
| AACSP1 | hsa-miR-876-3p |
| AACSP1 | hsa-miR-1244 |
| AACSP1 | hsa-miR-141 |
| AACSP1 | hsa-miR-200a |
| AACSP1 | hsa-miR-143 |
| AACSP1 | hsa-miR-1721 |
| AACSP1 | hsa-miR-4770 |
| AACSP1 | hsa-miR-144 |
| AACSP1 | hsa-miR-15abc |
| AACSP1 | hsa-miR-16 |
| AACSP1 | hsa-miR-16abc |
| AACSP1 | hsa-miR-195 |
| AACSP1 | hsa-miR-322 |
| AACSP1 | hsa-miR-424 |
| AACSP1 | hsa-miR-497 |
| AACSP1 | hsa-miR-1907 |
| AACSP1 | hsa-miR-181abcd |
| AACSP1 | hsa-miR-4262 |
| AACSP1 | hsa-miR-182 |
| AACSP1 | hsa-miR-187 |
| AACSP1 | hsa-miR-199ab-5p |
| AACSP1 | hsa-miR-21 |
| AACSP1 | hsa-miR-590-5p |
| AACSP1 | hsa-miR-214 |
| AACSP1 | hsa-miR-761 |
| AACSP1 | hsa-miR-3619-5p |
| AACSP1 | hsa-miR-22 |
| AACSP1 | hsa-miR-22-3p |
| AACSP1 | hsa-miR-221 |
| AACSP1 | hsa-miR-222 |
| AACSP1 | hsa-miR-222ab |
| AACSP1 | hsa-miR-1928 |
| AACSP1 | hsa-miR-122 |
| AACSP1 | hsa-miR-122a |
| AACSP1 | hsa-miR-1352 |
| AACSP1 | hsa-miR-24 |
| AACSP1 | hsa-miR-24ab |
| AACSP1 | hsa-miR-24-3p |
| AACSP1 | hsa-miR-26ab |
| AACSP1 | hsa-miR-1297 |
| AACSP1 | hsa-miR-4465 |
| AACSP1 | hsa-miR-27abc |
| AACSP1 | hsa-miR-27a-3p |
| AACSP1 | hsa-miR-101 |
| AACSP1 | hsa-miR-101ab |
| AACSP1 | hsa-miR-29abcd |
| AACSP1 | hsa-miR-31 |
| AACSP1 | hsa-miR-103a |
| AACSP1 | hsa-miR-107 |
| AACSP1 | hsa-miR-107ab |
| AACSP1 | hsa-miR-124 |
| AACSP1 | hsa-miR-124ab |
| AACSP1 | hsa-miR-506 |
| AACSP1 | hsa-miR-338 |
| AACSP1 | hsa-miR-338-3p |
| AACSP1 | hsa-miR-125a-5p |
| AACSP1 | hsa-miR-125b-5p |
| AACSP1 | hsa-miR-351 |
| AACSP1 | hsa-miR-670 |
| AACSP1 | hsa-miR-4319 |
| AACSP1 | hsa-miR-128 |
| AACSP1 | hsa-miR-128ab |
| AACSP1 | hsa-miR-129-5p |
| AACSP1 | hsa-miR-129ab-5p |
| CNOT10-AS1 | hsa-miR-34ac |
| CNOT10-AS1 | hsa-miR-34bc-5p |
| CNOT10-AS1 | hsa-miR-449abc |
| CNOT10-AS1 | hsa-miR-449c-5p |
| GTF2F2P1 | hsa-miR-7 |
| GTF2F2P1 | hsa-miR-7ab |
| GTF2F2P1 | hsa-miR-145 |
| GTF2F2P1 | hsa-miR-181abcd |
| GTF2F2P1 | hsa-miR-4262 |
| GTF2F2P1 | hsa-miR-182 |
| GTF2F2P1 | hsa-miR-21 |
| GTF2F2P1 | hsa-miR-590-5p |
| GTF2F2P1 | hsa-miR-216a |
| GTF2F2P1 | hsa-miR-216b |
| GTF2F2P1 | hsa-miR-216b-5p |
| GTF2F2P1 | hsa-miR-23abc |
| GTF2F2P1 | hsa-miR-23b-3p |
| GTF2F2P1 | hsa-miR-34ac |
| GTF2F2P1 | hsa-miR-34bc-5p |
| GTF2F2P1 | hsa-miR-449abc |
| GTF2F2P1 | hsa-miR-449c-5p |
| GTF2F2P1 | hsa-miR-425 |
| GTF2F2P1 | hsa-miR-425-5p |
| GTF2F2P1 | hsa-miR-489 |
| MALAT1 | hsa-miR-503 |
| MALAT1 | hsa-miR-93 |
| MALAT1 | hsa-miR-93a |
| MALAT1 | hsa-miR-105 |
| MALAT1 | hsa-miR-106a |
| MALAT1 | hsa-miR-291a-3p |
| MALAT1 | hsa-miR-294 |
| MALAT1 | hsa-miR-295 |
| MALAT1 | hsa-miR-302abcde |
| MALAT1 | hsa-miR-372 |
| MALAT1 | hsa-miR-373 |
| MALAT1 | hsa-miR-428 |
| MALAT1 | hsa-miR-519a |
| MALAT1 | hsa-miR-520be |
| MALAT1 | hsa-miR-520acd-3p |
| MALAT1 | hsa-miR-1378 |
| MALAT1 | hsa-miR-1420ac |
| MALAT1 | hsa-miR-96 |
| MALAT1 | hsa-miR-507 |
| MALAT1 | hsa-miR-1271 |
| MALAT1 | hsa-miR-135ab |
| MALAT1 | hsa-miR-135a-5p |
| MALAT1 | hsa-miR-138 |
| MALAT1 | hsa-miR-138ab |
| MALAT1 | hsa-miR-140 |
| MALAT1 | hsa-miR-140-5p |
| MALAT1 | hsa-miR-876-3p |
| MALAT1 | hsa-miR-1244 |
| MALAT1 | hsa-miR-141 |
| MALAT1 | hsa-miR-200a |
| MALAT1 | hsa-miR-142-3p |
| MALAT1 | hsa-miR-143 |
| MALAT1 | hsa-miR-1721 |
| MALAT1 | hsa-miR-4770 |
| MALAT1 | hsa-miR-144 |
| MALAT1 | hsa-miR-145 |
| MALAT1 | hsa-miR-146ac |
| MALAT1 | hsa-miR-146b-5p |
| MALAT1 | hsa-miR-148ab-3p |
| MALAT1 | hsa-miR-152 |
| MALAT1 | hsa-miR-150 |
| MALAT1 | hsa-miR-5127 |
| MALAT1 | hsa-miR-155 |
| MALAT1 | hsa-miR-15abc |
| MALAT1 | hsa-miR-16 |
| MALAT1 | hsa-miR-16abc |
| MALAT1 | hsa-miR-195 |
| MALAT1 | hsa-miR-322 |
| MALAT1 | hsa-miR-424 |
| MALAT1 | hsa-miR-497 |
| MALAT1 | hsa-miR-1907 |
| MALAT1 | hsa-miR-17 |
| MALAT1 | hsa-miR-17-5p |
| MALAT1 | hsa-miR-20ab |
| MALAT1 | hsa-miR-20b-5p |
| MALAT1 | hsa-miR-106ab |
| MALAT1 | hsa-miR-427 |
| MALAT1 | hsa-miR-518a-3p |
| MALAT1 | hsa-miR-519d |
| MALAT1 | hsa-miR-181abcd |
| MALAT1 | hsa-miR-4262 |
| MALAT1 | hsa-miR-182 |
| MALAT1 | hsa-miR-192 |
| MALAT1 | hsa-miR-215 |
| MALAT1 | hsa-miR-193 |
| MALAT1 | hsa-miR-193b |
| MALAT1 | hsa-miR-193a-3p |
| MALAT1 | hsa-miR-194 |
| MALAT1 | hsa-miR-1ab |
| MALAT1 | hsa-miR-206 |
| MALAT1 | hsa-miR-613 |
| MALAT1 | hsa-miR-200bc |
| MALAT1 | hsa-miR-429 |
| MALAT1 | hsa-miR-548a |
| MALAT1 | hsa-miR-203 |
| MALAT1 | hsa-miR-204 |
| MALAT1 | hsa-miR-204b |
| MALAT1 | hsa-miR-211 |
| MALAT1 | hsa-miR-205 |
| MALAT1 | hsa-miR-205ab |
| MALAT1 | hsa-miR-208ab |
| MALAT1 | hsa-miR-208ab-3p |
| MALAT1 | hsa-miR-21 |
| MALAT1 | hsa-miR-590-5p |
| MALAT1 | hsa-miR-214 |
| MALAT1 | hsa-miR-761 |
| MALAT1 | hsa-miR-3619-5p |
| MALAT1 | hsa-miR-216a |
| MALAT1 | hsa-miR-216b |
| MALAT1 | hsa-miR-216b-5p |
| MALAT1 | hsa-miR-217 |
| MALAT1 | hsa-miR-218 |
| MALAT1 | hsa-miR-218a |
| MALAT1 | hsa-miR-22 |
| MALAT1 | hsa-miR-22-3p |
| MALAT1 | hsa-miR-23abc |
| MALAT1 | hsa-miR-23b-3p |
| MALAT1 | hsa-miR-24 |
| MALAT1 | hsa-miR-24ab |
| MALAT1 | hsa-miR-24-3p |
| MALAT1 | hsa-miR-25 |
| MALAT1 | hsa-miR-32 |
| MALAT1 | hsa-miR-92abc |
| MALAT1 | hsa-miR-363 |
| MALAT1 | hsa-miR-363-3p |
| MALAT1 | hsa-miR-367 |
| MALAT1 | hsa-miR-26ab |
| MALAT1 | hsa-miR-1297 |
| MALAT1 | hsa-miR-4465 |
| MALAT1 | hsa-miR-101 |
| MALAT1 | hsa-miR-101ab |
| MALAT1 | hsa-miR-29abcd |
| MALAT1 | hsa-miR-30abcdef |
| MALAT1 | hsa-miR-30abe-5p |
| MALAT1 | hsa-miR-384-5p |
| MALAT1 | hsa-miR-124 |
| MALAT1 | hsa-miR-124ab |
| MALAT1 | hsa-miR-506 |
| MALAT1 | hsa-miR-338 |
| MALAT1 | hsa-miR-338-3p |
| MALAT1 | hsa-miR-33ab |
| MALAT1 | hsa-miR-33-5p |
| MALAT1 | hsa-miR-34ac |
| MALAT1 | hsa-miR-34bc-5p |
| MALAT1 | hsa-miR-449abc |
| MALAT1 | hsa-miR-449c-5p |
| MALAT1 | hsa-miR-375 |
| MALAT1 | hsa-miR-383 |
| MALAT1 | hsa-miR-425 |
| MALAT1 | hsa-miR-425-5p |
| MALAT1 | hsa-miR-489 |
| MALAT1 | hsa-miR-125a-5p |
| MALAT1 | hsa-miR-125b-5p |
| MALAT1 | hsa-miR-351 |
| MALAT1 | hsa-miR-670 |
| MALAT1 | hsa-miR-4319 |
| MALAT1 | hsa-miR-455-5p |
| MALAT1 | hsa-miR-129-5p |
| MALAT1 | hsa-miR-129ab-5p |
| MALAT1 | hsa-miR-490-3p |
| MALAT1 | hsa-miR-499-5p |
| ERHP1 | hsa-miR-23abc |
| ERHP1 | hsa-miR-23b-3p |
| ERHP1 | hsa-miR-27abc |
| ERHP1 | hsa-miR-27a-3p |
| SCARNA9 | hsa-miR-210 |
| SCARNA9 | hsa-miR-216a |
| MT1JP | hsa-miR-141 |
| MT1JP | hsa-miR-200a |
| MT1JP | hsa-miR-143 |
| MT1JP | hsa-miR-1721 |
| MT1JP | hsa-miR-4770 |
| MT1JP | hsa-miR-15abc |
| MT1JP | hsa-miR-16 |
| MT1JP | hsa-miR-16abc |
| MT1JP | hsa-miR-195 |
| MT1JP | hsa-miR-322 |
| MT1JP | hsa-miR-424 |
| MT1JP | hsa-miR-497 |
| MT1JP | hsa-miR-1907 |
| MT1JP | hsa-miR-184 |
| MT1JP | hsa-miR-199ab-5p |
| MT1JP | hsa-miR-204 |
| MT1JP | hsa-miR-204b |
| MT1JP | hsa-miR-211 |
| MT1JP | hsa-miR-214 |
| MT1JP | hsa-miR-761 |
| MT1JP | hsa-miR-3619-5p |
| MT1JP | hsa-miR-24 |
| MT1JP | hsa-miR-24ab |
| MT1JP | hsa-miR-24-3p |
| MT1JP | hsa-miR-26ab |
| MT1JP | hsa-miR-1297 |
| MT1JP | hsa-miR-4465 |
| MT1JP | hsa-miR-103a |
| MT1JP | hsa-miR-107 |
| MT1JP | hsa-miR-107ab |
| MT1JP | hsa-miR-34ac |
| MT1JP | hsa-miR-34bc-5p |
| MT1JP | hsa-miR-449abc |
| MT1JP | hsa-miR-449c-5p |
| ARAP1-AS1 | hsa-miR-139-5p |
| ARAP1-AS1 | hsa-miR-145 |
| ARAP1-AS1 | hsa-miR-150 |
| ARAP1-AS1 | hsa-miR-5127 |
| ARAP1-AS1 | hsa-miR-18ab |
| ARAP1-AS1 | hsa-miR-4735-3p |
| LINC00507 | hsa-miR-133abc |
| LINC00507 | hsa-miR-93 |
| LINC00507 | hsa-miR-93a |
| LINC00507 | hsa-miR-105 |
| LINC00507 | hsa-miR-106a |
| LINC00507 | hsa-miR-291a-3p |
| LINC00507 | hsa-miR-294 |
| LINC00507 | hsa-miR-295 |
| LINC00507 | hsa-miR-302abcde |
| LINC00507 | hsa-miR-372 |
| LINC00507 | hsa-miR-373 |
| LINC00507 | hsa-miR-428 |
| LINC00507 | hsa-miR-519a |
| LINC00507 | hsa-miR-520be |
| LINC00507 | hsa-miR-520acd-3p |
| LINC00507 | hsa-miR-1378 |
| LINC00507 | hsa-miR-1420ac |
| LINC00507 | hsa-miR-138 |
| LINC00507 | hsa-miR-138ab |
| LINC00507 | hsa-miR-146ac |
| LINC00507 | hsa-miR-146b-5p |
| LINC00507 | hsa-miR-181abcd |
| LINC00507 | hsa-miR-4262 |
| LINC00507 | hsa-miR-183 |
| LINC00507 | hsa-miR-187 |
| LINC00507 | hsa-miR-18ab |
| LINC00507 | hsa-miR-4735-3p |
| LINC00507 | hsa-miR-194 |
| LINC00507 | hsa-miR-199ab-5p |
| LINC00507 | hsa-miR-19ab |
| LINC00507 | hsa-miR-216b |
| LINC00507 | hsa-miR-216b-5p |
| LINC00507 | hsa-miR-23abc |
| LINC00507 | hsa-miR-23b-3p |
| LINC00507 | hsa-miR-24 |
| LINC00507 | hsa-miR-24ab |
| LINC00507 | hsa-miR-24-3p |
| LINC00507 | hsa-miR-27abc |
| LINC00507 | hsa-miR-27a-3p |
| LINC00507 | hsa-miR-30abcdef |
| LINC00507 | hsa-miR-30abe-5p |
| LINC00507 | hsa-miR-384-5p |
| LINC00507 | hsa-miR-31 |
| LINC00507 | hsa-miR-103a |
| LINC00507 | hsa-miR-107 |
| LINC00507 | hsa-miR-107ab |
| LINC00507 | hsa-miR-375 |
| LINC00507 | hsa-miR-455-5p |
| LINC00507 | hsa-miR-128 |
| LINC00507 | hsa-miR-128ab |
| KCNQ1OT1 | hsa-miR-503 |
| KCNQ1OT1 | hsa-miR-551a |
| KCNQ1OT1 | hsa-miR-130ac |
| KCNQ1OT1 | hsa-miR-301ab |
| KCNQ1OT1 | hsa-miR-301b |
| KCNQ1OT1 | hsa-miR-301b-3p |
| KCNQ1OT1 | hsa-miR-454 |
| KCNQ1OT1 | hsa-miR-721 |
| KCNQ1OT1 | hsa-miR-4295 |
| KCNQ1OT1 | hsa-miR-3666 |
| KCNQ1OT1 | hsa-miR-132 |
| KCNQ1OT1 | hsa-miR-212 |
| KCNQ1OT1 | hsa-miR-212-3p |
| KCNQ1OT1 | hsa-miR-7 |
| KCNQ1OT1 | hsa-miR-7ab |
| KCNQ1OT1 | hsa-miR-133abc |
| KCNQ1OT1 | hsa-miR-9 |
| KCNQ1OT1 | hsa-miR-9ab |
| KCNQ1OT1 | hsa-miR-93 |
| KCNQ1OT1 | hsa-miR-93a |
| KCNQ1OT1 | hsa-miR-105 |
| KCNQ1OT1 | hsa-miR-106a |
| KCNQ1OT1 | hsa-miR-291a-3p |
| KCNQ1OT1 | hsa-miR-294 |
| KCNQ1OT1 | hsa-miR-295 |
| KCNQ1OT1 | hsa-miR-302abcde |
| KCNQ1OT1 | hsa-miR-372 |
| KCNQ1OT1 | hsa-miR-373 |
| KCNQ1OT1 | hsa-miR-428 |
| KCNQ1OT1 | hsa-miR-519a |
| KCNQ1OT1 | hsa-miR-520be |
| KCNQ1OT1 | hsa-miR-520acd-3p |
| KCNQ1OT1 | hsa-miR-1378 |
| KCNQ1OT1 | hsa-miR-1420ac |
| KCNQ1OT1 | hsa-miR-96 |
| KCNQ1OT1 | hsa-miR-507 |
| KCNQ1OT1 | hsa-miR-1271 |
| KCNQ1OT1 | hsa-miR-135ab |
| KCNQ1OT1 | hsa-miR-135a-5p |
| KCNQ1OT1 | hsa-miR-137 |
| KCNQ1OT1 | hsa-miR-137ab |
| KCNQ1OT1 | hsa-miR-138 |
| KCNQ1OT1 | hsa-miR-138ab |
| KCNQ1OT1 | hsa-miR-140 |
| KCNQ1OT1 | hsa-miR-140-5p |
| KCNQ1OT1 | hsa-miR-876-3p |
| KCNQ1OT1 | hsa-miR-1244 |
| KCNQ1OT1 | hsa-miR-141 |
| KCNQ1OT1 | hsa-miR-200a |
| KCNQ1OT1 | hsa-miR-142-3p |
| KCNQ1OT1 | hsa-miR-143 |
| KCNQ1OT1 | hsa-miR-1721 |
| KCNQ1OT1 | hsa-miR-4770 |
| KCNQ1OT1 | hsa-miR-145 |
| KCNQ1OT1 | hsa-miR-146ac |
| KCNQ1OT1 | hsa-miR-146b-5p |
| KCNQ1OT1 | hsa-miR-148ab-3p |
| KCNQ1OT1 | hsa-miR-152 |
| KCNQ1OT1 | hsa-miR-150 |
| KCNQ1OT1 | hsa-miR-5127 |
| KCNQ1OT1 | hsa-miR-153 |
| KCNQ1OT1 | hsa-miR-155 |
| KCNQ1OT1 | hsa-miR-15abc |
| KCNQ1OT1 | hsa-miR-16 |
| KCNQ1OT1 | hsa-miR-16abc |
| KCNQ1OT1 | hsa-miR-195 |
| KCNQ1OT1 | hsa-miR-322 |
| KCNQ1OT1 | hsa-miR-424 |
| KCNQ1OT1 | hsa-miR-497 |
| KCNQ1OT1 | hsa-miR-1907 |
| KCNQ1OT1 | hsa-miR-17 |
| KCNQ1OT1 | hsa-miR-17-5p |
| KCNQ1OT1 | hsa-miR-20ab |
| KCNQ1OT1 | hsa-miR-20b-5p |
| KCNQ1OT1 | hsa-miR-106ab |
| KCNQ1OT1 | hsa-miR-427 |
| KCNQ1OT1 | hsa-miR-518a-3p |
| KCNQ1OT1 | hsa-miR-519d |
| KCNQ1OT1 | hsa-miR-181abcd |
| KCNQ1OT1 | hsa-miR-4262 |
| KCNQ1OT1 | hsa-miR-182 |
| KCNQ1OT1 | hsa-miR-183 |
| KCNQ1OT1 | hsa-miR-let-7 |
| KCNQ1OT1 | hsa-miR-98 |
| KCNQ1OT1 | hsa-miR-4458 |
| KCNQ1OT1 | hsa-miR-4500 |
| KCNQ1OT1 | hsa-miR-187 |
| KCNQ1OT1 | hsa-miR-18ab |
| KCNQ1OT1 | hsa-miR-4735-3p |
| KCNQ1OT1 | hsa-miR-190 |
| KCNQ1OT1 | hsa-miR-190ab |
| KCNQ1OT1 | hsa-miR-192 |
| KCNQ1OT1 | hsa-miR-215 |
| KCNQ1OT1 | hsa-miR-193 |
| KCNQ1OT1 | hsa-miR-193b |
| KCNQ1OT1 | hsa-miR-193a-3p |
| KCNQ1OT1 | hsa-miR-194 |
| KCNQ1OT1 | hsa-miR-196abc |
| KCNQ1OT1 | hsa-miR-199ab-5p |
| KCNQ1OT1 | hsa-miR-19ab |
| KCNQ1OT1 | hsa-miR-1ab |
| KCNQ1OT1 | hsa-miR-206 |
| KCNQ1OT1 | hsa-miR-613 |
| KCNQ1OT1 | hsa-miR-200bc |
| KCNQ1OT1 | hsa-miR-429 |
| KCNQ1OT1 | hsa-miR-548a |
| KCNQ1OT1 | hsa-miR-203 |
| KCNQ1OT1 | hsa-miR-204 |
| KCNQ1OT1 | hsa-miR-204b |
| KCNQ1OT1 | hsa-miR-211 |
| KCNQ1OT1 | hsa-miR-205 |
| KCNQ1OT1 | hsa-miR-205ab |
| KCNQ1OT1 | hsa-miR-208ab |
| KCNQ1OT1 | hsa-miR-208ab-3p |
| KCNQ1OT1 | hsa-miR-214 |
| KCNQ1OT1 | hsa-miR-761 |
| KCNQ1OT1 | hsa-miR-3619-5p |
| KCNQ1OT1 | hsa-miR-216a |
| KCNQ1OT1 | hsa-miR-216b |
| KCNQ1OT1 | hsa-miR-216b-5p |
| KCNQ1OT1 | hsa-miR-217 |
| KCNQ1OT1 | hsa-miR-218 |
| KCNQ1OT1 | hsa-miR-218a |
| KCNQ1OT1 | hsa-miR-219-5p |
| KCNQ1OT1 | hsa-miR-508 |
| KCNQ1OT1 | hsa-miR-508-3p |
| KCNQ1OT1 | hsa-miR-4782-3p |
| KCNQ1OT1 | hsa-miR-22 |
| KCNQ1OT1 | hsa-miR-22-3p |
| KCNQ1OT1 | hsa-miR-221 |
| KCNQ1OT1 | hsa-miR-222 |
| KCNQ1OT1 | hsa-miR-222ab |
| KCNQ1OT1 | hsa-miR-1928 |
| KCNQ1OT1 | hsa-miR-223 |
| KCNQ1OT1 | hsa-miR-122 |
| KCNQ1OT1 | hsa-miR-122a |
| KCNQ1OT1 | hsa-miR-1352 |
| KCNQ1OT1 | hsa-miR-23abc |
| KCNQ1OT1 | hsa-miR-23b-3p |
| KCNQ1OT1 | hsa-miR-24 |
| KCNQ1OT1 | hsa-miR-24ab |
| KCNQ1OT1 | hsa-miR-24-3p |
| KCNQ1OT1 | hsa-miR-25 |
| KCNQ1OT1 | hsa-miR-32 |
| KCNQ1OT1 | hsa-miR-92abc |
| KCNQ1OT1 | hsa-miR-363 |
| KCNQ1OT1 | hsa-miR-363-3p |
| KCNQ1OT1 | hsa-miR-367 |
| KCNQ1OT1 | hsa-miR-26ab |
| KCNQ1OT1 | hsa-miR-1297 |
| KCNQ1OT1 | hsa-miR-4465 |
| KCNQ1OT1 | hsa-miR-27abc |
| KCNQ1OT1 | hsa-miR-27a-3p |
| KCNQ1OT1 | hsa-miR-29abcd |
| KCNQ1OT1 | hsa-miR-30abcdef |
| KCNQ1OT1 | hsa-miR-30abe-5p |
| KCNQ1OT1 | hsa-miR-384-5p |
| KCNQ1OT1 | hsa-miR-103a |
| KCNQ1OT1 | hsa-miR-107 |
| KCNQ1OT1 | hsa-miR-107ab |
| KCNQ1OT1 | hsa-miR-124 |
| KCNQ1OT1 | hsa-miR-124ab |
| KCNQ1OT1 | hsa-miR-506 |
| KCNQ1OT1 | hsa-miR-338 |
| KCNQ1OT1 | hsa-miR-338-3p |
| KCNQ1OT1 | hsa-miR-33a-3p |
| KCNQ1OT1 | hsa-miR-365 |
| KCNQ1OT1 | hsa-miR-365-3p |
| KCNQ1OT1 | hsa-miR-33ab |
| KCNQ1OT1 | hsa-miR-33-5p |
| KCNQ1OT1 | hsa-miR-34ac |
| KCNQ1OT1 | hsa-miR-34bc-5p |
| KCNQ1OT1 | hsa-miR-449abc |
| KCNQ1OT1 | hsa-miR-449c-5p |
| KCNQ1OT1 | hsa-miR-375 |
| KCNQ1OT1 | hsa-miR-425 |
| KCNQ1OT1 | hsa-miR-425-5p |
| KCNQ1OT1 | hsa-miR-489 |
| KCNQ1OT1 | hsa-miR-125a-5p |
| KCNQ1OT1 | hsa-miR-125b-5p |
| KCNQ1OT1 | hsa-miR-351 |
| KCNQ1OT1 | hsa-miR-670 |
| KCNQ1OT1 | hsa-miR-4319 |
| KCNQ1OT1 | hsa-miR-10abc |
| KCNQ1OT1 | hsa-miR-10a-5p |
| KCNQ1OT1 | hsa-miR-455-5p |
| KCNQ1OT1 | hsa-miR-128 |
| KCNQ1OT1 | hsa-miR-128ab |
| KCNQ1OT1 | hsa-miR-129-5p |
| KCNQ1OT1 | hsa-miR-129ab-5p |
| KCNQ1OT1 | hsa-miR-490-3p |
| KCNQ1OT1 | hsa-miR-499-5p |

**Table S4** **8 out of top 200 de-circRNAs have corresponding miRNAs binding.**

| circRNA | miRNA |
| --- | --- |
| hsa_circ_0001495 | hsa-miR-103a-3p |
| hsa_circ_0001495 | hsa-miR-106a-5p |
| hsa_circ_0001495 | hsa-miR-106b-5p |
| hsa_circ_0001495 | hsa-miR-107 |
| hsa_circ_0001459 | hsa-miR-1193 |
| hsa_circ_0001495 | hsa-miR-1252-5p |
| hsa_circ_0000367 | hsa-miR-128-3p |
| hsa_circ_0001459 | hsa-miR-1287-5p |
| hsa_circ_0001543 | hsa-miR-1298-5p |
| hsa_circ_0001495 | hsa-miR-130a-3p |
| hsa_circ_0001495 | hsa-miR-130b-3p |
| hsa_circ_0001543 | hsa-miR-136-5p |
| hsa_circ_0001395 | hsa-miR-141-3p |
| hsa_circ_0000922 | hsa-miR-142-3p |
| hsa_circ_0001495 | hsa-miR-17-5p |
| hsa_circ_0001395 | hsa-miR-182-5p |
| hsa_circ_0001543 | hsa-miR-18a-5p |
| hsa_circ_0001543 | hsa-miR-18b-5p |
| hsa_circ_0001459 | hsa-miR-190a-5p |
| hsa_circ_0001459 | hsa-miR-190b |
| hsa_circ_0000922 | hsa-miR-199a-3p |
| hsa_circ_0000922 | hsa-miR-199b-3p |
| hsa_circ_0001495 | hsa-miR-19a-3p |
| hsa_circ_0001495 | hsa-miR-19b-3p |
| hsa_circ_0001395 | hsa-miR-200a-3p |
| hsa_circ_0001495 | hsa-miR-20a-5p |
| hsa_circ_0001495 | hsa-miR-20b-5p |
| hsa_circ_0001543 | hsa-miR-2116-3p |
| hsa_circ_0000367 | hsa-miR-216a-3p |
| hsa_circ_0001495 | hsa-miR-224-5p |
| hsa_circ_0001543 | hsa-miR-2355-5p |
| hsa_circ_0000367 | hsa-miR-25-3p |
| hsa_circ_0000367 | hsa-miR-2681-3p |
| hsa_circ_0001543 | hsa-miR-27a-3p |
| hsa_circ_0001543 | hsa-miR-27b-3p |
| hsa_circ_0001459 | hsa-miR-296-3p |
| hsa_circ_0001020 | hsa-miR-29a-3p |
| hsa_circ_0001020 | hsa-miR-29b-3p |
| hsa_circ_0001020 | hsa-miR-29c-3p |
| hsa_circ_0001495 | hsa-miR-301a-3p |
| hsa_circ_0001495 | hsa-miR-301b-3p |
| hsa_circ_0001543 | hsa-miR-3064-5p |
| hsa_circ_0000922 | hsa-miR-3129-5p |
| hsa_circ_0001543 | hsa-miR-3173-5p |
| hsa_circ_0000367 | hsa-miR-32-5p |
| hsa_circ_0001543 | hsa-miR-323a-3p |
| hsa_circ_0001543 | hsa-miR-328-3p |
| hsa_circ_0001495 | hsa-miR-34a-5p |
| hsa_circ_0001543 | hsa-miR-34a-5p |
| hsa_circ_0001495 | hsa-miR-34c-5p |
| hsa_circ_0001543 | hsa-miR-34c-5p |
| hsa_circ_0000922 | hsa-miR-3612 |
| hsa_circ_0001543 | hsa-miR-3614-5p |
| hsa_circ_0000367 | hsa-miR-363-3p |
| hsa_circ_0001495 | hsa-miR-3666 |
| hsa_circ_0000367 | hsa-miR-367-3p |
| hsa_circ_0000367 | hsa-miR-3681-3p |
| hsa_circ_0000367 | hsa-miR-3681-5p |
| hsa_circ_0001495 | hsa-miR-370-3p |
| hsa_circ_0001543 | hsa-miR-370-3p |
| hsa_circ_0001459 | hsa-miR-378a-3p |
| hsa_circ_0001459 | hsa-miR-378b |
| hsa_circ_0001459 | hsa-miR-378c |
| hsa_circ_0001459 | hsa-miR-378d |
| hsa_circ_0001459 | hsa-miR-378e |
| hsa_circ_0001459 | hsa-miR-378f |
| hsa_circ_0001459 | hsa-miR-378h |
| hsa_circ_0001459 | hsa-miR-378i |
| hsa_circ_0001543 | hsa-miR-382-5p |
| hsa_circ_0001543 | hsa-miR-421 |
| hsa_circ_0001459 | hsa-miR-422a |
| hsa_circ_0001495 | hsa-miR-4295 |
| hsa_circ_0001543 | hsa-miR-432-5p |
| hsa_circ_0001543 | hsa-miR-4424 |
| hsa_circ_0001495 | hsa-miR-449a |
| hsa_circ_0001543 | hsa-miR-449a |
| hsa_circ_0001495 | hsa-miR-449b-5p |
| hsa_circ_0001543 | hsa-miR-449b-5p |
| hsa_circ_0001543 | hsa-miR-450b-5p |
| hsa_circ_0001495 | hsa-miR-454-3p |
| hsa_circ_0001543 | hsa-miR-4731-5p |
| hsa_circ_0001543 | hsa-miR-4735-3p |
| hsa_circ_0001020 | hsa-miR-496 |
| hsa_circ_0001459 | hsa-miR-496 |
| hsa_circ_0001459 | hsa-miR-5047 |
| hsa_circ_0001459 | hsa-miR-508-3p |
| hsa_circ_0001543 | hsa-miR-5094 |
| hsa_circ_0001495 | hsa-miR-515-5p |
| hsa_circ_0001543 | hsa-miR-515-5p |
| hsa_circ_0001495 | hsa-miR-516b-5p |
| hsa_circ_0001495 | hsa-miR-519a-3p |
| hsa_circ_0001495 | hsa-miR-519b-3p |
| hsa_circ_0001495 | hsa-miR-519c-3p |
| hsa_circ_0001495 | hsa-miR-519d-3p |
| hsa_circ_0001495 | hsa-miR-519e-5p |
| hsa_circ_0001543 | hsa-miR-519e-5p |
| hsa_circ_0000367 | hsa-miR-520a-5p |
| hsa_circ_0000467 | hsa-miR-520g-3p |
| hsa_circ_0000467 | hsa-miR-520h |
| hsa_circ_0000367 | hsa-miR-525-5p |
| hsa_circ_0001495 | hsa-miR-526b-3p |
| hsa_circ_0001020 | hsa-miR-526b-5p |
| hsa_circ_0001543 | hsa-miR-545-5p |
| hsa_circ_0001495 | hsa-miR-551a |
| hsa_circ_0001495 | hsa-miR-551b-3p |
| hsa_circ_0001543 | hsa-miR-556-3p |
| hsa_circ_0000467 | hsa-miR-576-5p |
| hsa_circ_0001543 | hsa-miR-629-5p |
| hsa_circ_0000922 | hsa-miR-650 |
| hsa_circ_0001543 | hsa-miR-6504-5p |
| hsa_circ_0001543 | hsa-miR-665 |
| hsa_circ_0001543 | hsa-miR-6799-3p |
| hsa_circ_0000367 | hsa-miR-6849-5p |
| hsa_circ_0001495 | hsa-miR-6893-3p |
| hsa_circ_0001543 | hsa-miR-6893-3p |
| hsa_circ_0001495 | hsa-miR-766-5p |
| hsa_circ_0000467 | hsa-miR-888-5p |
| hsa_circ_0000467 | hsa-miR-892c-5p |
| hsa_circ_0001495 | hsa-miR-9-3p |
| hsa_circ_0001543 | hsa-miR-9-5p |
| hsa_circ_0000367 | hsa-miR-92a-3p |
| hsa_circ_0000367 | hsa-miR-92b-3p |
| hsa_circ_0001495 | hsa-miR-93-5p |
| hsa_circ_0001543 | hsa-miR-942-5p |
| hsa_circ_0001495 | hsa-miR-944 |

**Table S5 Enrichment pathways between high and low risk groups results from GSEA**

| NAME | Brief description | SIZE | NES | NOM p-val | FDR q-val |
| --- | --- | --- | --- | --- | --- |
| HALLMARK_OXIDATIVE_PHOSPHORYLATION | Genes encoding proteins involved in oxidative phosphorylation. | 200 | -2.0865695 | 0 | 0.013448074 |
| HALLMARK_UV_RESPONSE_DN | Genes down-regulated in response to ultraviolet (UV) radiation. | 144 | 2.0679054 | 0 | 0.037007358 |
| HALLMARK_HEDGEHOG_SIGNALING | Genes up-regulated by activation of hedgehog signaling. | 36 | 2.0614717 | 0.004016064 | 0.01891239 |
| HALLMARK_MYOGENESIS | Genes involved in development of skeletal muscle (myogenesis). | 200 | 1.9866164 | 0.007984032 | 0.030244028 |
| HALLMARK_EPITHELIAL_MESENCHYMAL_TRANSITION | Genes defining epithelial-mesenchymal transition, as in wound healing, fibrosis and metastasis. | 200 | 1.9826127 | 0.018518519 | 0.023151752 |
| HALLMARK_ANGIOGENESIS | Genes up-regulated during formation of blood vessels (angiogenesis). | 36 | 1.8981189 | 0.006289308 | 0.0377786 |
| HALLMARK_MITOTIC_SPINDLE | Genes important for mitotic spindle assembly. | 199 | 1.8911692 | 0.02471483 | 0.0330583 |
| HALLMARK_FATTY_ACID_METABOLISM | Genes encoding proteins involved in metabolism of fatty acids. | 158 | -1.8778036 | 0.00209205 | 0.07009872 |
| HALLMARK_APICAL_JUNCTION | Genes encoding components of apical junction complex. | 199 | 1.8289437 | 0.021611001 | 0.04428156 |
