# Supplementary material for: The noncoding RNAs regulating pyroptosis in colon adenocarcinoma were derived from the construction of a ceRNA network and used to develop a prognostic model
Source: BMC Med Genomics. 2022 Sep 20;15:201. doi: 10.1186/s12920-022-01359-w (PMC9490888; doi:10.1186/s12920-022-01359-w)
Supplement: Supplementary file 2 — Additional file 2: Table S6. Accession numbers of COAD from TCGA database. [file 12920_2022_1359_MOESM2_ESM.docx]

Table S6 Accession numbers of COAD from TCGA database.

| Accession number |
| --- |
| TCGA-A6-2671-11A-01R-A32Z-07 |
| TCGA-AA-3655-11A-01R-1723-07 |
| TCGA-AA-3517-11A-01R-A32Z-07 |
| TCGA-AA-3712-11A-01R-1723-07 |
| TCGA-A6-5659-11A-01R-1653-07 |
| TCGA-A6-2682-11A-01R-A32Z-07 |
| TCGA-AZ-6603-11A-02R-1839-07 |
| TCGA-AA-3511-11A-01R-1839-07 |
| TCGA-A6-2683-11A-01R-A32Z-07 |
| TCGA-AA-3520-11A-01R-A32Z-07 |
| TCGA-A6-5665-11A-01R-1653-07 |
| TCGA-A6-5667-11A-01R-1723-07 |
| TCGA-AZ-6601-11A-01R-1774-07 |
| TCGA-AZ-6600-11A-01R-1774-07 |
| TCGA-AA-3525-11A-01R-A32Z-07 |
| TCGA-A6-2684-11A-01R-A32Z-07 |
| TCGA-AA-3522-11A-01R-A32Z-07 |
| TCGA-AZ-6605-11A-01R-1839-07 |
| TCGA-AA-3713-11A-01R-1723-07 |
| TCGA-AZ-6599-11A-01R-1774-07 |
| TCGA-A6-2685-11A-01R-A32Z-07 |
| TCGA-AZ-6598-11A-01R-1774-07 |
| TCGA-AA-3531-11A-01R-A32Z-07 |
| TCGA-AA-3496-11A-01R-1839-07 |
| TCGA-AA-3660-11A-01R-1723-07 |
| TCGA-AA-3534-11A-01R-A32Z-07 |
| TCGA-AA-3527-11A-01R-A32Z-07 |
| TCGA-A6-2675-11A-01R-1723-07 |
| TCGA-A6-2679-11A-01R-A32Z-07 |
| TCGA-A6-2680-11A-01R-A32Z-07 |
| TCGA-A6-2686-11A-01R-A32Z-07 |
| TCGA-AA-3662-11A-01R-1723-07 |
| TCGA-A6-2678-11A-01R-A32Z-07 |
| TCGA-AA-3489-11A-01R-1839-07 |
| TCGA-AA-3663-11A-01R-1723-07 |
| TCGA-AA-3518-11A-01R-1672-07 |
| TCGA-AA-3697-11A-01R-1723-07 |
| TCGA-AA-3514-11A-01R-A32Z-07 |
| TCGA-A6-5662-11A-01R-1653-07 |
| TCGA-4N-A93T-01A-11R-A37K-07 |
| TCGA-AA-3511-01A-21R-1839-07 |
| TCGA-AA-A00K-01A-02R-A002-07 |
| TCGA-G4-6307-01A-11R-1723-07 |
| TCGA-A6-2683-01A-01R-0821-07 |
| TCGA-CM-5864-01A-01R-1653-07 |
| TCGA-G4-6320-01A-11R-1723-07 |
| TCGA-A6-5666-01A-01R-1653-07 |
| TCGA-AA-3662-01A-01R-1723-07 |
| TCGA-AA-3846-01A-01R-1022-07 |
| TCGA-CK-5913-01A-11R-1653-07 |
| TCGA-NH-A6GB-01A-11R-A37K-07 |
| TCGA-AA-3492-01A-01R-1410-07 |
| TCGA-A6-6648-01A-11R-1774-07 |
| TCGA-F4-6854-01A-11R-1928-07 |
| TCGA-A6-2686-01A-01R-A32Z-07 |
| TCGA-AA-3525-01A-02R-0826-07 |
| TCGA-A6-6654-01A-21R-1839-07 |
| TCGA-AA-3864-01A-01R-1022-07 |
| TCGA-CM-6161-01A-11R-1653-07 |
| TCGA-AA-3675-01A-02R-0905-07 |
| TCGA-AA-3506-01A-01R-1410-07 |
| TCGA-AA-3861-01A-01R-1022-07 |
| TCGA-CM-6170-01A-11R-1653-07 |
| TCGA-A6-2681-01A-01R-1410-07 |
| TCGA-AU-3779-01A-01R-1723-07 |
| TCGA-DM-A28E-01A-11R-A32Y-07 |
| TCGA-A6-2676-01A-01R-0826-07 |
| TCGA-F4-6807-01A-11R-1839-07 |
| TCGA-G4-6310-01A-11R-1723-07 |
| TCGA-CM-4752-01A-01R-1410-07 |
| TCGA-AZ-6603-01A-11R-1839-07 |
| TCGA-CM-6167-01A-11R-1653-07 |
| TCGA-AA-3534-01A-01R-0821-07 |
| TCGA-CM-6676-01A-11R-1839-07 |
| TCGA-AA-A02Y-01A-43R-A32Y-07 |
| TCGA-CM-5860-01A-01R-1653-07 |
| TCGA-AY-A71X-01A-12R-A37K-07 |
| TCGA-AZ-6606-01A-11R-1839-07 |
| TCGA-G4-6297-01A-11R-1723-07 |
| TCGA-AA-3526-01A-02R-A32Z-07 |
| TCGA-AA-3845-01A-01R-1022-07 |
| TCGA-AA-3678-01A-01R-0905-07 |
| TCGA-G4-6625-01A-21R-1774-07 |
| TCGA-AD-A5EK-01A-11R-A28H-07 |
| TCGA-CA-5256-01A-01R-1410-07 |
| TCGA-F4-6808-01A-11R-1839-07 |
| TCGA-A6-5659-01A-01R-A278-07 |
| TCGA-AA-3554-01A-01R-0826-07 |
| TCGA-AA-3667-01A-01R-0905-07 |
| TCGA-CA-5797-01A-01R-1653-07 |
| TCGA-AZ-6601-01A-11R-1774-07 |
| TCGA-G4-6626-01A-11R-1774-07 |
| TCGA-AA-A02E-01A-01R-A00A-07 |
| TCGA-AA-3858-01A-01R-0905-07 |
| TCGA-CM-5341-01A-01R-1410-07 |
| TCGA-AD-6548-01A-11R-1839-07 |
| TCGA-D5-6929-01A-31R-1928-07 |
| TCGA-NH-A8F7-01A-11R-A41B-07 |
| TCGA-AA-3930-01A-01R-1022-07 |
| TCGA-A6-6782-01A-11R-1839-07 |
| TCGA-AM-5821-01A-01R-1653-07 |
| TCGA-AA-3561-01A-01R-0821-07 |
| TCGA-D5-6926-01A-11R-1928-07 |
| TCGA-AA-3538-01A-01R-0821-07 |
| TCGA-AA-3860-01A-02R-0905-07 |
| TCGA-AA-A01I-01A-02R-A089-07 |
| TCGA-AA-3544-01A-01R-1873-07 |
| TCGA-AA-A00R-01A-01R-A002-07 |
| TCGA-AA-3850-01A-01R-1022-07 |
| TCGA-AA-3815-01A-01R-1022-07 |
| TCGA-AA-3663-01A-01R-1723-07 |
| TCGA-QG-A5Z2-01A-11R-A28H-07 |
| TCGA-AA-3986-01A-02R-1022-07 |
| TCGA-CM-5868-01A-01R-1653-07 |
| TCGA-AA-A00L-01A-01R-A002-07 |
| TCGA-A6-6649-01A-11R-1774-07 |
| TCGA-A6-A567-01A-31R-A28H-07 |
| TCGA-F4-6805-01A-11R-1839-07 |
| TCGA-AA-3681-01A-01R-0905-07 |
| TCGA-D5-6533-01A-11R-1723-07 |
| TCGA-A6-2675-01A-02R-1723-07 |
| TCGA-AA-3819-01A-01R-0905-07 |
| TCGA-AA-3552-01A-01R-0821-07 |
| TCGA-AA-A03J-01A-21R-A16W-07 |
| TCGA-CM-5861-01A-01R-1653-07 |
| TCGA-5M-AATA-01A-31R-A41B-07 |
| TCGA-G4-6295-01A-11R-1723-07 |
| TCGA-AA-A017-01A-01R-A00A-07 |
| TCGA-CM-5349-01A-21R-1723-07 |
| TCGA-D5-5538-01A-01R-1653-07 |
| TCGA-CK-6747-01A-11R-1839-07 |
| TCGA-D5-6530-01A-11R-1723-07 |
| TCGA-A6-5664-01A-21R-1839-07 |
| TCGA-AA-3872-01A-01R-1022-07 |
| TCGA-AA-3518-01A-02R-0826-07 |
| TCGA-AZ-5407-01A-01R-1723-07 |
| TCGA-AD-6895-01A-11R-1928-07 |
| TCGA-D5-6538-01A-11R-1723-07 |
| TCGA-AU-6004-01A-11R-1723-07 |
| TCGA-AA-A00E-01A-01R-A002-07 |
| TCGA-AA-A00D-01A-01R-A002-07 |
| TCGA-AA-3812-01A-01R-0905-07 |
| TCGA-A6-2671-01A-01R-1410-07 |
| TCGA-AA-3713-01A-21R-1723-07 |
| TCGA-D5-6531-01A-11R-1723-07 |
| TCGA-AA-3548-01A-01R-1873-07 |
| TCGA-G4-6294-01A-11R-1774-07 |
| TCGA-CM-6172-01A-11R-1653-07 |
| TCGA-AA-3496-01A-21R-1839-07 |
| TCGA-A6-6142-01A-11R-1774-07 |
| TCGA-CM-4747-01A-01R-1410-07 |
| TCGA-AA-A02W-01A-01R-A00A-07 |
| TCGA-AD-6901-01A-11R-1928-07 |
| TCGA-DM-A28A-01A-21R-A32Y-07 |
| TCGA-G4-6304-01A-11R-1928-07 |
| TCGA-AA-3870-01A-01R-1022-07 |
| TCGA-AA-3710-01A-01R-1022-07 |
| TCGA-CM-6677-01A-11R-1839-07 |
| TCGA-G4-6299-01A-11R-1774-07 |
| TCGA-A6-6137-01A-11R-1774-07 |
| TCGA-AA-3697-01A-01R-1723-07 |
| TCGA-CA-6716-01A-11R-1839-07 |
| TCGA-DM-A1D0-01A-11R-A155-07 |
| TCGA-AA-3856-01A-01R-0905-07 |
| TCGA-DM-A0XD-01A-12R-A155-07 |
| TCGA-AZ-6607-01A-11R-1839-07 |
| TCGA-AD-6890-01A-11R-1928-07 |
| TCGA-AA-3489-01A-21R-1839-07 |
| TCGA-3L-AA1B-01A-11R-A37K-07 |
| TCGA-RU-A8FL-01A-11R-A37K-07 |
| TCGA-CK-5912-01A-11R-1653-07 |
| TCGA-A6-5667-01A-21R-1723-07 |
| TCGA-AZ-6605-01A-11R-1839-07 |
| TCGA-AA-3527-01A-01R-0821-07 |
| TCGA-D5-6924-01A-11R-1928-07 |
| TCGA-AA-A01C-01A-01R-A00A-07 |
| TCGA-A6-2678-01A-01R-0821-07 |
| TCGA-G4-6323-01A-11R-1723-07 |
| TCGA-A6-6650-01A-11R-1774-07 |
| TCGA-DM-A28M-01A-12R-A16W-07 |
| TCGA-CM-5344-01A-21R-1723-07 |
| TCGA-AZ-4313-01A-01R-1410-07 |
| TCGA-NH-A5IV-01A-42R-A37K-07 |
| TCGA-AA-3494-01A-01R-1410-07 |
| TCGA-CM-6164-01A-11R-1653-07 |
| TCGA-CM-4743-01A-01R-1723-07 |
| TCGA-QG-A5YX-01A-11R-A28H-07 |
| TCGA-AA-3664-01A-01R-0905-07 |
| TCGA-A6-6653-01A-11R-1774-07 |
| TCGA-AY-A54L-01A-11R-A28H-07 |
| TCGA-CM-6680-01A-11R-1839-07 |
| TCGA-A6-5661-01A-01R-1653-07 |
| TCGA-AA-3685-01A-02R-A32Z-07 |
| TCGA-AA-3530-01A-01R-1022-07 |
| TCGA-A6-5659-01A-01R-1653-07 |
| TCGA-5M-AAT4-01A-11R-A41B-07 |
| TCGA-AM-5820-01A-01R-1653-07 |
| TCGA-F4-6459-01A-11R-1774-07 |
| TCGA-AA-A00A-01A-01R-A002-07 |
| TCGA-AA-3655-01A-02R-1723-07 |
| TCGA-A6-5660-01A-01R-1653-07 |
| TCGA-CM-6674-01A-11R-1839-07 |
| TCGA-CM-6166-01A-11R-1653-07 |
| TCGA-DM-A1D8-01A-11R-A155-07 |
| TCGA-F4-6570-01A-11R-1774-07 |
| TCGA-AA-A01X-01A-21R-A083-07 |
| TCGA-AD-6963-01A-11R-1928-07 |
| TCGA-AA-3939-01A-01R-1022-07 |
| TCGA-T9-A92H-01A-11R-A37K-07 |
| TCGA-A6-5662-01A-01R-1653-07 |
| TCGA-DM-A0XF-01A-11R-A155-07 |
| TCGA-A6-4105-01A-02R-1774-07 |
| TCGA-A6-2672-01A-01R-0826-07 |
| TCGA-A6-2684-01A-01R-A278-07 |
| TCGA-AA-A01F-01A-01R-A002-07 |
| TCGA-D5-6535-01A-11R-1723-07 |
| TCGA-A6-A56B-01A-31R-A28H-07 |
| TCGA-AA-3977-01A-01R-1022-07 |
| TCGA-D5-6536-01A-11R-1723-07 |
| TCGA-D5-6529-01A-11R-1774-07 |
| TCGA-D5-6931-01A-11R-1928-07 |
| TCGA-DM-A1HA-01A-11R-A155-07 |
| TCGA-AA-3955-01A-02R-1022-07 |
| TCGA-G4-6314-01A-11R-1723-07 |
| TCGA-D5-5540-01A-01R-1653-07 |
| TCGA-AA-3971-01A-01R-1022-07 |
| TCGA-G4-6317-02A-11R-2066-07 |
| TCGA-AA-3818-01A-01R-0905-07 |
| TCGA-AA-A01V-01A-23R-A083-07 |
| TCGA-CA-5255-01A-11R-1839-07 |
| TCGA-G4-6627-01A-11R-1774-07 |
| TCGA-AA-3521-01A-01R-0821-07 |
| TCGA-AA-A00O-01A-02R-A089-07 |
| TCGA-AZ-4323-01A-21R-1839-07 |
| TCGA-AA-3524-01A-02R-0821-07 |
| TCGA-AA-3989-01A-01R-1022-07 |
| TCGA-AA-3542-01A-02R-1873-07 |
| TCGA-D5-5541-01A-01R-1653-07 |
| TCGA-AA-A022-01A-21R-A16W-07 |
| TCGA-AA-3666-01A-02R-0905-07 |
| TCGA-DM-A0X9-01A-11R-A155-07 |
| TCGA-AA-A01Z-01A-11R-A083-07 |
| TCGA-AA-3519-01A-02R-0821-07 |
| TCGA-D5-6539-01A-11R-1723-07 |
| TCGA-D5-6920-01A-11R-1928-07 |
| TCGA-CM-6678-01A-11R-1839-07 |
| TCGA-D5-6923-01A-11R-A32Z-07 |
| TCGA-AA-3941-01A-01R-1022-07 |
| TCGA-AD-6888-01A-11R-1928-07 |
| TCGA-AA-3855-01A-01R-1022-07 |
| TCGA-AY-5543-01A-01R-1653-07 |
| TCGA-AA-A00W-01A-01R-A002-07 |
| TCGA-AA-3560-01A-01R-0821-07 |
| TCGA-AZ-6598-01A-11R-1774-07 |
| TCGA-CM-4746-01A-01R-1410-07 |
| TCGA-G4-6321-01A-11R-1723-07 |
| TCGA-AA-3866-01A-01R-1022-07 |
| TCGA-AY-6197-01A-11R-1723-07 |
| TCGA-AA-A01T-01A-21R-A16W-07 |
| TCGA-AZ-6608-01A-11R-1839-07 |
| TCGA-AA-A00Z-01A-01R-A002-07 |
| TCGA-AA-A02J-01A-01R-A00A-07 |
| TCGA-CK-5914-01A-11R-1653-07 |
| TCGA-A6-6651-01A-21R-1839-07 |
| TCGA-DM-A1DA-01A-11R-A155-07 |
| TCGA-CK-5916-01A-11R-1653-07 |
| TCGA-AD-A5EJ-01A-11R-A28H-07 |
| TCGA-AZ-6599-01A-11R-1774-07 |
| TCGA-CA-5254-01A-21R-1839-07 |
| TCGA-A6-4107-01A-02R-1410-07 |
| TCGA-G4-6293-01A-11R-1723-07 |
| TCGA-QG-A5YW-01A-11R-A28H-07 |
| TCGA-AA-3522-01A-01R-0821-07 |
| TCGA-5M-AAT5-01A-21R-A41B-07 |
| TCGA-AA-A029-01A-01R-A00A-07 |
| TCGA-AA-3869-01A-01R-1022-07 |
| TCGA-AY-6386-01A-21R-1723-07 |
| TCGA-A6-2680-01A-01R-1410-07 |
| TCGA-AA-3862-01A-01R-1022-07 |
| TCGA-AA-3984-01A-02R-1022-07 |
| TCGA-AA-3693-01A-01R-0905-07 |
| TCGA-AA-3814-01A-01R-0905-07 |
| TCGA-AA-A00U-01A-01R-A002-07 |
| TCGA-AA-A02H-01A-01R-A089-07 |
| TCGA-G4-6317-01A-11R-1723-07 |
| TCGA-AA-3679-01A-02R-0905-07 |
| TCGA-AA-3562-01A-02R-0821-07 |
| TCGA-CM-4744-01A-01R-A32Z-07 |
| TCGA-NH-A8F8-01A-72R-A41B-07 |
| TCGA-G4-6303-01A-11R-1774-07 |
| TCGA-G4-6586-01A-11R-1774-07 |
| TCGA-CA-6719-01A-11R-1839-07 |
| TCGA-QG-A5YV-01A-11R-A28H-07 |
| TCGA-AA-3495-01A-01R-1410-07 |
| TCGA-AA-3842-01A-01R-1022-07 |
| TCGA-AA-3844-01A-01R-1022-07 |
| TCGA-AA-3982-01A-02R-1022-07 |
| TCGA-A6-6140-01A-11R-1774-07 |
| TCGA-CA-6718-01A-11R-1839-07 |
| TCGA-AA-A01K-01A-01R-A00A-07 |
| TCGA-AA-3509-01A-01R-1410-07 |
| TCGA-A6-5656-01A-21R-1839-07 |
| TCGA-QL-A97D-01A-12R-A41B-07 |
| TCGA-F4-6461-01A-11R-1774-07 |
| TCGA-F4-6855-01A-11R-1928-07 |
| TCGA-D5-6541-01A-11R-1723-07 |
| TCGA-AA-3841-01A-01R-0905-07 |
| TCGA-AA-3696-01A-01R-0905-07 |
| TCGA-AZ-6600-01A-11R-1774-07 |
| TCGA-AA-3520-01A-01R-0821-07 |
| TCGA-G4-6588-01A-11R-1774-07 |
| TCGA-CM-5348-01A-21R-1723-07 |
| TCGA-AA-3875-01A-01R-0905-07 |
| TCGA-AA-3549-01A-02R-0821-07 |
| TCGA-A6-2685-01A-01R-1410-07 |
| TCGA-AA-A02O-01A-21R-A16W-07 |
| TCGA-AA-3510-01A-01R-1410-07 |
| TCGA-CA-6715-01A-21R-1839-07 |
| TCGA-A6-6141-01A-11R-1774-07 |
| TCGA-AA-A02R-01A-01R-A00A-07 |
| TCGA-AY-A8YK-01A-11R-A41B-07 |
| TCGA-A6-6138-01A-11R-1774-07 |
| TCGA-AA-3848-01A-01R-0905-07 |
| TCGA-NH-A8F7-06A-31R-A41B-07 |
| TCGA-D5-6927-01A-21R-1928-07 |
| TCGA-AA-3867-01A-01R-1022-07 |
| TCGA-G4-6306-01A-11R-1774-07 |
| TCGA-AA-A01S-01A-21R-A083-07 |
| TCGA-AA-3514-01A-02R-0821-07 |
| TCGA-A6-3810-01A-01R-A278-07 |
| TCGA-AA-A010-01A-01R-A089-07 |
| TCGA-A6-5656-01A-21R-A278-07 |
| TCGA-A6-6652-01A-11R-1774-07 |
| TCGA-5M-AATE-01A-11R-A41B-07 |
| TCGA-A6-3808-01A-01R-1022-07 |
| TCGA-AD-6964-01A-11R-1928-07 |
| TCGA-AA-3833-01A-01R-0905-07 |
| TCGA-CM-6679-01A-11R-1839-07 |
| TCGA-5M-AAT6-01A-11R-A41B-07 |
| TCGA-AA-3532-01A-01R-0821-07 |
| TCGA-F4-6460-01A-11R-1774-07 |
| TCGA-AA-3531-01A-01R-0821-07 |
| TCGA-A6-3810-01A-01R-1022-07 |
| TCGA-AZ-4616-01A-21R-1839-07 |
| TCGA-DM-A28G-01A-11R-A16W-07 |
| TCGA-AA-3956-01A-02R-1022-07 |
| TCGA-A6-3807-01A-01R-1022-07 |
| TCGA-AA-3688-01A-01R-0905-07 |
| TCGA-CM-6168-01A-11R-1653-07 |
| TCGA-AZ-4614-01A-01R-1410-07 |
| TCGA-DM-A28F-01A-11R-A32Y-07 |
| TCGA-DM-A28C-01A-11R-A32Y-07 |
| TCGA-AA-3673-01A-01R-0905-07 |
| TCGA-AA-3973-01A-01R-1022-07 |
| TCGA-DM-A1DB-01A-11R-A155-07 |
| TCGA-AA-3979-01A-01R-1022-07 |
| TCGA-AY-4070-01A-01R-1113-07 |
| TCGA-AD-6965-01A-11R-1928-07 |
| TCGA-A6-2684-01C-08R-A277-07 |
| TCGA-AA-3680-01A-01R-0905-07 |
| TCGA-AZ-4615-01A-01R-1410-07 |
| TCGA-AA-3952-01A-01R-1022-07 |
| TCGA-AZ-5403-01A-01R-1653-07 |
| TCGA-AZ-4315-01A-01R-1410-07 |
| TCGA-AA-3972-01A-01R-1022-07 |
| TCGA-AA-3517-01A-01R-0821-07 |
| TCGA-AA-3529-01A-02R-0821-07 |
| TCGA-NH-A50T-01A-11R-A28H-07 |
| TCGA-NH-A50V-01A-11R-A28H-07 |
| TCGA-AA-3502-01A-01R-1410-07 |
| TCGA-SS-A7HO-01A-21R-A37K-07 |
| TCGA-AA-A01Q-01A-01R-A002-07 |
| TCGA-F4-6809-01A-11R-1839-07 |
| TCGA-AA-3553-01A-01R-0821-07 |
| TCGA-CK-6746-01A-11R-1839-07 |
| TCGA-G4-6309-01A-21R-1839-07 |
| TCGA-CK-5915-01A-11R-1653-07 |
| TCGA-AD-6889-01A-11R-1928-07 |
| TCGA-G4-6298-01A-11R-1723-07 |
| TCGA-AA-3968-01A-01R-1022-07 |
| TCGA-CM-6675-01A-11R-1839-07 |
| TCGA-AA-3660-01A-01R-1723-07 |
| TCGA-CM-6165-01A-11R-1653-07 |
| TCGA-AY-4071-01A-01R-1113-07 |
| TCGA-AA-3672-01A-01R-0905-07 |
| TCGA-CM-6163-01A-11R-1653-07 |
| TCGA-A6-2677-01A-01R-0821-07 |
| TCGA-AA-3851-01A-01R-1022-07 |
| TCGA-AA-3712-01A-21R-1723-07 |
| TCGA-AZ-4308-01A-01R-1410-07 |
| TCGA-G4-6311-01A-11R-1723-07 |
| TCGA-A6-2682-01A-01R-1410-07 |
| TCGA-A6-6650-01A-11R-A278-07 |
| TCGA-NH-A6GA-01A-11R-A37K-07 |
| TCGA-AY-A69D-01A-11R-A37K-07 |
| TCGA-AA-3975-01A-01R-1022-07 |
| TCGA-A6-A5ZU-01A-11R-A28H-07 |
| TCGA-G4-6628-01A-11R-1839-07 |
| TCGA-CM-5862-01A-01R-1653-07 |
| TCGA-D5-6898-01A-11R-1928-07 |
| TCGA-AA-A00F-01A-01R-A002-07 |
| TCGA-D5-6932-01A-11R-1928-07 |
| TCGA-F4-6806-01A-11R-1839-07 |
| TCGA-F4-6569-01A-11R-1774-07 |
| TCGA-A6-2679-01A-02R-1410-07 |
| TCGA-A6-5657-01A-01R-A32Z-07 |
| TCGA-AA-A00Q-01A-01R-A002-07 |
| TCGA-CM-6169-01A-11R-1653-07 |
| TCGA-AA-3980-01A-02R-1022-07 |
| TCGA-A6-5665-01A-01R-1653-07 |
| TCGA-DM-A28H-01A-11R-A16W-07 |
| TCGA-F4-6703-01A-11R-1839-07 |
| TCGA-D5-6922-01A-11R-1928-07 |
| TCGA-D5-5537-01A-21R-1928-07 |
| TCGA-DM-A1D4-01A-21R-A155-07 |
| TCGA-AA-A004-01A-01R-A00A-07 |
| TCGA-AA-A01P-01A-21R-A083-07 |
| TCGA-D5-6537-01A-11R-1723-07 |
| TCGA-D5-6532-01A-11R-1723-07 |
| TCGA-AA-3831-01A-01R-0905-07 |
| TCGA-CM-6171-01A-11R-1653-07 |
| TCGA-AA-3970-01A-01R-1022-07 |
| TCGA-A6-2684-01A-01R-1410-07 |
| TCGA-AA-A02K-01A-03R-A32Y-07 |
| TCGA-DM-A1D9-01A-11R-A155-07 |
| TCGA-CM-4751-01A-02R-1839-07 |
| TCGA-G4-6315-01A-11R-1723-07 |
